# Supplementary material for: Healthcare professionals’ experiences of job satisfaction when providing person-centred care: a systematic review of qualitative studies
Source: BMJ Open. 2023 Jun 9;13(6):e071178. doi: 10.1136/bmjopen-2022-071178 (PMC10277035; doi:10.1136/bmjopen-2022-071178)
Supplement: Supplementary data [file bmjopen-2022-071178supp002.pdf]

BMJ Publishing Group Limited (BMJ) disclaims all liability and responsibility arising from any reliance placed on this supplemental material which has been supplied by the author(s)

Online Supplementary File 2 – Search strategy

**Search strategy**				
**Population**	**Exposure**	**Outcome**	**Countries**	**Study design**
“health personnel” “care personnel” “health care provider*” “care provider*” “health care worker*” “health care staff” “caregiver*” “care assistant*” “care aides” “nurse*” “nursing*” “physician*” “physiotherapist*” “physical therapist*” “psychologist*” “social worker*” “health care professional*” “nutritionist*” “case manager*” “medical staff” “general practitioner*”	“person centered” “person centeredness” “client centered” “patient centered” **“patient-centered care”** “relationship centered” “family centered” “patient focused” “person focused” “individualized care” “personalized care” “person-directed care” “individual centered”	**“perception*”** “experience*” “perspective*” **“attitude*”** “stress of conscience” **“job satisfaction” “absenteeism” “presenteeism” “occupational stress” “occupational health” “personnel turnover*” “sick leave” “stress, psychological”** “psychological stress” **“dyssomnia*”** “sleep disorder*” “sleep disturbance*” “moral stress” **“attitude of health personnel”**	“Sweden” “Swedish” “Norway” “Norwegian” “Finland” “Finnish” “Denmark” “Danish” “Iceland” “Icelandic” “United kingdom” “UK” “British” “North Ireland” “North Irish” “Wales” “Welsh” “Scotland” “Scottish” “Ireland” “Irish” “the Netherlands” “Dutch” “Germany” “German” “Spain” “Spanish” “Italy” “Italian” “Belgium” “Belgian” “Portugal” “Portuguese” “Malta” “Maltese” “Estonia” “Estonian” “Slovenia” “Slovenian” “Czech republic” “Czech” “France” “French” “Poland” “polish” “Hungary”	**“qualitative research”** “qualitative stud*” **“focus group*” “grounded theory”** “mixed-method” “ethnography” “phenomenology*” **“interviews as topic”** “interview*” **“hermeneutic*”**

	“Hungarian” “Greece” “Greek” “Latvia” “Latvian” “Serbia” “Serbian”

PubMed 1885

Scopus 1620

Cinahl 249

PubMed searched 21 December 2021

(Sweden OR Swedish OR Norway OR Norwegian OR Finland OR Finnish OR Denmark OR Danish OR Iceland OR Icelandic OR “United Kingdom” OR UK OR British OR North Ireland OR North Irish OR Wales OR Welsh OR Scotland OR Scottish OR Ireland OR Irish OR The Netherlands OR Netherlands OR Dutch OR Germany OR German OR Spain OR Spanish OR Italy OR Italian OR Belgium OR Belgian OR Portugal OR Portuguese OR Malta OR Maltese OR Estonia OR Estonian OR Slovenia OR Slovenian OR Czech Republic OR Czech OR France OR French OR Poland OR Polish OR Hungary OR Hungarian OR Greece OR Greek OR Latvia OR Latvian OR Serbia OR Serbian)

AND

(“health personnel”[MeSH] OR “health personnel”[tiab] OR “care personnel”[tiab] OR “health care provider”[tiab] OR “health care providers”[tiab] OR “care provider”[tiab] OR “care providers”[tiab] OR “health care worker”[tiab] OR “health care staff”[tiab] OR caregiver[tiab] OR caregivers[tiab] OR caregivers[mesh] OR “healthcare worker”[tiab] OR “healthcare workers”[tiab] OR “care assistant”[tiab] OR “care aides”[tiab] OR nurses[mesh] OR nurse[tiab] OR nurses[tiab] OR nursing[tiab] OR physicians[mesh] OR physicians[tiab] OR physician[tiab] OR physiotherapists[tiab] OR “physical therapists”[mesh] OR “physical therapists”[tiab] OR psychologist[tiab] OR “social workers”[mesh] OR “social workers”[tiab] OR “social worker”[tiab] OR “Health care professionals”[tiab] OR “health care professional”[tiab] OR “nutritionist”[tiab] OR nutritionists[mesh] OR “case managers” [tiab] OR “Medical staff”[mesh] OR “Medical staff”[tiab] OR “general practitioners”[tiab] OR “General Practitioners”[mesh])

AND

(“Person centered”[tiab] OR “person centred”[tiab] OR “person-centred”[tiab] OR “person centeredness”[tiab] OR “personcenteredness”[tiab] OR “person centredness”[tiab] OR “client centered”[tiab] OR “client centred”[tiab] OR “patient centered”[tiab] OR “patient centred”[tiab] OR “Patient-Centered Care”[Mesh] OR “relationship centered”[tiab] OR “relationship centred”[tiab] OR “family centred”[tiab] OR “patient focused”[tiab] OR “person

focused"[tiab] OR "individualized care" [tiab] OR "personalized care" [tiab] OR "person-directed care" [tiab] OR "individual centered"[tiab])

AND

(perception[mesh] OR perception[tiab] OR experience[tiab] OR experiences[tiab] OR perspective[tiab] OR attitude[mesh] OR attitude[tiab] OR attitudes[tiab] OR "stress of conscience"[tiab] OR "Job Satisfaction"[Mesh] OR "job satisfaction"[tiab] OR "Absenteeism"[Mesh] OR "absenteeism"[tiab] OR "presenteeism"[Mesh] OR "presenteeism"[tiab] OR "Occupational Stress"[Mesh] OR "occupational stress"[tiab] OR "Personnel Turnover"[Mesh] OR "personnel turnover"[tiab] OR "Sick Leave"[Mesh] OR "sick leave"[tiab] OR "Stress, Psychological"[Mesh] OR "psychological stress"[tiab] OR "Dyssomnias"[Mesh] OR "sleep disorder"[tiab] OR "sleep disorders"[tiab] OR "sleep disturbances"[tiab] OR "occupational health"[Mesh] OR "occupational health"[tiab] OR "moral stress"[tiab] OR "Attitude of Health Personnel"[mesh])

AND

("qualitative research"[mesh] OR "qualitative research"[tiab] OR "qualitative study"[tiab] OR "qualitative studies"[tiab] OR "focus groups"[mesh] OR "focus groups"[tiab] OR "focus group"[tiab] OR "Grounded theory"[mesh] OR "grounded theory"[tiab] OR "mixed-method"[tiab] OR ethnography[tiab] OR phenomenology*[tiab] OR interview*[tiab] OR "interviews as topic"[mesh] OR Hermeneutics[mesh] OR hermeneutic*[tiab])

Scopus searched 21 December 2021

(sweden OR swedish OR norway OR norwegian OR finland OR finnish OR denmark OR danish OR iceland OR icelandic OR "United Kingdom" OR uk OR british OR "North Ireland" OR "North Irish" OR wales OR welsh OR scotland OR scottish OR ireland OR irish OR the AND netherlands OR netherlands OR dutch OR germany OR german OR spain OR spanish OR italy OR italian OR belgium OR belgian OR portugal OR portuguese OR malta OR maltese OR estonia OR estonian OR slovenia OR slovenian OR "Czech Republic" OR czech OR france OR french OR poland OR polish OR hungary OR hungarian OR greece OR greek OR latvia OR latvian OR serbia OR serbian) AND TITLE-ABS-KEY ("health personnel" OR "care personnel" OR "health care provider*" OR "care provider*" OR "health care worker*" OR "health care staff" OR caregiver* OR "healthcare worker*" OR "care assistant*" OR "care

aides" OR nurse* OR nursing OR physician* OR physiotherapist* OR "physical therapist*" OR psychologist* OR "social worker*" OR "Health care professional*" OR "nutritionist*" OR "case manager*" OR "Medical staff" OR "general practitioner*") AND TITLE-ABS-KEY ("Person centered" OR "person centred" OR "person centeredness" OR "personcenteredness" OR "person centredness" OR "client centered" OR "client centred" OR "patient centered" OR "patient centred" OR "Patient-Centered Care" OR "relationship centered" OR "relationship centred" OR "family centred" OR "patient focused" OR "person focused" OR "individualized care" OR "personalized care" OR "person-directed care" OR "individual centered") AND TITLE-ABS-KEY (perception* OR experience* OR perspective* OR attitude* OR "stress of conscience" OR "Job Satisfaction" OR absenteeism OR presenteeism OR "Occupational Stress" OR "Personnel Turnover*" OR "Sick Leave" OR "psychological stress" OR dyssomnia* OR "sleep disorder*" OR "sleep disturbance*" OR "occupational health" OR "moral stress") AND TITLE-ABS-KEY ("qualitative research" OR "qualitative stud*" OR "focus group*" OR "Grounded theory" OR "mixed-method" OR ethnography OR phenomenology OR interview* OR hermeneutic*) AND (LIMIT-TO (PUBYEAR , 2022) OR LIMIT-TO (PUBYEAR , 2021) OR LIMIT-TO (PUBYEAR , 2020) OR LIMIT-TO (PUBYEAR , 2019) OR LIMIT-TO (PUBYEAR , 2018) OR LIMIT-TO (PUBYEAR , 2017) OR LIMIT-TO (PUBYEAR , 2016) OR LIMIT-TO (PUBYEAR , 2015) OR LIMIT-TO (PUBYEAR , 2014) OR LIMIT-TO (PUBYEAR , 2013) OR LIMIT-TO (PUBYEAR , 2012) OR LIMIT-TO (PUBYEAR , 2011) OR LIMIT-TO (PUBYEAR , 2010))

Cinahl searched 21 December 2021

(Sweden OR Swedish OR Norway OR Norwegian OR Finland OR Finnish OR Denmark OR Danish OR Iceland OR Icelandic OR "United Kingdom" OR UK OR British OR "North Ireland" OR "North Irish" OR Wales OR Welsh OR Scotland OR Scottish OR Ireland OR Irish OR The Netherlands OR Netherlands OR Dutch OR Germany OR German OR Spain OR Spanish OR Italy OR Italian OR Belgium OR Belgian OR Portugal OR Portuguese OR Malta OR Maltese OR Estonia OR Estonian OR Slovenia OR Slovenian OR "Czech Republic" OR Czech OR France OR French OR Poland OR Polish OR Hungary OR Hungarian OR Greece OR Greek OR Latvia OR Latvian OR Serbia OR Serbian)

AND

((MH “health personnel+”) OR (TI “health personnel” OR AB “health personnel”) OR (TI “care personnel” OR AB “care personnel”) OR (TI “health care provider” OR AB “health care provider”) OR (TI “health care providers” OR AB “health care providers”) OR (TI “care provider” OR AB “care provider”) OR (TI “care providers” OR AB “care providers”) OR (TI “health care staff” OR AB “health care staff”) OR (TI caregiver OR AB caregiver) OR (TI caregivers OR AB caregivers) OR (MH caregivers+) OR (TI “healthcare worker” OR AB “healthcare worker”) OR (TI “healthcare workers” OR AB “healthcare workers”) OR (TI “care assistant” OR AB “care assistant”) OR (TI “care aides” OR AB “care aides”) OR (MH nurses+) OR (TI nursing OR AB nursing) OR (TI nurse OR AB nurse) OR (TI nurses OR AB nurses) OR (MH physicians+) OR (TI physicians OR AB physicians) OR (TI physician OR AB physician) OR (TI physiotherapists OR AB physiotherapists) OR (MH “physical therapists+”) OR (TI “physical therapists” OR AB “physical therapists”) OR (TI psychologist OR AB psychologist) OR (MH “social workers+”) OR (TI “social workers” OR AB “social workers”) OR (TI “social worker” OR AB “social worker”) OR (TI “Health care professionals” OR AB “Health care professionals”) OR (TI “health care professional” OR AB “health care professional”) OR (TI nutritionist OR AB nutritionist) OR (TI “case managers” OR AB “case managers”) OR (MH “Medical staff+”) OR (TI “Medical staff” OR AB “Medical staff”) OR (TI “general practitioners” OR AB “general practitioners”) OR (MH “Physicians, Family+”))

AND

((TI “Person centered” OR AB “Person centered”) OR (TI “person centred” OR AB “person centred”) OR (TI “person centeredness” OR AB “person centeredness”) OR (TI personcenteredness OR AB personcenteredness) OR (TI “person centredness” OR AB “person centredness”) OR (TI “client centered” OR AB “client centered”) OR (TI “client centred” OR AB “client centred”) OR (TI “patient centered” OR AB “patient centered”) OR (TI “patient centred” OR AB “patient centred”) OR (MH “Patient Centered Care”) OR (TI “relationship centered” OR AB “relationship centered”) OR (TI “relationship centred” OR AB “relationship centred”) OR (TI “family centred” OR AB “family centred”) OR (TI “patient focused” OR AB “patient focused”) OR (TI “person focused” OR AB “person focused”) OR (TI “individualized care” OR AB “individualized care”) OR (TI “personalized care” OR AB “personalized care”) OR (TI “person-directed care” OR AB “person-directed care”) OR (TI “individual centered” OR AB “individual centered”)) AND ((MH perception+) OR (TI perception OR AB perception) OR (TI experience OR AB experience) OR (TI

experiences OR AB experiences) OR (TI perspective OR AB perspective) OR (MH attitude+) OR (TI attitude OR AB attitude) OR (TI attitudes OR AB attitudes) OR (TI “stress of conscience” OR AB “stress of conscience”) OR (MH “Job Satisfaction+”) OR (TI “job satisfaction” OR AB “job satisfaction”) OR (MH Absenteeism+) OR (TI absenteeism OR AB absenteeism) OR (MH presenteeism+) OR (TI presenteeism OR AB presenteeism) OR (MH “Stress, Occupational+”) OR (TI “occupational stress” OR AB “occupational stress”) OR (MH “Personnel Turnover+”) OR (TI “personnel turnover” OR AB “personnel turnover”) OR (MH “Sick Leave+”) OR (TI “sick leave” OR AB “sick leave”) OR (MH “Stress, Psychological+”) OR (TI “psychological stress” OR AB “psychological stress”) OR (MH Dyssomnias+) OR (TI “sleep disorder” OR AB “sleep disorder”) OR (TI “sleep disorders” OR AB “sleep disorders”) OR (TI “sleep disturbances” OR AB “sleep disturbances”) OR (MH “occupational health+”) OR (TI “occupational health” OR AB “occupational health”) OR (TI “moral stress” OR AB “moral stress”) OR (MH “Attitude of Health Personnel+”))

AND

((MH “qualitative studies+”) OR (TI “qualitative research” OR AB “qualitative research”) OR (TI “qualitative study” OR AB “qualitative study”) OR (TI “qualitative studies” OR AB “qualitative studies”) OR (MH “focus groups+”) OR (TI “focus groups” OR AB “focus groups”) OR (TI “focus group” OR AB “focus group”) OR (MH “Grounded theory+”) OR (TI “grounded theory” OR AB “grounded theory”) OR (TI mixed-method OR AB mixed-method) OR (TI ethnography OR AB ethnography) OR (TI phenomenology* OR AB phenomenology*) OR (TI interview* OR AB interview*) OR (MH “interviews+”) OR (MH “Phenomenology”) OR (TI hermeneutic* OR AB hermeneutic*))
