# Supplementary material for: Healthcare professionals’ experiences of job satisfaction when providing person-centred care: a systematic review of qualitative studies
Source: BMJ Open. 2023 Jun 9;13(6):e071178. doi: 10.1136/bmjopen-2022-071178 (PMC10277035; doi:10.1136/bmjopen-2022-071178)
Supplement: Supplementary data [file bmjopen-2022-071178supp003.pdf]

Online Supplementary File 3 – Screening Tool

Screening Tool

Citation, Title, and Abstract Screening

1. Does the **citation** indicate publication on or after 2010?
    a. Yes or Unsure/Unclear: continue screening
    b. No: stop screening

2. Does the **title or abstract** use English?
    a. Yes or Unsure/Unclear: continue screening
    b. No: stop screening

3. Does the **title or abstract** indicate that the study uses a qualitative design?
    a. Yes or Unsure/Unclear: continue screening
    b. No: stop screening

Keywords: qualitative, interview, focus group, grounded-theory, mixed-method, ethnography, phenomenology, hermeneutic.

4. Does the **title or abstract** indicate that the study concerns PCC?
    a. Yes or Unsure/Unclear: continue screening
    b. No: stop screening

Note: This also includes the different definitions of PCC, e.g., Patient-Centred Care, Relationship-Centred Care, Client-Centred Care etc. (See block 3 in Search Strategy).

5. Does the **title or abstract** indicate that the study concerns HCPs perspectives/experiences of PCC?
    a. Yes or Unsure/Unclear: continue screening
    b. No: stop screening

6. Does the **title or abstract** indicate that the research was conducted in one of the countries from the inclusion criteria?

   a. Yes or Unsure/Unclear: continue screening

   b. No: stop screening

Countries: Sweden, Norway, Finland, Denmark, Iceland, United Kingdom (England, Scotland, Wales, North Ireland) Ireland, The Netherlands, Germany, Spain, Italy, Belgium, Portugal, Malta, Estonia, Slovenia, Czech Republic, France, Poland, Hungary, Greece, Latvia, Serbia.

7. Does the **title or abstract** indicate that job satisfaction/occupational health in relation to PCC was studied?

   a. Yes or Unsure/Unclear: continue screening

   b. No: stop screening

Keywords: satisfaction, job satisfaction, job strain, stress, moral stress, stress of conscience, demand, occupational health

Decision: **Should this article be included for full-text read?**

   a. **Yes**, all 7 screening questions answered Yes or Unsure/Unclear

   b. **No**, at least one answers definitely “No”
