# Supplementary material for: Healthcare professionals’ experiences of job satisfaction when providing person-centred care: a systematic review of qualitative studies
Source: BMJ Open. 2023 Jun 9;13(6):e071178. doi: 10.1136/bmjopen-2022-071178 (PMC10277035; doi:10.1136/bmjopen-2022-071178)
Supplement: Supplementary data [file bmjopen-2022-071178supp004.pdf]

Online Supplementary File 4 – SBU Checklist

Bedömning av studier med kvalitativ metodik

UPPDATERAD 2020-10-06

Författare: ______________ År: ______________

Granskare: ______________

Sammanvägd bedömning av metodologiska brister:
Obetydliga eller mindre ☐<br>Måttliga ☐
Stora brister, studien ingår inte i syntesen ☐

Kommentarer:

1. Överensstämmelse mellan filosofisk hållning/teori och urval och metodik i studien[1]

Vilken teori eller filosofisk hållning utgick författarna från?

	Ja	Nej	Oklart
Hänger syfte och fråga ihop med teori/filosofisk hållning?	☐	☐	☐

Kommentarer:

2. Deltagare

Hur gjordes urvalet?

**Stödfrågor för bedömning av brister i urvalsförfarandet:**	**Ja**	**Nej**	**Oklart**
Är urvalet lämpligt för att besvara frågan?	☐	☐	☐
Är rekryteringsmetoden lämpligt vald och genomförd?	☐	☐	☐
Finns det allvarliga brister som kan påverka tillförlitligheten?	☐	☐	☐

Kommentarer:

3. Datainsamling

Vilka metoder användes för datainsamling?

	Ja	Nej	Oklart
Finns det allvarliga brister i datainsamlingen som kan påverka tillförlitligheten?	☐	☐	☐

Kommentarer:

4. Analys

Vilka metoder användes för analys?

Stödfrågor för bedömning av brister i analyssteget:	Ja	Nej	Oklart
Är vald analysmetod lämplig och genomförd på ett lämpligt sätt?	☐	☐	☐
Var forskarna reflexiva vid tolkning av data?	☐	☐	☐
Validerades tolkningarna?	☐	☐	☐
Finns det allvarliga brister i analysen som kan påverka tillförlitligheten?	☐	☐	☐

Kommentarer:

5. Forskaren

Vilken bakgrund och kompetens hade forskarna?

Stödfrågor för bedömning av brister:	Ja	Nej	Oklart
Har forskarna någon relation till studiedeltagarna som kan påverka datainsamlingen?	☐	☐	☐
Har forskarna hanterat sin förförståelse på ett acceptabelt sätt?	☐	☐	☐
Var forskarna oberoende av finansiella eller andra förutsättningar som kunde påverka analysen?	☐	☐	☐
Finns det allvarliga brister som kan påverka tillförlitligheten?	☐	☐	☐

Kommentarer:
