# Supplementary material for: Healthcare professionals’ experiences of job satisfaction when providing person-centred care: a systematic review of qualitative studies
Source: BMJ Open. 2023 Jun 9;13(6):e071178. doi: 10.1136/bmjopen-2022-071178 (PMC10277035; doi:10.1136/bmjopen-2022-071178)
Supplement: Supplementary data [file bmjopen-2022-071178supp005.pdf]

Online Supplementary File 5 – Quality Assessments

Quality assessments from the SBU checklist

Study	Data collection	Analysis	1. Theory/Philosophy: aim and research question coherent with theory or philosophical approach	2. Participants: relevant sampling; recruitment relevant and well described; ethical considerations relevant and well described	3. Data collection: relevant and well described	4. Data analysis: methodology relevant and well described; researchers showing reflexiveness in their analysis	5. The researcher: researcher–participant relationship well described; researcher pre-understanding well described; conflict of interest described	Methodological quality assessment
Barbosa *et al*[64]	Semi-structured focus groups	Thematic analysis	Yes	Yes	Yes/unclear	Yes	Yes	High
				Limitations: Ensure participants confidentiality and anonymity, but conducts focus groups where that cannot be ensured. Only one moderator for focus groups. Brief description of analysis.				
Boersma *et al*[61]	Focus groups	Deductive analysis from the RE-AIM framework	Yes	Yes	Yes/unclear	Yes	Yes/no	Moderate
				Limitations: Researcher-participant relation not described. Potential response bias discussed (with regards to focus group).				

Boström *et al*[50]	Focus group and individual semi-structured interviews	Qualitative content analysis	Yes	Yes	Yes	Yes	Yes/no	Moderate
			Limitations: Researcher-participant relation not described. Not discussing preunderstanding.					
Coyne[63]	Individual interviews with open-ended questions	Grounded theory	Yes	Yes	Unclear	Yes	Yes/no	Moderate
			Limitations: Researcher-participant relation not described. Split focus in the data between families/patients and HCPs.					
Fridberg *et al*[51]	Focus groups, semi-structured dyadic interviews, and semi-structured individual interviews	Deductive–inductive content analysis	Yes	Yes	Unclear	Yes	Yes	Moderate
			Limitations: Potential data collection bias.					
Kadri *et al*[57]	Semi-structured individual interviews	Secondary qualitative thematic analysis	Yes	Yes	Unclear	Yes	Yes/no	Moderate
			Limitations: Initial study not having the precise focus relevant for this study which means data could have been more precise, the secondary analysis however relevant for this review. Preunderstanding not described.					
Karlsson *et al*[52]	Semi-structured individual interviews	Systematic text condensation	Yes	Yes	Yes	Yes	Yes/no	High
			Limitations: Declaration of interest not described.					

Kirkley *et al*[60]	Focus groups, telephone interview, individual interviews	Qualitative thematic analysis	Yes	Yes/no	Yes	Yes	Yes/no	Moderate
			Limitations: Potential selection bias in the face-to-face interviews. Participants worked in departments with good knowledge in PCC whereas in the telephone interviews the knowledge varied. Relation with participants not described.					
Kjörnsberg *et al*[54]	Open-ended informal individual interviews	Latent qualitative content analysis	Yes	Yes/no	Yes	Yes	Yes/no	Moderate
			Limitations: Small sample of only 6 RNs. Conflict of interest not described.					
Nilsson *et al*[53]	Individual semi-structured interviews	Qualitative content analysis	Yes	Yes	Yes	Yes	Yes/no	Moderate
			Limitations: Researcher-participant relationship not described. Preunderstanding not described.					
Pinkert *et al*[65]	Semi-structured focus groups	Secondary qualitative content analysis	Yes	Yes	Yes/unclear	Yes	Yes/no	Moderate
			Limitations: Cannot find researcher-participant relation due to not getting access to the initial study. Only focus groups.					
Ross *et al*[58]	Individual semi-structured interviews	Framework analysis	Yes	Yes	Yes	Yes	Yes/no	High
			Limitations: Researcher-participant relationship unclear and potential bias not described.					

Sjöberg and Forsner[55]	Focus groups, semi-structured individual interviews	Analysis in phenomenography	Yes	Yes/unclear	Yes	Yes	Yes/no	Moderate
			Limitations: Relatively low sample size (n = 7). Researcher-participant relationship not described.					
Uittenbroek *et al*[62]	Individual interviews with topic-based interview guide	Grounded theory	Yes	Yes/no	Yes	Yes	Yes/no	High
			Limitations: All participants were women. Researcher-participant relationship not described.					
Vassbø *et al*[66]	Individual open-ended interviews	Phenomenological-hermeneutical analysis for research of lived experiences	Yes	Yes/no	Yes	Yes	Yes/no	Moderate
			Limitations: Almost all participants were women. Relationship between some researchers and participants that could lead to potential bias.					
Walker and Deacon[59]	Group interviews and an individual interview	Qualitative directed content analysis	Yes	Yes	Yes/no	Yes	Yes/no	Moderate
			Limitations: Researcher-participant relationship not described.					
Öhman *et al*[56]	Individual interviews with thematic interview guide	Grounded theory, qualitative content analysis	Yes	Yes	Yes	Yes	Yes/no	High
			Limitations: Preunderstanding not described. Researcher-participant relationship not described.					
