# Supplementary material for: Healthcare professionals’ experiences of job satisfaction when providing person-centred care: a systematic review of qualitative studies
Source: BMJ Open. 2023 Jun 9;13(6):e071178. doi: 10.1136/bmjopen-2022-071178 (PMC10277035; doi:10.1136/bmjopen-2022-071178)
Supplement: Supplementary data [file bmjopen-2022-071178supp006.pdf]

Online Supplementary File 6 - Characteristics of included studies

Author, year, country	Aim	Data collection method	Analysis method	Context	HCPs
Barbosa *et al*,[64] 2015, Portugal	To explore direct-care workers' experiences of a psychoeducational intervention including person-centred care	Semi-structured focus groups (mixed methods)	Thematic analysis	Four residential care facilities	Direct-care workers (n = 50) (8 focus groups) (all women)
Boersma *et al*,[61] 2017, The Netherlands	To explore how caregivers working with a person-centred care method (the Veder method) experience their job satisfaction	Focus groups (mixed methods)	Deductive analysis from the RE-AIM framework	Six nursing home wards	Professional caregivers (n = 42) (6 focus groups) (40 women, 2 men)
Boström *et al*,[50] 2014, Sweden	To describe how nurses specialising in diabetes care experience patient-centred care during an intervention for type 2 diabetes	Focus group and individual semi-structured interviews	Qualitative content analysis	Primary health care centres in northern Sweden	Diabetes specialist nurses (n = 10) (2 focus groups, 10 individual interviews, same participants) (all women)
Coyne,[63] 2015, Ireland	To explore families' and nurses' experiences and perceptions of family-centred care	Individual interviews using open-ended questions	Constructivist grounded theory	One general hospital ward and one surgical ward for children in a children's hospital, and one surgical	RNs (n = 18) (17 women, 1 man)

				ward for children in a general district hospital	
Fridberg *et al*,[51] 2021, Sweden	To describe HCPs' perceptions and experiences of PCC implementation	Focus groups, semi-structured dyadic interviews, and semi-structured individual interviews	Deductive–inductive content analysis	Six healthcare units in a region in central Sweden	97 in total<br>Assistant nurses (*n* = 30), RNs (*n* = 40), physicians (*n* = 10), physiotherapists (*n* = 9), occupational therapists (*n* = 7), 'other' (not specified) (*n* = 1)<br>(65 participants in 15 focus groups, 10 participants in 5 dyadic interviews, 22 individual interviews)<br>(80 women, 17 men)
Kadri *et al*,[57] 2018, UK	To explore home-care staff members' experiences and perceptions of their personhood when working with person-centred care	Semi-structured individual interviews	Secondary qualitative thematic analysis	Six care homes in the UK: three residential care homes and three nursing homes	Care home staff (*n* = 25)<br>(17 women, 8 men)
Karlsson *et al*,[52] 2019, Sweden	To describe RNs' job satisfaction	Semi-structured individual interviews	Systematic text condensation	One university hospital and one county hospital from different healthcare regions in Sweden	RNs (*n* = 25)<br>(23 women, 2 men)
Kirkley *et al*,[60] 2011, UK: England, Scotland	To explore cultural organisational perspectives in the provision of person-centred care	Focus groups, individual interviews (mixed methods)	Qualitative thematic analysis	Organisations in England and Scotland providing respite care	Frontline staff (*n* = 17)

Kjörnsberg *et al*,[54] 2010, Sweden	To investigate RNs' experiences of transitioning to patient-focused care	Open-ended informal individual interviews	Latent qualitative content analysis	A ward for infectious diseases at a university hospital in central Sweden	RNs (*n* = 6) (all women)
Nilsson *et al*,[53] 2019, Sweden	To explore RNs' experiences of working with person-centred care	Individual semi-structured interviews, focus groups	Qualitative content analysis	An acute ward at a university hospital in Sweden	14 in total RNs, enrolled nurses (12 women, 2 men)
Pinkert *et al*,[65] 2018, Austria and Germany	To describe the experiences of RNs working with dementia patients in acute-care hospital contexts	Semi-structured focus groups	Secondary qualitative content analysis	Acute-care settings in four Austrian hospitals and five German hospitals	RNs (*n* = 68) RNs from Austrian wards (*n* = 46) and German wards (*n* = 22) (12 focus groups)
Ross *et al*,[58] 2015, UK	To describe nurses' perspectives on facilitators of person-centred care	Individual semi-structured interviews	Framework analysis	An acute ward at a UK teaching hospital	14 in total RNs (*n* = 7), support workers (*n* = 3), student nurses (*n* = 4)
Sjöberg and Forsner,[55] 2020, Sweden	To explore physiotherapists' varying perceptions of person-centred care	Focus groups, semi-structured individual interviews	Analysis in phenomenography in accordance with Sjöström and Dahlgren (2002)	Wards for acute medicine, rheumatology, surgery, and orthopaedics	Physiotherapists (*n* = 7) (7 individual interviews, 1 focus group with 3 participants from individual interviews) (6 women, 1 man)
Uittenbroek *et al*,[62] 2018, The Netherlands	To explore case managers' perceptions of their professional role when working with person-centred care	Individual interviews using topic-based interview guide	Classical grounded theory	GP practices in northern Netherlands	11 in total RNs (*n* = 6), social workers (*n* = 5) (All women)

Vassbø *et al*,[66] 2019, Australia, Norway, and Sweden	To illuminate how HCPs in nursing homes experience the meaning of person-centred care focusing on their job satisfaction	Individual open-ended interviews	Phenomenological–hermeneutical analysis for research into lived experiences	Three nursing homes: one in a city in Norway, one in rural Sweden, and one in rural Australia	29 in total<br>RNs (*n* = 10), enrolled nurses (*n* = 12), care assistants (*n* = 2), personal carers (*n* = 2), occupational therapists (*n* = 2), physiotherapist (*n* = 1)
Walker and Deacon,[59] 2016, UK	To explore nurses' experiences of person-centred care of the suddenly bereaved	Group interviews, dyadic interview, and individual interview	Qualitative directed content analysis	A hospital with critical and acute-care services	RNs (*n* = 9)<br>(2 group interviews with 7 participants, 1 dyadic and 1 individual interview)
Öhman *et al*,[56] 2017, Sweden	To explore and improve knowledge of the factors contributing to HCPs' job satisfaction	Individual interviews using thematic interview guide (mixed methods)	Constructivist grounded theory, qualitative content analysis	Six elderly-care facilities in Sweden	17 in total<br>RNs (*n* = 5), physiotherapists (*n* = 6), occupational therapists (*n* = 6)<br>(14 women, 3 men)
