# Supplementary material for: Healthcare professionals’ experiences of job satisfaction when providing person-centred care: a systematic review of qualitative studies
Source: BMJ Open. 2023 Jun 9;13(6):e071178. doi: 10.1136/bmjopen-2022-071178 (PMC10277035; doi:10.1136/bmjopen-2022-071178)
Supplement: Author's manuscript [file bmjopen-2022-071178.draft_revisions.pdf]

BMJ Open is committed to open peer review. As part of this commitment we make the peer review history of every article we publish publicly available.

When an article is published we post the peer reviewers' comments and the authors' responses online. We also post the versions of the paper that were used during peer review. These are the versions that the peer review comments apply to.

The versions of the paper that follow are the versions that were submitted during the peer review process. They are not the versions of record or the final published versions. They should not be cited or distributed as the published version of this manuscript.

BMJ Open is an open access journal and the full, final, typeset and author-corrected version of record of the manuscript is available on our site with no access controls, subscription charges or pay-per-view fees (http://bmjopen.bmj.com).

If you have any questions on BMJ Open's open peer review process please email info.bmjopen@bmj.com

BMJ Open

Healthcare professionals' experiences of job satisfaction when providing person-centred care: a systematic review of qualitative studies from Europe

Journal:	*BMJ Open*
Manuscript ID	bmjopen-2022-071178
Article Type:	Original research
Date Submitted by the Author:	17-Dec-2022
Complete List of Authors:	Gustavsson, Kristoffer; University of Gothenburg Centre for Person-Centred Care<br>van Diepen, Cornelia; University of Gothenburg Centre for Person-Centred Care; Erasmus Universiteit Rotterdam Erasmus School of Health Policy and Management,<br>Fors, Andreas; University of Gothenburg Centre for Person-Centred Care; University of Gothenburg Institute of Health and Care Sciences<br>Axelsson, Malin; Malmö University Department of Care Science<br>Bertilsson, Monica; University of Gothenburg School of Public Health and Community Medicine<br>Hensing, Gunnel; University of Gothenburg School of Public Health and Community Medicine
Keywords:	PREVENTIVE MEDICINE, PUBLIC HEALTH, Quality in health care < HEALTH SERVICES ADMINISTRATION & MANAGEMENT, SOCIAL MEDICINE

SCHOLARONE™
Manuscripts

I, the Submitting Author has the right to grant and does grant on behalf of all authors of the Work (as defined in the below author licence), an exclusive licence and/or a non-exclusive licence for contributions from authors who are: i) UK Crown employees; ii) where BMJ has agreed a CC-BY licence shall apply, and/or iii) in accordance with the terms applicable for US Federal Government officers or employees acting as part of their official duties; on a worldwide, perpetual, irrevocable, royalty-free basis to BMJ Publishing Group Ltd ("BMJ") its licensees and where the relevant Journal is co-owned by BMJ to the co-owners of the Journal, to publish the Work in this journal and any other BMJ products and to exploit all rights, as set out in our licence.

The Submitting Author accepts and understands that any supply made under these terms is made by BMJ to the Submitting Author unless you are acting as an employee on behalf of your employer or a postgraduate student of an affiliated institution which is paying any applicable article publishing charge ("APC") for Open Access articles. Where the Submitting Author wishes to make the Work available on an Open Access basis (and intends to pay the relevant APC), the terms of reuse of such Open Access shall be governed by a Creative Commons licence – details of these licences and which Creative Commons licence will apply to this Work are set out in our licence referred to above.

Other than as permitted in any relevant BMJ Author's Self Archiving Policies, I confirm this Work has not been accepted for publication elsewhere, is not being considered for publication elsewhere and does not duplicate material already published. I confirm all authors consent to publication of this Work and authorise the granting of this licence.

For peer review only

Title: Healthcare professionals' experiences of job satisfaction when providing person-centred care: a systematic review of qualitative studies from Europe

Kristoffer Gustavsson (ORCID id: 0000-0002-1743-9576),[1] Cornelia van Diepen (0000-0001-6991-9443),[1,2] Andreas Fors (0000-0001-8980-0538),[1,3,4] Malin Axelsson (0000-0001-5493-8334),[5] Monica Bertilsson (0000-0003-2365-2522),[6] Gunnel Hensing (0000-0002-3457-2993)[6]

[1] Centre for Person-Centred Care (GPCC), University of Gothenburg, Gothenburg, Sweden

[2] Erasmus School of Health Policy & Management, Erasmus University Rotterdam, Rotterdam, Netherlands

[3] Institute of Health and Care Sciences, Sahlgrenska Academy, University of Gothenburg, Goteborg, Sweden

[4] Region Västra Götaland, Research, Education, Development and Innovation, Primary Health Care, Gothenburg, Sweden

[5] Department of Care Science, Faculty of Health and Society, Malmö University, Malmö, Sweden

[6] School of Public Health and Community Medicine, Institute of Medicine, Sahlgrenska Academy, University of Gothenburg, Gothenburg, Sweden

Corresponding author:

Andreas Fors

Andreas.fors@gu.se

University of Gothenburg, Sahlgrenska Academy, Institute of Health and Care Sciences

Arvid Wallgrens backe, building 1

Box 457, 405 30 Gothenburg, Sweden

Word count: 3982 (excluding title page, abstract, references, figures and tables)

For peer review only

ABSTRACT

Objectives: This qualitative systematic review aimed to explore and synthesise healthcare professionals' (HCPs) experiences of job satisfaction when providing person-centred care (PCC) in healthcare settings in Europe.

Method: This systematic review of qualitative studies was followed by a thematic synthesis applying an inductive approach. Studies concerning HCPs and different levels of healthcare in Europe were eligible for inclusion. The Cinahl, Pubmed, and Scopus databases were searched. Study titles, abstracts, and full texts were screened for relevance. Included studies were assessed for methodological quality using a quality appraisal checklist. Data were extracted and synthesised via thematic synthesis, generating analytical themes.

Results: Seventeen studies were included in the final thematic synthesis, and eight analytical themes were derived. Most studies were conducted in Sweden and the United Kingdom and were performed in hospitals, nursing homes, elderly care, and primary care. Thirteen of these studies were qualitative and four used a mixed-method design in which the qualitative part was used for analysis. HCPs experienced challenges adapting to a new remoulded professional role and felt torn and inadequate due to ambiguities between organisational structures, task-oriented care, and PCC. Improved job satisfaction was experienced when providing PCC in line with ethical expectations, patients and colleagues expressed appreciation, and team collaboration improved, while learning new skills generated motivation.

Conclusion: This systematic review found varied experiences among HCPs. Notably, the new professional role was experienced to entail disorientation and uncertainty; importantly, it also entailed meaningfulness, an improved relationship between HCPs and patients, appreciation, and collaboration. To facilitate PCC implementation, healthcare organisations should focus on supporting HCPs in their new roles.

PROSPERO registration number: CRD42022304732

For peer review only

ARTICLE SUMMARY

Strengths and limitations of this study:

- This is the first systematic review synthesising qualitative research on healthcare professionals' experiences of job satisfaction when providing person-centred care across different healthcare settings in Europe.
- The methodology was thoroughly described, increasing replicability and reliability, and the researchers involved in this review had different professional backgrounds, i.e., nursing, sociology, and social medicine.
- The search strategy was extensive, included a broad range of terms, and covered several databases; a search specialist was consulted in the process.
- The search was limited to studies written in English and published in or since 2010, so some potentially relevant studies might have been missed.
- The included studies had different qualitative methodologies and theoretical approaches, which could affect interpretations and comparisons.

Keywords: healthcare professional, job satisfaction, person-centred care, qualitative synthesis

For peer review only

INTRODUCTION

In Europe, healthcare professionals (HCPs) are at higher risk of exposure to psychosocial risk factors from their work environment than are several other occupations,[1] which can result in adverse health impacts on the individual, such as stress, fatigue, burnout, and physical and emotional demands.[2] The working environment affects job satisfaction, which is a mental state of satisfaction deriving from employees' assessment of their work situation.[3] It includes factors such as accomplishment, praise, social relations, advancement opportunities, and undertaking tasks in line with personal ethical values.[4 5] Low job control can decrease health and job satisfaction,[6] and high stress combined with decreased job satisfaction is associated with high turnover rates in healthcare.[7] With demands on European healthcare systems gradually increasing,[8] recruiting and retaining educated HCPs is difficult, threatening the sustainability of healthcare.[9] Moreover, an under-supported workplace with many stressors can lead to decreased quality of care for patients and increased emotional demands on HCPs.[10]

Simultaneously, European healthcare has been evolving to involve more patient participation.[11] Person-centred care (PCC) is a model of care deriving from an ethical standpoint in which patients' capabilities and needs are emphasised, and care is provided through collaboration between professionals, patients, and family.[12 13] Providing PCC involves some key principles, such as: initiating and focusing partnership by carefully listening to the patient's narrative, with the patient seen as an expert on her/his health; and co-creating a health plan covering the patient's experiences, capabilities, goals, expectations, and social support resources along with medical status, and modifying the health plan according to the circumstances.[12 14 15] PCC communication involves both verbal (e.g., open-ended questions, reflections, and summaries) and non-verbal communication (e.g., displaying welcoming and respectful body language).[14 16]

Working with PCC based on the patient's narrative helps professionals realise their expected ethical standards and provide high-quality care.[17] Working in a more person-centred way requires conducive organisational structure and support, leadership and training, and constructive collaboration within the interprofessional team.[18 19] Several concepts have been used to describe care centred around the patient and her/his family, such as patient centredness or patient-centred care[20 21] or relationship-centred,[22] family-centred,[23] and

individualised care,[24] and varied settings require specific approaches of ‘centredness’ on the patient and family.[25 26] PCC entails building a relationship in which patients are involved as active partners in their care and treatment and, more distinctly, emphasises a shift away from a model of care in which patients are often regarded as passive care recipients.[13 15]

In European healthcare, ethical stress and lack of resources are issues affecting HCPs’ ability to offer the desired care of the required quality.[27] PCC has been proposed as a model of care with potential to help solve some of these problems, although evidence regarding PCC and its impact on HCP outcomes remains unclear.[28] Enhancing our understanding of how HCPs’ job satisfaction is related to PCC could lead to further research, new relevant policies, and improved working conditions for HCPs. Research has primarily investigated the patient outcomes of PCC, showing, for example, increased self-efficacy, improved satisfaction with care, improved symptom control, and clinical outcomes characterised by shorter hospital stays and cost savings.[29] Five reviews of aspects of HCP outcomes and PCC have been identified,[30-34] which presented mixed findings. These reviews focused on specific professions, such as registered nurses (RNs), nurse aides, direct-care workers, and caregivers in residential care or nursing homes,[30-32 34] or included only quantitative studies.[30 31 33 34] No reviews have explored qualitative research on job satisfaction from providing PCC across healthcare professions and settings in Europe; this systematic review accordingly aimed to fill this research gap.

AIM

This qualitative systematic review aimed to explore and synthesise HCPs’ experiences of job satisfaction when providing PCC in healthcare settings in Europe.

METHODS

Design

A systematic review of qualitative studies was conducted. The research question was ‘How do healthcare professionals in Europe, when providing PCC, experience their job satisfaction?’ A protocol following the PRISMA-P checklist was written in preparation for the systematic review and registered in PROSPERO on 23 January 2022 (ID: CRD42022304732).[35] A systematic search was executed on 21 December 2021 in the electronic databases Cinahl, Pubmed, and Scopus, covering the main research areas relevant to this review. Thematic synthesis with an inductive approach was used to analyse data obtained from the relevant studies.[36] The framework for Enhancing Transparency in Reporting the Synthesis of

Qualitative Research (ENTREQ)[37] was used for reporting the synthesis (see Online Supplementary File 1).

Eligibility criteria

The eligibility criteria for the literature search in this systematic review were based on the Population, Exposure, Outcome (PEO) framework.[38] The inclusion criterion for the population was HCPs, for exposure it was PCC, and for the outcome it was experiences of job satisfaction. Additionally, studies from all healthcare settings were acceptable, provided they were conducted in European countries with a healthcare system that could be identified according to Rosengren *et al*.[39] Studies had to be qualitative, peer-reviewed, ethically approved, written in English, and published in or since 2010. The included qualitative studies also include the qualitative, but no quantitative, parts of mixed-method studies. The exclusion criteria were quantitative studies and reviews.

Search strategy

The search strategy was created by the research group with assistance from a librarian at the Biomedical Library at the University of (name withheld during the review process). It comprised keywords and Medical Subject Heading (MeSH) terms hierarchically structured to facilitate the inclusion of broader and more precise medical and health-related terms.[40] In accordance with the PEO framework, relevant search terms associated with HCPs, PCC, and job satisfaction were defined and put in their associated search blocks.[38] Within each block, the search terms were connected using the Boolean operator 'OR', and the blocks were connected with the Boolean operator 'AND'.[41] Along with the PEO blocks, two additional blocks were added. The first additional search block, 'countries', consisted of the European countries eligible for inclusion, similar to Rosengren *et al*[39] and their mapping of European countries having identifiable and eligible healthcare systems. Rosengren *et al* found 23 eligible European countries and identified three types of healthcare systems: Beveridge (n = 12), Bismarck (n = 10), and Out of Pocket (n = 1).[39] The second additional block was 'study design', which comprised different search terms for qualitative study designs and mixed-method studies (e.g., interviews and focus groups) to ensure that the search would identify all relevant studies of qualitative design. The search strategy is presented in Online Supplementary File 2.

For peer review only

Selection process

The records from the database searches were imported into Endnote for reference management and into Rayyan software for screening. Duplicates were removed using the 'detect duplicates' option available in both programs.[42 43] The first (A1) and second author (A2) screened titles and abstracts blinded and independently for relevance in Rayyan using a screening tool (see Online Supplementary File 3).[44]

In the second step of the selection process, the remaining studies were downloaded and shared in an Endnote library. The studies were read in full text for relevance against the inclusion criteria by A1 and A2 independently in Endnote. All full-text studies were discussed in a meeting among A1, A2, and A3 until consensus was reached. Eighteen studies were considered relevant to the review. After closer reading, one study was interpreted as related to working during the COVID-19 pandemic rather than to person-centred work; therefore, it was excluded at this stage after discussions.

Quality assessment

The Swedish Agency for Health Technology Assessment and Assessment for Social Services (SBU) checklist for assessing methodological quality was used to assess the included studies after they were read in full text.[45] This checklist has five domains (i.e., theoretical framework, sampling, data collection, analysis, and researcher role) and 13 items integrated among the five domains (see Online Supplementary File 4).[46]

In line with SBU's recommendations, the studies were rated by A1, who considered omitting studies about which there were considerable methodological concerns, indicating low methodological quality.[47] The rating categories were as follows: no or few concerns = high quality; moderate concerns = moderate quality; and high concerns = low quality. The studies' ratings varied between moderate (n = 12) and high (n = 5) quality. The most common deficiencies were missing descriptions of the researcher's role and how it might have affected the study results. No studies were assessed as having low methodological quality, so no studies were excluded due to the methodological assessment. The quality assessments are presented in Online Supplementary File 5.

For peer review only

Data extraction and synthesis

The qualitative findings extracted from the included studies were analysed via thematic synthesis. The synthesis followed the three stages described by Thomas and Harden.[36] The synthesis was conducted by A1 supported by A6. All authors then reviewed and discussed the synthesis and themes.

The first stage of the thematic synthesis was coding, which was conducted using NVivo software, in which the text was coded line by line to identify passages of text from the studies in line with the aim and research question of the review. Each text passage received a code describing its meaning. The coding was continuously scrutinised: it was revised with further coding if consistency was lacking, or progressed without revision if no issues emerged.[36]

The second stage involved inductively developing descriptive themes from the data. In this stage, a descriptive theme was formulated to cover the first code from the first study; either the following codes from the same study were grouped under that theme, if the codes were related in meaning and concept, or new descriptive themes were formulated when codes with new thematic concepts emerged. Codes from subsequent studies were then assigned to existing descriptive themes or new themes were formulated, if needed.[36]

The third stage of the process 'went beyond' the content of the primary studies and created a new understanding of the topic. New knowledge was created by utilising the descriptive themes generated and connecting them to the research question to produce analytical themes. The analytical themes were discussed by A1 and A6, and new themes were created if necessary and scrutinised again until they were considered to sufficiently describe the descriptive themes.[36]

Patient and public involvement

This study was conducted without patient or public involvement.

For peer review only

RESULTS

Search and selection results

The study aimed to explore HCPs' experiences of job satisfaction when providing PCC. The results of the systematic search and selection process are presented in Figure 1.

Figure 1. The selection of studies presented in a PRISMA flow diagram.[48]

Characteristics of included studies

The characteristics of the 17 included studies are presented in Table 1. Seven studies were conducted in Sweden,[49-55] four in the United Kingdom (UK),[56-59] two in the Netherlands,[60 61] one in Ireland,[62] one in Portugal,[63] one in Austria and Germany,[64] and one in Sweden, Norway, and Australia.[65]

Most of the studies were qualitative (*n* = 13),[49-54 56-58 61 62 64 65] while four[55 59 60 63] used mixed methods. Eight studies[51 53 55-57 61 62 65] used individual interviews, three[60 63 64] used focus groups, and six[49 50 52 54 58 59] combined individual and focus-group interviews, of which two[50 58] also used dyadic interviews.

The total number of participants in the included studies was 459 (range 6–97).[50 53] Thirteen studies[49-56 60-63 65] reported the gender of participants, who were predominantly women (*n* = 351; 90%). The professions represented were RNs and specialist nurses (*n* = 211),[49-53 55 57 58 61 62 64 65] assistant nurses, students, enrolled nurses, caregivers, direct-care workers, frontline staff (*n* = 189),[50 52 56 57 59 60 63 65] physiotherapists (*n* = 23),[50 54 55 65] occupational therapists (*n* = 15),[50 55 65] physicians (*n* = 10),[50] professional support workers and social workers (*n* = 8),[57 61] and 'other', with the profession not being specified (*n* = 1).[50] Nine studies were conducted in hospitals, [50-54 57 58 62 64] six in elderly-care, residential-care, or nursing homes,[55 56 59 60 63 65] and two in primary-care or GP practices.[49 61]

For peer review only - http://bmjopen.bmj.com/site/about/guidelines.xhtml

Table 1 Characteristics of included studies

Author, year, country	Aim	Data collection method	Analysis method	Context	HCPs
Barbosa *et al*,[63] 2015, Portugal	To explore direct-care workers' experiences of a psychoeducational intervention including person-centred care	Semi-structured focus groups (mixed methods)	Thematic analysis	Four residential care facilities	Direct-care workers (*n* = 50) (8 focus groups) (all women)
Boersma *et al*,[60] 2017, The Netherlands	To explore how caregivers working with a person-centred care method (the Veder method) experience their job satisfaction	Focus groups (mixed methods)	Deductive analysis from the RE-AIM framework	Six nursing home wards	Professional caregivers (*n* = 42) (6 focus groups) (40 women, 2 men)
Boström *et al*,[49] 2014, Sweden	To describe how nurses specialising in diabetes care experience patient-centred care during an intervention for type 2 diabetes	Focus group and individual semi-structured interviews	Qualitative content analysis	Primary health care centres in northern Sweden	Diabetes specialist nurses (*n* = 10) (2 focus groups, 10 individual interviews, same participants) (all women)
Coyne,[62] 2015,	To explore families' and nurses' experiences and	Individual interviews using open-ended questions	Grounded theory	One general hospital ward and one surgical ward for	RNs (*n* = 18) (17 women, 1 man)

Ireland	perceptions of family-centred care			children in a children's hospital, and one surgical ward for children in a general district hospital	
Fridberg *et al*,[50] 2021, Sweden	To describe HCPs' perceptions and experiences of PCC implementation	Focus groups, semi-structured dyadic interviews, and semi-structured individual interviews	Deductive–inductive content analysis	Six healthcare units in a region in central Sweden	97 in total<br>Assistant nurses (*n* = 30), RNs (*n* = 40), physicians (*n* = 10), physiotherapists (*n* = 9), occupational therapists (*n* = 7), 'other' (not specified) (*n* = 1)<br>(65 participants in 15 focus groups, 10 participants in 5 dyadic interviews, 22 individual interviews)<br>(80 women, 17 men)
Kadri *et al*,[56] 2018, UK	To explore home-care staff members' experiences and perceptions of their personhood when working with person-centred care	Semi-structured individual interviews	Secondary qualitative thematic analysis	Six care homes in the UK: three residential care homes and three nursing homes	Care home staff (*n* = 25)<br>(17 women, 8 men)
Karlsson *et al*,[51] 2019, Sweden	To describe RNs' job satisfaction	Semi-structured individual interviews	Systematic text condensation	One university hospital and one county hospital from different healthcare regions in Sweden	RNs (*n* = 25)<br>(23 women, 2 men)
Kirkley *et al*,[59] 2011, UK: England, Scotland	To explore cultural organisational perspectives in the provision of person-centred care	Focus groups, individual interviews (mixed methods)	Qualitative thematic analysis	Organisations in England and Scotland providing respite care	Frontline staff (*n* = 17)

Kjörnsberg *et al*,[53] 2010, Sweden	To investigate RNs' experiences of transitioning to patient-focused care	Open-ended informal individual interviews	Latent qualitative content analysis	A ward for infectious diseases at a university hospital in central Sweden	RNs (n = 6) (all women)
Nilsson *et al*,[52] 2019, Sweden	To explore RNs' experiences of working with person-centred care	Individual semi-structured interviews, focus groups	Qualitative content analysis	An acute ward at a university hospital in Sweden	14 in total RNs, enrolled nurses (12 women, 2 men)
Pinkert *et al*,[64] 2018, Austria and Germany	To describe the experiences of RNs working with dementia patients in acute-care hospital contexts	Semi-structured focus groups	Secondary qualitative content analysis	Acute-care settings in four Austrian hospitals and five German hospitals	RNs (n = 68) RNs from Austrian wards (n = 46) and German wards (n = 22) (12 focus groups)
Ross *et al*,[57] 2015, UK	To describe nurses' perspectives on facilitators of person-centred care	Individual semi-structured interviews	Framework analysis	An acute ward at a UK teaching hospital	14 in total RNs (n = 7), support workers (n = 3), student nurses (n = 4)
Sjöberg and Forsner,[54] 2020, Sweden	To explore physiotherapists' varying perceptions of person-centred care	Focus groups, semi-structured individual interviews	Analysis in phenomenography in accordance with Sjöström and Dahlgren (2002)	Wards for acute medicine, rheumatology, surgery, and orthopaedics	Physiotherapists (n = 7) (7 individual interviews, 1 focus group with 3 participants from individual interviews) (6 women, 1 man)
Uittenbroek *et al*,[61] 2018, The Netherlands	To explore case managers' perceptions of their professional role when working with person-centred care	Individual interviews using topic-based interview guide	Grounded theory	GP practices in northern Netherlands	11 in total RNs (n = 6), social workers (n = 5) (All women)

Vassbø *et al*,[65] 2019, Australia, Norway, and Sweden	To illuminate how HCPs in nursing homes experience the meaning of person-centred care focusing on their job satisfaction	Individual open-ended interviews	Phenomenological–hermeneutical analysis for research into lived experiences	Three nursing homes: one in a city in Norway, one in rural Sweden, and one in rural Australia	29 in total<br>RNs (n = 10), enrolled nurses (n = 12), care assistants (n = 2), personal carers (n = 2), occupational therapists (n = 2), physiotherapist (n = 1) (all women)
Walker and Deacon,[58] 2016, UK	To explore nurses' experiences of person-centred care of the suddenly bereaved	Group interviews, dyadic interview, and individual interview	Qualitative directed content analysis	A hospital with critical and acute-care services	RNs (n = 9)<br>(2 group interviews with 7 participants, 1 dyadic and 1 individual interview)
Öhman *et al*,[55] 2017, Sweden	To explore and improve knowledge of the factors contributing to HCPs' job satisfaction	Individual interviews using thematic interview guide (mixed methods)	Grounded theory, qualitative content analysis	Six elderly-care facilities in Sweden	17 in total<br>RNs (n = 5), physiotherapists (n = 6), occupational therapists (n = 6)<br>(14 women, 3 men)

Synthesis

Twenty-four descriptive themes were derived from the codes, and eight analytical themes from the descriptive themes (Table 2). The analytical themes were generated by going beyond the content of the primary studies, representing interpretations of the included studies as a whole.[36]

Table 2 Themes from the qualitative synthesis

Analytical themes	Descriptive themes	Studies included in themes
HCPs feeling torn and inadequate	Conflict between organisational structures and PCC<br>Conflict between disease-oriented task care and PCC<br>Feelings of inadequacy and guilt	Coyne,[62] Fridberg *et al*,[50] Kadri *et al*,[56] Kjörnsberg *et al*,[53] Pinkert *et al*,[64] Uittenbroek *et al*,[61]
PCC demanding for HCPs	High workloads hamper the work with PCC<br>PCC not affecting or is negative for workload<br>Stressful due to lack of resources	Boersma *et al*,[60] Boström *et al*,[49] Coyne,[62] Fridberg *et al*,[50] Kadri *et al*,[56] Karlsson *et al*,[51] Kjörnsberg *et al*,[53] Ross *et al*,[57] Sjöberg and Forsner,[54] Uittenbroek *et al*,[61]
Remoulded professional role	Disorientation and uncertainties with new approach and routines<br>Loss of control<br>Patients having considerable influence	Boström *et al*,[49] Fridberg *et al*,[50] Kadri *et al*,[56] Sjöberg and Forsner[54]

Providing PCC meaningful for HCPs	Improved job satisfaction<br>Meaningfulness – making a difference and doing the 'little extra'<br>In line with ethical expectations<br>Improved relationship between HCPs and patients<br>Self-care for improved care	Barbosa *et al*,[63] Boersma *et al*,[60] Fridberg *et al*,[50] Karlsson *et al*,[51] Kjörnsberg *et al*,[53] Nilsson *et al*, [52] Pinkert *et al*,[64] Ross *et al*,[57] Vassbø *et al*,[65] Öhman *et al*,[55]
HCPs feeling appreciated	Gratitude from patients makes working with PCC easier<br>Feeling valued and appreciated by colleagues	Barbosa *et al*,[63] Boersma *et al*,[60] Nilsson *et al*, [52] Uittenbroek *et al*,[61] Vassbø *et al,*[65] Öhman *et al*[55]
Improved team collaboration	Enhanced collaboration improves satisfaction and working context<br>Social support reduces stress and frustration	Boersma *et al*,[60] Kirkley *et al*,[59] Nilsson *et al*, [52] Uittenbroek *et al*,[61] Vassbø *et al*[65]
Workload and stress reduction	Improves flow and workload<br>Reduced stress at work<br>Sense of increased control and reduced anxiety	Barbosa *et al*,[63] Fridberg *et al*,[50] Kjörnsberg *et al*,[53] Vassbø *et al*[65]
Increased personal motivation and commitment	Feeling joy and energy<br>Thriving at work<br>Willingness and motivation to perform 'ideal'	Barbosa *et al*,[63] Fridberg *et al*,[50] Kadri *et al*,[56] Karlsson *et al*,[51] Vassbø *et al*,[65] Walker and Deacon[58]

For peer review only - http://bmjopen.bmj.com/site/about/guidelines.xhtml

PCC

For peer review only

Analytical themes

HCPs feeling torn and inadequate

Working in line with PCC was experienced as challenging and could make HCPs feel torn and inadequate. The reason for this was described as a conflict between the desire to perform personalised care fulfilling ethical and professional standards, organisational deficiencies in support and negotiation, and an organisational focus on effectiveness.[53 56 61 62 64] A perceived conflict between disease-oriented task care and PCC was described, with HCPs initially uncertain whether, for example, listening attentively was regarded as a task.[61] HCPs who experienced difficulties balancing PCC with the more task-oriented disease care perceived that they were blamed by others, resulting in feelings of guilt and inadequacy.[50 53]

PCC demanding for HCPs

HCPs described how spending time with patients when providing PCC could be demanding and be hampered by high workloads in the organisation.[49 51 54 62] HCPs found that PCC resulted in the same or even a greater workload than before.[50 53 57 61] HCPs also perceived that it could be stressful to work with PCC due to pressures from insufficient resources in terms of time, space, and staffing.[50 56 62] Some HCPs found that PCC did not influence their job satisfaction, especially when dealing with difficult behaviours of patients not responding well to PCC.[60]

Remoulded professional role

HCPs experienced a remoulded professional role when providing PCC. This could result in disorientation and uncertainty due to new structures, routines, and adaptation to new ways of working.[49 54] They perceived their expertise to be compromised and experienced a loss of control, with patients now having more influence over their own care.[54 56] Moreover, HCPs experienced doubt concerning parts of their new role, which was more conversation based than before and felt less valid than the usual, more hands-on approach.[54] Providing PCC could be frustrating with patients who were less communicative and more reserved[54] and in situations in which HCPs did not agree with patients' wishes regarding their care.[50]

Providing PCC meaningful for HCPs

When HCPs could provide PCC in line with ethical expectations and could collaborate with patients, they experienced increased meaningfulness and job satisfaction.[51 52 55 57 64 65] PCC provision was aligned with HCPs' standards and expectations regarding ethics and routines.[50] Spending more time with patients improved the relationship between HCPs and patients.[50 51]

For peer review only

[53 57 60 65] Additionally, HCPs described becoming more aware of the importance of their personal well-being when providing PCC, through its connection to patient well-being. HCPs taking better care of themselves could enable care that both patients and HCPs were content with.[63]

HCPs feeling appreciated

Although some HCPs found that PCC did not influence their job satisfaction when dealing with patients presenting difficult behaviours, others found that providing PCC instead helped in the care of these patients, causing them to show appreciation.[60] Patient expressions of gratification with the care were experienced as leading to increased job satisfaction and enhanced energy.[55 60 65] PCC was also described as a practice leading to appreciation from colleagues, as colleagues could understand and acknowledge one another more through collaboration and interaction, increasing satisfaction and meaningfulness at work.[52 61 63 65]

Improved team collaboration

The enhanced awareness, learning, and collaboration with colleagues and organisations derived from PCC was described as improving job satisfaction and the work environment.[60 61 65] HCPs valued the social support that emerged from working with PCC, fostering a supportive culture in which colleagues shared experiences and feelings, and recognised and relieved each other; this was experienced as increasing job satisfaction and reducing stress and frustration.[52 59 65]

Workload and stress reduction

HCPs who implemented PCC experienced a better workflow with new routines[50] and a calmer work environment.[65] Lower workload and reduced stress were experienced from working with PCC and being responsible for fewer patients.[53] Moreover, HCPs described increased control arising from enhanced independence in organising the work.[65] With PCC, HCPs could better deal with situations formerly experienced as stressful, which was described as reducing anxiety.[63]

Increased personal motivation and commitment

Motivation[56 65] and commitment[51 56 58 63] to continue providing PCC increased. The HCPs experienced enhanced joy from the improved connections with patients[50] and increased energy when they could meet patients' needs.[65] Moreover, HCPs working jointly with PCC, helping one another and discussing the care, generated motivation and an increased striving to improve their current skills.[65]

For peer review only

DISCUSSION

This systematic review identified, assessed, and synthesised qualitative studies to explore HCPs' experiences of providing PCC by focusing on job satisfaction. Seventeen studies published between 2010 and 2021 were synthesised. Most of them were conducted in Sweden, followed by the UK. Most participants in the included studies were women. Participants were mainly RNs or specialist nurses, closely followed by assistant nurses, students, enrolled nurses, care workers, and frontline staff. The themes identified in this review present a pattern of both positive and negative experiences of PCC in relation to job satisfaction.

An interesting finding derived from the synthesis concerned the remoulded professional role. A study by Boström *et al*[66] of RNs' experiences of providing PCC over the phone highlighted that RNs experienced a need to adapt and remould their professional role when working with PCC. Remoulding the professional role was an iterative process in which they had to challenge their thinking and practice, which could generate insecurity;[66] despite being a challenging process, the RNs experienced this remoulding as expanding their professional role.[66] Similarly, in this systematic review, uncertainty towards the new professional role when providing PCC emerged due to the new practice and routines, and an experienced decrease in control and autonomy.[49 50 54 56] Having a system with a PCC culture with well-supported and committed HCPs is a prerequisite for implementing PCC.[67] Moreover, Moore *et al*[26] described how HCPs must adopt a new professional role when working with PCC. HCPs' integration of PCC is a process that requires time for reflecting on and adapting to the theory and practice of PCC for the specific context.[14] A more equal distribution of power, with patients being more involved and seen as experts in their own care, could be experienced as challenging for HCPs used to traditional care. Summer Meranius *et al*[68] argued that PCC could lead to reduced autonomy and negative health impacts for HCPs. To avoid negative health impacts, overcome the challenges of adapting, and instead expand the professional role, HCPs should be provided with time for the process and be encouraged to reflect on the theory and practice of PCC in their specific setting.

HCPs unable to provide the care desired from an ethical standpoint were also apparent. Similar to the finding of Juthberg and Sundin,[69] guilt and inadequacy could arise from being torn between striving to provide PCC, task-oriented care, and organisational structures.[50 53 54]

For peer review only

[56,61,62,64] A review by Güney *et al*[32] showed that the organisation and task-oriented care could be barriers to PCC and that improved support could modify organisational barriers. Ethical stress can also emerge from not being able to provide the care desired due to a lack of resources.[27] Furthermore, perceived stress and job dissatisfaction can increase healthcare turnover rates.[7] However, systematic organisational support when providing PCC can reduce stress of conscience,[70] indicating that systematic organisational support could serve as a preventive measure. Providing resources for HCPs working with PCC could thus potentially reduce stress of conscience and improve job satisfaction; in the long term, this might lead to reduced turnover, with HCPs being more satisfied with the care provided.

HCPs also experienced improved job satisfaction and meaningfulness when providing PCC, being engaged, and spending time with patients,[50-53,55,57,60,64,65] and they described reduced workload and stress from working with PCC.[50,53,65] The mixed experiences found in this review regarding engagement, workload, and stress align with Summer Meranius *et al*,[68] suggesting that engagement in patient care is essential for PCC and enhances the relationship between HCPs and patients; however, too much engagement and commitment can also be considered a risk.[68] The contrasting views of workload and stress may be mediated by organisational support. Whereas reduced stress seemed to be related to resources such as enhanced support, increased stress was related to a lack of resources in term of space, time, and staffing. Structures promoting collaboration with colleagues and more time with patients facilitate a conducive working environment, promoting PCC.[67] This underpins the benefits of supporting HCPs with sufficient resources to provide PCC. Moreover, it aligns with WHO's efforts to promote the co-creation of care in the interest of improved working conditions.[71]

The HCPs felt increased appreciation from colleagues when providing PCC.[52,61,63,65] Additionally, improved team collaboration when providing PCC was described as leading to enhanced satisfaction with the work environment.[52,60,61,65] The findings regarding those themes are well aligned with Montgomery *et al*,[72] who found a positive association between well-functioning teamwork and engagement. This suggests that collaboration in providing PCC could have a role in improving the health and well-being of HCPs.

As in the review by van den Pol-Grevelink,[31] increased thriving, motivation, and commitment were also apparent.[51,56,58,63,65] Keyko *et al*[73] found that HCPs engaged in their work will experience increased motivation and well-being, resulting in higher-quality care. Moreover, healthy HCPs are a prerequisite for the consistent provision of PCC.[74] HCPs increasingly

For peer review only

valued their personal well-being, which meant that they could provide better, more satisfying care.[63] HCPs providing care that helps them thrive and be satisfied could mean healthier HCPs and decreased turnover in healthcare, facilitating the continuous provision of PCC.

Strengths and limitations

A strength of this review was that its authors had different professional backgrounds, with A1, A3, and A4 coming from nursing, A2 from sociology, and A5 and A6 from social medicine. In the selection and research process, pre-understanding was addressed through regular discussions in the group. Moreover, a librarian was consulted, several databases were searched, a broad range of terms was used, and the reference lists of the included studies were scrutinised. Also, two authors independently screened the titles, abstracts, and full texts for inclusion.

A systematic review was a suitable method for the present research purpose, since the aim was to explore and synthesise findings regarding a particular research question.[75] To demonstrate transparency, reduce the risk of bias, and enable replicability, a PRISMA protocol was published in PROSPERO before data collection, a PRISMA flow diagram was used, and data from extracted studies were presented in tables.[48 76-78] It was a strength that the synthesis strictly followed the steps described by Thomas and Harden,[36] since these have been proven viable for thematically synthesising qualitative experiences of healthcare implementations. The themes derived from the qualitative synthesis can be valuable as guidance for further research or can inform policy and practice concerning HCPs' job satisfaction.[75 79]

Some potentially relevant studies might not have been captured since only English-language studies, specific search terms, and studies published in or since 2010 were included. Recent studies are more likely to capture the current healthcare situation.

The search was delimited regarding the countries included in the eligibility criteria. Two studies were included despite being partly conducted in countries not included in the criteria, which could be considered a limitation. Although Vassbø *et al*[65] collected some of the data in Australia, two of the three countries represented in the data collection (i.e., Sweden and Norway) fit the inclusion criteria and the study contained rich data, so the study was included after discussions. The study by Pinkert *et al*[64] was conducted in Germany and Austria, with the latter not being included in the criteria; however, these two countries have similar

For peer review only

healthcare systems and the working conditions in the dementia care and acute settings are similar, so this study was also deemed relevant in order to facilitate a comprehensive overview.[64 80] Additionally, the included studies applied different methodologies and theoretical approaches within qualitative research, which might have affected how the studies were interpreted and compared.

CONCLUSION

This qualitative systematic review identified varying HCP experiences concerning job satisfaction when providing PCC in healthcare settings in Europe. A noteworthy aspect of the synthesis was the experienced new professional role, which came with its own demands and (initially) resulted in, for example, disorientation and uncertainty. Vitally, the new role also came with meaningfulness, an improved relationship between HCPs and patients, appreciation from patients and colleagues, and enhanced team collaboration. An implication of this is the importance of healthcare organisations focusing on supporting HCPs in their new professional role in providing PCC.

Author contributions

All authors developed and contributed to the review in collaboration. A1 developed the search strategy, which was discussed in meetings with all authors. A1, A3, and A6 developed the protocol. A1 and A2, supported by A3, screened studies for inclusion. A1, supported by A6, extracted, and synthesised the data, which were then discussed and reviewed by all authors. A first draft of the manuscript was written by A1, with feedback from A2 and A4. The manuscript was then revised several times by A1 after feedback from A3, A5, and A6. The final version of the manuscript was approved by all authors.

Acknowledgements

We wish to thank N.N. at the Biomedical Library for contributing to the search strategy.

Funding

This work was supported by grants from the AFA Insurance (no.190030), an organisation owned by Sweden's labour market parties, and the University of Gothenburg Centre for Person-Centred Care (GPCC), Sweden. GPCC is funded by the Swedish Government's grant for Strategic Research Areas, Care Sciences (Application to Swedish Research Council no.

For peer review only

2009–1088). The funders have no role in the design of the study, data collection, analysis or interpretation.

Competing interests

None declared.

Patient consent for publication

Not required.

Ethics approval

No formal ethics approval was needed since this review is based on publicly available data from previously published studies.

Provenance and peer review

Not commissioned; externally peer reviewed.

Data availability statement

Data sharing not applicable as no datasets were generated and/or analysed for this study. This is a systematic review, and all the data used are taken from previously published material.

Supplemental material

For peer review only

REFERENCES

1. The European Agency for Safety and Health at Work. Third European Survey of Enterprises on New and Emerging Risks (ESENER 3) https://osha.europa.eu/en/publications/third-european-survey-enterprises-new-and-emerging-risks-esener-3/view, 2019.

2. The National Institute for Occupational Safety and Health. Healthcare Workers: Work Stress & Mental Health 2016 [Available from: https://www.cdc.gov/niosh/topics/healthcare/workstress.html accessed 21 February, 2022.

3. Harrison DA, Newman DA, Roth PL. How Important Are Job Attitudes? Meta-Analytic Comparisons of Integrative Behavioral Outcomes and Time Sequences. *Academy of Management journal* 2006;49(2):305-25. doi: 10.5465/AMJ.2006.20786077

4. Broetje S, Jenny GJ, Bauer GF. The Key Job Demands and Resources of Nursing Staff: An Integrative Review of Reviews. *Frontiers in Psychology* 2020;11 doi: 10.3389/fpsyg.2020.00084

5. Bigliardi B, Ivo Dormio A, Galati F, et al. The impact of organizational culture on the job satisfaction of knowledge workers. *VINE Very informal newsletter on library automation* 2012;42(1):36-51. doi: 10.1108/03055721211207752

6. Portoghese I, Galletta M, Coppola RC, et al. Burnout and workload among health care workers: the moderating role of job control. *Saf Health Work* 2014;5(3):152-57. doi: 10.1016/j.shaw.2014.05.004 [published Online First: 2014/06/07]

7. Halter M, Boiko O, Pelone F, et al. The determinants and consequences of adult nursing staff turnover: a systematic review of systematic reviews. *BMC Health Services Research* 2017;17(1):824. doi: 10.1186/s12913-017-2707-0

8. WHO. Spending on health in Europe: entering a new era. https://apps.who.int/iris/bitstream/handle/10665/340910/9789289055079-eng.pdf, 2021.

9. The European Commission. Recruitment and Retention of the Health Workforce in Europe. https://ec.europa.eu/health/other-pages/basic-page/recruitment-and-retention-health-workforce-europe-2015_en, 2015.

10. The European Commission. Occupational health and safety risks in the healthcare sector. https://op.europa.eu/en/publication-detail/-/publication/b29abb0a-f41e-4cb4-b787-4538ac5f0238/language-en/format-PDF/source-251761322, 2010.

11. Swedish Institute for Standards. Patient involvement in health care - Minimum requirements for person-centred care. https://www.sis.se/en/produkter/health-care-technology/general/health-care-services-in-general/ss-en-173982020/, 2020.

12. Ekman I. The GPCC. *European Heart Journal* 2018;39(7):495-96. doi: 10.1093/eurheartj/ehy029

For peer review only

13. Ekman I. Practising the ethics of person-centred care balancing ethical conviction and moral obligations. *Nurs Philos* 2022;23(3):e12382. doi: 10.1111/nup.12382 [published Online First: 20220225]

14. Britten N, Moore L, Lydahl D, et al. Elaboration of the Gothenburg model of person-centred care. *Health Expectations* 2017;20(3):407-18. doi: https://doi.org/10.1111/hex.12468

15. Ekman I, Swedberg K, Taft C, et al. Person-Centered Care — Ready for Prime Time. *European Journal of Cardiovascular Nursing* 2011;10(4):248-51. doi: 10.1016/j.ejcnurse.2011.06.008

16. Ekman N, Moons P, Taft C, et al. Observable indicators of person-centred care: an interview study with patients, relatives and professionals. *BMJ Open* 2022;12(4):e059308. doi: 10.1136/bmjopen-2021-059308

17. Epstein RM, Fiscella K, Lesser CS, et al. Why The Nation Needs A Policy Push On Patient-Centered Health Care. *Health Affairs* 2010;29(8):1489-95.

18. Vennedey V, Hower KI, Hillen H, et al. Patients' perspectives of facilitators and barriers to patient-centred care: insights from qualitative patient interviews. *BMJ Open* 2020;10(5):e033449. doi: 10.1136/bmjopen-2019-033449

19. Chenoweth L, Jeon Y-H, Stein-Parbury J, et al. PerCEN trial participant perspectives on the implementation and outcomes of person-centered dementia care and environments. *International Psychogeriatrics* 2015;27(12):2045-57. doi: 10.1017/S1041610215001350 [published Online First: 2015/08/26]

20. Mead N, Bower P. Patient-centredness: a conceptual framework and review of the empirical literature. *Soc Sci Med* 2000;51(7):1087-110. doi: 10.1016/s0277-9536(00)00098-8

21. Epstein RM. The science of patient-centered care. *J Fam Pract* 2000;49(9):805-7.

22. Beach MC, Inui T, Relationship-Centered Care Research N. Relationship-centered care. A constructive reframing. *J Gen Intern Med* 2006;21 Suppl 1(Suppl 1):S3-S8. doi: 10.1111/j.1525-1497.2006.00302.x

23. Kuo DZ, Houtrow AJ, Arango P, et al. Family-centered care: current applications and future directions in pediatric health care. *Matern Child Health J* 2012;16(2):297-305. doi: 10.1007/s10995-011-0751-7

24. Weiner SJ. Contextualizing medical decisions to individualize care: lessons from the qualitative sciences. *J Gen Intern Med* 2004;19(3):281-85. doi: 10.1111/j.1525-1497.2004.30261.x

25. Hughes JC, Bamford C, May C. Types of centredness in health care: themes and concepts. *Med Health Care Philos* 2008;11(4):455-63. doi: 10.1007/s11019-008-9131-5 [published Online First: 20080409]

26. Moore L, Britten N, Lydahl D, et al. Barriers and facilitators to the implementation of person-centred care in different healthcare contexts. *Scand J Caring Sci* 2017;31(4):662-73. doi: 10.1111/scs.12376 [published Online First: 2016/11/08]

27. Aiken LH, Sloane DM, Bruyneel L, et al. Nurses' reports of working conditions and hospital quality of care in 12 countries in Europe. *International Journal of Nursing Studies* 2013;50(2):143-53. doi: https://doi.org/10.1016/j.ijnurstu.2012.11.009

For peer review only

28. van Diepen C, Fors A, Ekman I, et al. Associations between person-centred care and job strain, stress of conscience, and intent to leave among hospital personnel. *Journal of Clinical Nursing* 2022;31(5-6):612-22. doi: https://doi.org/10.1111/jocn.15919

29. Britten N, Ekman I, Naldemirci Ö, et al. Learning from Gothenburg model of person centred healthcare. *BMJ* 2020;370:m2738. doi: 10.1136/bmj.m2738

30. Rajamohan S, Porock D, Chang Y-P. Understanding the Relationship Between Staff and Job Satisfaction, Stress, Turnover, and Staff Outcomes in the Person-Centered Care Nursing Home Arena. *Journal of Nursing Scholarship* 2019;51(5):560-68. doi: https://doi.org/10.1111/jnu.12488

31. van den Pol-Grevelink A, Jukema JS, Smits CHM. Person-centred care and job satisfaction of caregivers in nursing homes: a systematic review of the impact of different forms of person-centred care on various dimensions of job satisfaction. *International Journal of Geriatric Psychiatry* 2012;27(3):219-29. doi: https://doi.org/10.1002/gps.2719

32. Güney S, Karadağ A, El-Masri M. Perceptions and experiences of person-centered care among nurses and nurse aides in long term residential care facilities: A systematic review of qualitative studies. *Geriatric Nursing* 2021;42(4):816-24. doi: https://doi.org/10.1016/j.gerinurse.2021.04.005

33. van Diepen C, Fors A, Ekman I, et al. Association between person-centred care and healthcare providers' job satisfaction and work-related health: a scoping review. *BMJ Open* 2020;10(12):e042658. doi: 10.1136/bmjopen-2020-042658 [published Online First: 2020/12/10]

34. Barbosa A, Sousa L, Nolan M, et al. Effects of Person-Centered Care Approaches to Dementia Care on Staff: A Systematic Review. *Am J Alzheimers Dis Other Demen* 2015;30(8):713-22. doi: 10.1177/1533317513520213 [published Online First: 2014/01/23]

35. Shamseer L, Moher D, Clarke M, et al. Preferred reporting items for systematic review and meta-analysis protocols (PRISMA-P) 2015: elaboration and explanation. *BMJ : British Medical Journal* 2015;349:g7647. doi: 10.1136/bmj.g7647

36. Thomas J, Harden A. Methods for the thematic synthesis of qualitative research in systematic reviews. *BMC Medical Research Methodology* 2008;8(1):45. doi: 10.1186/1471-2288-8-45

37. Tong A, Flemming K, McInnes E, et al. Enhancing transparency in reporting the synthesis of qualitative research: ENTREQ. *BMC Medical Research Methodology* 2012;12(1):181. doi: 10.1186/1471-2288-12-181

38. Bettany-Saltikov J. How to do a systematic literature review in nursing : a step-by-step guide. 2. ed. ed: London : McGraw-Hill Education/Open University Press 2016.

39. Rosengren K, Brannefors P, Carlström E. Adoption of the concept of person-centred care into discourse in Europe: a systematic literature review. *Journal Of Health Organization And Management, 2021, Vol 35, Iss 9, pp 265-280* 2021;35(9):265-80. doi: 10.1108/JHOM-01-2021-0008

40. National Library of Medicine. Medical Subject Headings 2022 [Available from: https://www.nlm.nih.gov/mesh/meshhome.html accessed 11 February, 2022.

41. Sayers A. Tips and tricks in performing a systematic review--chapter 4. *Br J Gen Pract* 2008;58(547):136-36. doi: 10.3399/bjgp08X277168

For peer review only - http://bmjopen.bmj.com/site/about/guidelines.xhtml

42. Bramer WM, Giustini D, de Jonge GB, et al. De-duplication of database search results for systematic reviews in EndNote. *J Med Libr Assoc* 2016;104(3):240-43. doi: 10.3163/1536-5050.104.3.014

43. Ouzzani M, Hammady H, Fedorowicz Z, et al. Rayyan-a web and mobile app for systematic reviews. *Syst Rev* 2016;5(1):210. doi: 10.1186/s13643-016-0384-4 [published Online First: 2016/12/07]

44. Polanin JR, Pigott TD, Espelage DL, et al. Best practice guidelines for abstract screening large-evidence systematic reviews and meta-analyses. *Research Synthesis Methods* 2019;10(3):330-42. doi: https://doi.org/10.1002/jrsm.1354

45. SBU. Appraisal of Studies with Qualitative Methodology. https://www.sbu.se/globalassets/ebm/bedomning_studier_kvalitativ_metodik.pdf, 2020.

46. SBU. Guidance for Assessment of Studies with Qualitative Methodology. https://www.sbu.se/globalassets/ebm/vagledning_granskning_studier_kvalitativ_metodik.pdf, 2020.

47. SBU. SBU:s metodbok. https://www.sbu.se/sv/metod/sbus-metodbok/?pub=48286#48490, 2020.

48. Page MJ, McKenzie JE, Bossuyt PM, et al. The PRISMA 2020 statement: an updated guideline for reporting systematic reviews. *BMJ* 2021;372:n71. doi: 10.1136/bmj.n71

49. Boström E, Isaksson U, Lundman B, et al. Patient-centred care in type 2 diabetes - an altered professional role for diabetes specialist nurses. *Scand J Caring Sci* 2014;28(4):675-82. doi: 10.1111/scs.12092

50. Fridberg H, Wallin L, Tistad M. The innovation characteristics of person-centred care as perceived by healthcare professionals: an interview study employing a deductive-inductive content analysis guided by the consolidated framework for implementation research. *BMC Health Services Research* 2021;21(1):904. doi: 10.1186/s12913-021-06942-y

51. Karlsson AC, Gunningberg L, Bäckström J, et al. Registered nurses' perspectives of work satisfaction, patient safety and intention to stay - A double-edged sword. *J Nurs Manag* 2019;27(7):1359-65. doi: 10.1111/jonm.12816

52. Nilsson A, Edvardsson D, Rushton C. Nurses' descriptions of person-centred care for older people in an acute medical ward-On the individual, team and organisational levels'. *J Clin Nurs* 2019;28(7):1251-59. doi: https://doi.org/10.1111/jocn.14738

53. Kjörnsberg A, Karlsson L, Babra A, et al. Registered nurses' opinions about patient focused care. *Australian Journal of Advanced Nursing* 2010;28(1):35-44.

54. Sjöberg V, Forsner M. Shifting roles: physiotherapists' perception of person-centered care during a pre-implementation phase in the acute hospital setting - A phenomenographic study. *Physiother Theory Pract* 2020:1-11. doi: 10.1080/09593985.2020.1809042

55. Öhman A, Keisu BI, Enberg B. Team social cohesion, professionalism, and patient-centeredness: Gendered care work, with special reference to elderly care - a mixed methods study. *BMC Health Serv Res* 2017;17(1):381. doi: https://doi.org/10.1186/s12913-017-2326-9

For peer review only

56. Kadri A, Rapaport P, Livingston G, et al. Care workers, the unacknowledged persons in person-centred care: A secondary qualitative analysis of UK care home staff interviews. *PLoS One* 2018;13(7):e0200031. doi: 10.1371/journal.pone.0200031

57. Ross H, Tod AM, Clarke A. Understanding and achieving person-centred care: the nurse perspective. *J Clin Nurs* 2015;24(9):1223-33. doi: 10.1111/jocn.12662

58. Walker W, Deacon K. Nurses' experiences of caring for the suddenly bereaved in adult acute and critical care settings, and the provision of person-centred care: A qualitative study. *Intensive Crit Care Nurs* 2016;33:39-47. doi: 10.1016/j.iccn.2015.12.005

59. Kirkley C, Bamford C, Poole M, et al. The impact of organisational culture on the delivery of person-centred care in services providing respite care and short breaks for people with dementia. *Health Soc Care Community* 2011;19(4):438-48. doi: 10.1111/j.1365-2524.2011.00998.x

60. Boersma P, Dröes RM, Lissenberg-Witte BI, et al. Does working with the Veder Contact Method influence the job satisfaction of caregivers? A non-randomized controlled trial in nursing homes for people with dementia. *Int Psychogeriatr* 2017;29(12):2017-32. doi: 10.1017/S1041610217001582

61. Uittenbroek RJ, van der Mei SF, Slotman K, et al. Experiences of case managers in providing person-centered and integrated care based on the Chronic Care Model: A qualitative study on embrace. *PLoS One* 2018;13(11):e0207109. doi: 10.1371/journal.pone.0207109

62. Coyne I. Families and health-care professionals' perspectives and expectations of family-centred care: hidden expectations and unclear roles. *Health Expect* 2015;18(5):796-808. doi: 10.1111/hex.12104

63. Barbosa A, Nolan M, Sousa L, et al. Supporting direct care workers in dementia care: effects of a psychoeducational intervention. *Am J Alzheimers Dis Other Demen* 2015;30(2):130-8. doi: https://doi.org/10.1177/1533317514550331

64. Pinkert C, Faul E, Saxer S, et al. Experiences of nurses with the care of patients with dementia in acute hospitals: A secondary analysis. *J Clin Nurs* 2018;27(1):162-72. doi: 10.1111/jocn.13864

65. Vassbø TK, Kirkevold M, Edvardsson D, et al. The meaning of working in a person-centred way in nursing homes: a phenomenological-hermeneutical study. *BMC Nurs* 2019;18:45. doi: https://doi.org/10.1186/s12912-019-0372-9

66. Boström E, Ali L, Fors A, et al. Registered nurses' experiences of communication with patients when practising person-centred care over the phone: a qualitative interview study. *BMC Nurs* 2020;19:54. doi: https://doi.org/10.1186/s12912-020-00448-4

67. Santana MJ, Manalili K, Jolley RJ, et al. How to practice person-centred care: A conceptual framework. *Health Expectations* 2018;21(2):429-40. doi: https://doi.org/10.1111/hex.12640

68. Summer Meranius M, Holmström IK, Håkansson J, et al. Paradoxes of person-centred care: A discussion paper. *Nursing Open* 2020;7(5):1321-29. doi: https://doi.org/10.1002/nop2.520

69. Juthberg C, Sundin K. Registered nurses' and nurse assistants' lived experience of troubled conscience in their work in elderly care--a phenomenological hermeneutic study. *Int J Nurs Stud* 2010;47(1):20-9. doi: 10.1016/j.ijnurstu.2009.06.001 [published Online First: 20090701]

For peer review only

70. Backman A, Sjögren K, Lövheim H, et al. The influence of nursing home managers' leadership on person-centred care and stress of conscience: A cross-sectional study. *BMC Nursing* 2021;20(1):200. doi: 10.1186/s12912-021-00718-9

71. WHO. WHO global strategy on integrated people-centred health services 2016-2026. https://interprofessional.global/wp-content/uploads/2019/11/WHO-2015-Global-strategy-on-integrated-people-centred-health-services-2016-2026.pdf, 2016.

72. Montgomery A, Spânu F, Băban A, et al. Job demands, burnout, and engagement among nurses: A multi-level analysis of ORCAB data investigating the moderating effect of teamwork. *Burnout Research* 2015;2(2):71-79. doi: https://doi.org/10.1016/j.burn.2015.06.001

73. Keyko K, Cummings GG, Yonge O, et al. Work engagement in professional nursing practice: A systematic review. *Int J Nurs Stud* 2016;61:142-64. doi: 10.1016/j.ijnurstu.2016.06.003 [published Online First: 2016/06/29]

74. Hower KI, Vennedey V, Hillen HA, et al. Implementation of patient-centred care: which organisational determinants matter from decision maker's perspective? Results from a qualitative interview study across various health and social care organisations. *BMJ Open* 2019;9(4):e027591. doi: 10.1136/bmjopen-2018-027591

75. Snyder H. Literature review as a research methodology: An overview and guidelines. *Journal of Business Research* 2019;104:333-39. doi: https://doi.org/10.1016/j.jbusres.2019.07.039

76. Booth A, Clarke M, Ghersi D, et al. An international registry of systematic-review protocols. *The Lancet* 2011;377(9760):108-09. doi: https://doi.org/10.1016/S0140-6736(10)60903-8

77. Xiao Y, Watson M. Guidance on Conducting a Systematic Literature Review. *Journal of Planning Education and Research* 2017;39(1):93-112. doi: 10.1177/0739456X17723971

78. Butler A, Hall H, Copnell B. A Guide to Writing a Qualitative Systematic Review Protocol to Enhance Evidence-Based Practice in Nursing and Health Care. *Worldviews on Evidence-Based Nursing* 2016;13(3):241-49. doi: https://doi.org/10.1111/wvn.12134

79. Noyes J, Booth A, Cargo M, et al. Qualitative evidence. Cochrane Handbook for Systematic Reviews of Interventions2019:525-45.

80. The European Parliament. Health Care Systems in the EU: A Comparative Study. https://www.europarl.europa.eu/workingpapers/saco/pdf/101_en.pdf, 1998.

For peer review only

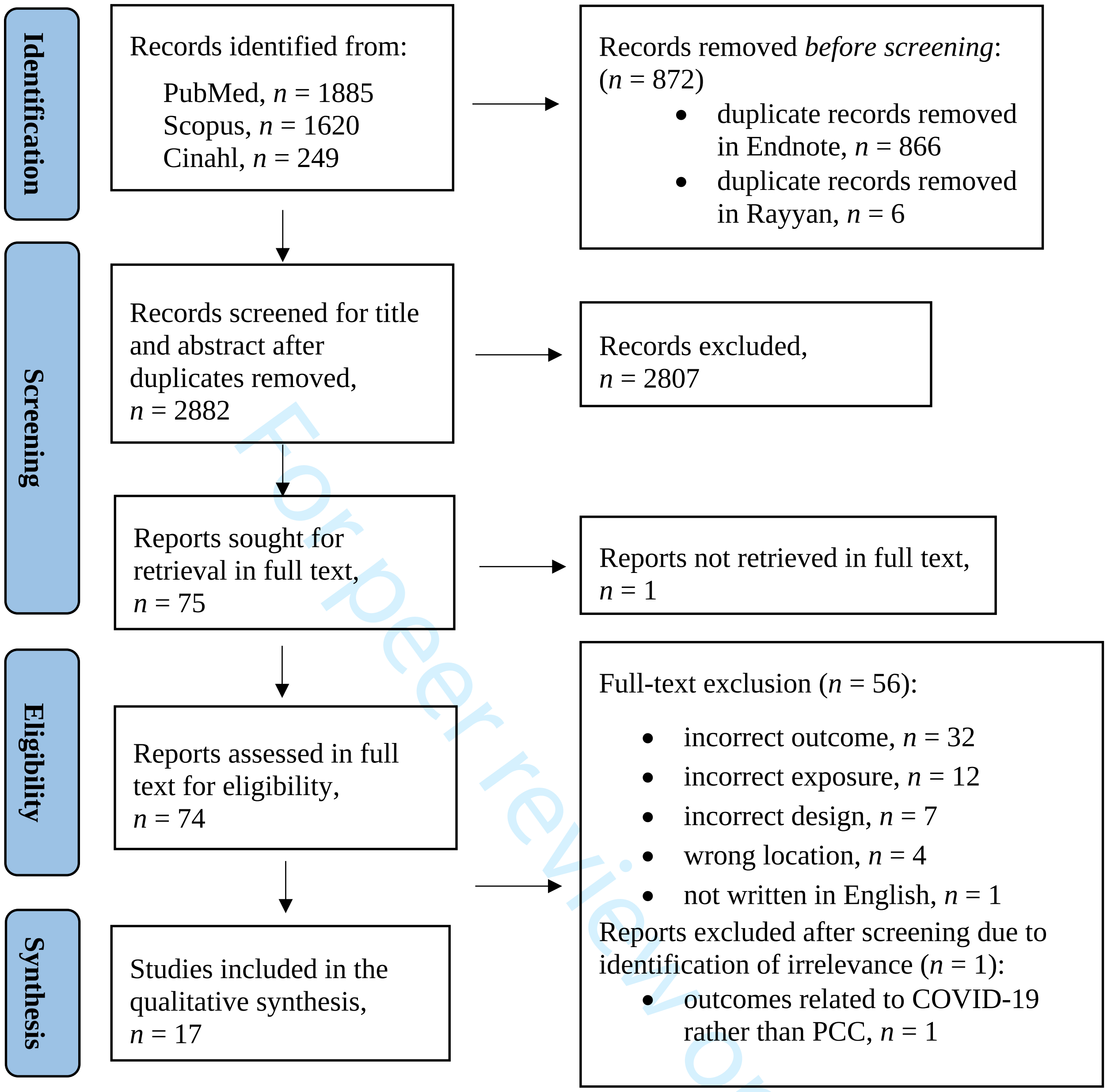

For peer review only

NIHR | National Institute for Health Research

PROSPERO
International prospective register of systematic reviews

To enable PROSPERO to focus on COVID-19 submissions, this registration record has undergone basic automated checks for eligibility and is published exactly as submitted. PROSPERO has never provided peer review, and usual checking by the PROSPERO team does not endorse content. Therefore, automatically published records should be treated as any other PROSPERO registration. Further detail is provided here.

Citation

Kristoffer Gustavsson, Andreas Fors, Cornelia van Diepen. Healthcare providers' experiences of job satisfaction and work-related health when providing Person-Centred Care: a systematic review of qualitative studies in Europe. PROSPERO 2022 CRD42022304732 Available from:
https://www.crd.york.ac.uk/prospero/display_record.php?ID=CRD42022304732

For peer review only

Review question

How does healthcare providers' experience their job satisfaction and work-related health when providing Person-Centred Care?

Searches

The databases that was searched were: CINAHL, PubMed and Scopus. The search was conducted on December 21, 2021. A re-run of the search will be conducted before the analysis to ensure that no relevant articles will be excluded.

Types of study to be included

The review will include studies based on criteria such as:

- Study designs with a qualitative approach, which also includes the qualitative component from mixed methods studies.

- Peer-reviewed studies conducted with an ethical approval.

- Studies from 2010 and forward.

- An English, full-text document of the study exists.

Condition or domain being studied

Healthcare provider outcomes will be studied, e.g., job satisfaction, job strain, stress, stress of conscience, burnout, psychosocial work environment and intent to leave.

Participants/population

Inclusion:

Healthcare providers that have worked or are working with Person-Centred Care.

Intervention(s), exposure(s)

What will be of interest from the included studies are healthcare providers' experiences of their job satisfaction and work-related health when providing Person-Centred Care.

Comparator(s)/control

Not applicable.

Context

Studies from all healthcare settings, and research from countries in Europe with a healthcare model that can be discerned are eligible for inclusion.

Main outcome(s)

The review aims to further explore healthcare providers' experiences of their job satisfaction and work-related health when providing Person-Centred Care. This could for example include outcomes such as: job strain, stress, stress of conscience and job satisfaction.

Measures of effect

Not applicable.

Additional outcome(s)

Not applicable.

Measures of effect

Not applicable.

Data extraction (selection and coding)

Study selection:

The search was exported into endnote to remove duplicates, and then exported into Rayyan for screening. Two research group members will independently and blinded screen for title/abstract and then conduct full-text screening. Articles of uncertainty will also be noted and scrutinised. If there is a disagreement on whether to include an article, this will be solved by discussion, and if still not resolved, another research group member will be consulted. Full-text articles meeting the inclusion criteria will be exported into Nvivo for data coding.

The process of including articles will be presented in a PRISMA flow chart.

Examples of data that will be extracted: Author names, journal, year/country of publication, research aim/question, study design, sampling method, sample size, participant demographics, data collection method, analysis-method.

Risk of bias (quality) assessment

The Swedish Agency for Health Technology Assessment and Assessment for Social Services (SBU) checklist will be used to evaluate the study's methodology, and the instrument also serves to prepare for the GRADE-CERQual assessment (SBU, 2020).

To evaluate the qualitative evidence and assess how much confidence to put in the qualitative findings from the synthesis of studies, the GRADE-CERQual will be used (Lewin, 2015). The GRADE-CERQual checklist covers questions related to the study and includes four "risk areas" that will be assessed through these questions. The four areas are: Methodological limitations, Relevance, Coherence, and Adequacy of data.

The critical appraisal conducted with the SBU checklist and the GRADE-CERQual will be filled out independently by two reviewers from the research group.

References:

Lewin, S., Glenton, C., Munthe-Kaas, H., Carlsen, B., Colvin, C., Gülmezoglu, M., & Rashidian, A. (2015). Using Qualitative Evidence in Decision Making for Health and Social Interventions: An Approach to Assess Confidence in Findings from Qualitative Evidence Syntheses (GRADE-CERQual). PLoS Medicine, 12(10), E1001895.
Swedish Agency for Health Technology Assessment and Assessment for Social Services [SBU]. (2020). Bedömning av studier med kvalitativ metodik. Retrieved 2022-01-10 from https://www.sbu.se/globalassets/ebm/bedomning_studier_kvalitativ_metodik.pdf

Strategy for data synthesis

This systematic review will use thematic synthesis, following the steps from Thomas & Harden (2008). The thematic synthesis will be conducted in three stages:

Stage one:

This stage serves to become familiar with the text at hand by reading it line-by-line and producing initial

For peer review only

codes.

Stage two:

In this stage, descriptive themes are produced, deriving from the individual codings. They can also be modified with further coding levels during this process if deemed necessary.

Stage three:

In the last stage, analytical themes will be generated from the descriptive themes to "go beyond" the initial studies' content by being analysed in relation to the purpose and aim of the review.

During the whole analysis process, disagreements will be solved through discussions, and if needed, through consultations with other research group members to reach a consensus.

References

Thomas, J., & Harden, A. (2008). Methods for the thematic synthesis of qualitative research in systematic reviews. BMC Medical Research Methodology, 8(1), 45.

Analysis of subgroups or subsets

Not applicable.

Contact details for further information

Mr Kristoffer Gustavsson
kristoffer.gustavsson@gu.se

Organisational affiliation of the review

Centre for Person-Centred Care (GPCC), Institute of Health and Care Sciences, Sahlgrenska Academy, University of Gothenburg, Gothenburg, Sweden.
https://www.gu.se/en

Review team members and their organisational affiliations

Mr Kristoffer Gustavsson. Centre for Person-Centred Care (GPCC), Institute of Health and Care Sciences, Sahlgrenska Academy, University of Gothenburg, Gothenburg, Sweden
Assistant/Associate Professor Andreas Fors. Centre for Person-Centred Care (GPCC), Institute of Health and Care Sciences, Sahlgrenska Academy, University of Gothenburg, Gothenburg, Sweden
Assistant/Associate Professor Cornelia van Diepen. Centre for Person-Centred Care (GPCC), Institute of Health and Care Sciences, Sahlgrenska Academy, University of Gothenburg, Gothenburg, Sweden. Erasmus School of Health Policy & Management, Erasmus University Rotterdam, Rotterdam, Netherlands

Collaborators

Professor Gunnel Hensing. School of Public Health and Community Medicine, Institute of Medicine, Sahlgrenska Academy, University of Gothenburg, Sweden
Professor Malin Axelsson. Department of Care Science, Faculty of Health and Society, Malmö University, Malmö, Sweden
Dr Monica Bertilsson. School of Public Health and Community Medicine, Institute of Medicine, Sahlgrenska Academy, University of Gothenburg, Gothenburg, Sweden
Professor Inger Ekman. Centre for Person-Centred Care (GPCC), Institute of Health and Care Sciences, Sahlgrenska Academy, University of Gothenburg, Gothenburg, Sweden

Type and method of review

Synthesis of qualitative studies, Systematic review

Anticipated or actual start date

17 January 2022

Anticipated completion date

01 December 2022

For peer review only

Funding sources/sponsors
No funding or sponsors.

Conflicts of interest

Language
English

Country
Sweden

Stage of review
Review Ongoing

Subject index terms status
Subject indexing assigned by CRD

Subject index terms
MeSH headings have not been applied to this record

Date of registration in PROSPERO
01 March 2022

Date of first submission
29 January 2022

Stage of review at time of this submission

Stage	Started	Completed
Preliminary searches	Yes	Yes
Piloting of the study selection process	No	No
Formal screening of search results against eligibility criteria	No	No
Data extraction	No	No
Risk of bias (quality) assessment	No	No
Data analysis	No	No

The record owner confirms that the information they have supplied for this submission is accurate and complete and they understand that deliberate provision of inaccurate information or omission of data may be construed as scientific misconduct.
The record owner confirms that they will update the status of the review when it is completed and will add publication details in due course.

Versions
01 March 2022
01 March 2022

For peer review only

PROSPERO
International prospective register of systematic reviews

NHS
National Institute for Health Research

UNIVERSITY of York
Centre for Reviews and Dissemination

??For those soon to register with PROSPERO: records may not be submitted between the 23rd Dec - 3rd Jan whilst staff are away and the university is closed for the Christmas period. During this time you may still work on your record and save it for submission in the new year. ??

Systematic review

Fields that have an ***asterisk (*)*** *next to them means that they* ***must be answered. Word limits*** *are provided for each section. You will be unable to submit the form if the word limits are exceeded for any section. Registrant means the person filling out the form*.

For peer review only

2. Original language title.

For reviews in languages other than English, give the title in the original language. This will be displayed with the English language title.

3. * Anticipated or actual start date.

Give the date the systematic review started or is expected to start.

17/01/2022

4. * Anticipated completion date.

Give the date by which the review is expected to be completed.

01/12/2022

5. * Stage of review at time of this submission. [1 change]

This field uses answers to initial screening questions. It cannot be edited until after registration.

Tick the boxes to show which review tasks have been started and which have been completed.

Update this field each time any amendments are made to a published record.

PROSPERO
International prospective register of systematic reviews

NHS National Institute for Health Research

The review has not yet started: No

**Review stage**	**Started**	**Completed**
Preliminary searches	Yes	Yes
Piloting of the study selection process	Yes	Yes
Formal screening of search results against eligibility criteria	Yes	Yes
Data extraction	Yes	Yes
Risk of bias (quality) assessment	Yes	Yes
Data analysis	Yes	Yes

Provide any other relevant information about the stage of the review here.

7. * Named contact email.

Give the electronic email address of the named contact.

9. Named contact phone number.

Give the telephone number for the named contact, including international dialling code.

11. * Review team members and their organisational affiliations.

Give the personal details and the organisational affiliations of each member of the review team. Affiliation refers to groups or organisations to which review team members belong. **NOTE: email and country now MUST be entered for each person, unless you are amending a published record.**

Mr Kristoffer Gustavsson. Centre for Person-Centred Care (GPCC), Institute of Health and Care Sciences, Sahlgrenska Academy, University of Gothenburg, Gothenburg, Sweden
Assistant/Associate Professor Andreas Fors. Centre for Person-Centred Care (GPCC), Institute of Health and Care Sciences, Sahlgrenska Academy, University of Gothenburg, Gothenburg, Sweden
Assistant/Associate Professor Cornelia van Diepen. Centre for Person-Centred Care (GPCC), Institute of Health and Care Sciences, Sahlgrenska Academy, University of Gothenburg, Gothenburg, Sweden. Erasmus School of Health Policy & Management, Erasmus University Rotterdam, Rotterdam, Netherlands

13. * Conflicts of interest.

List actual or perceived conflicts of interest (financial or academic).
None

14. Collaborators.

Give the name and affiliation of any individuals or organisations who are working on the review but who are not listed as review team members. **NOTE: email and country must be completed for each person, unless you are amending a published record.**

Professor Gunnel Hensing. School of Public Health and Community Medicine, Institute of Medicine,

For peer review only

PROSPERO
International prospective register of systematic reviews

National Institute for Health Research

Sahlgrenska Academy, University of Gothenburg, Sweden
Professor Malin Axelsson. Department of Care Science, Faculty of Health and Society, Malmö University, Malmö, Sweden
Dr Monica Bertilsson. School of Public Health and Community Medicine, Institute of Medicine, Sahlgrenska Academy, University of Gothenburg, Gothenburg, Sweden

16. * Searches.

State the sources that will be searched (e.g. Medline). Give the search dates, and any restrictions (e.g. language or publication date). Do NOT enter the full search strategy (it may be provided as a link or attachment below.)

The databases that was searched were: CINAHL, PubMed and Scopus. The search was conducted on December 21, 2021. A re-run of the search will be conducted before the analysis to ensure that no relevant articles will be excluded.

18. * Condition or domain being studied.

Give a short description of the disease, condition or healthcare domain being studied in your systematic review.

Healthcare provider outcomes will be studied, e.g., job satisfaction, job strain, stress, stress of conscience, burnout, psychosocial work environment and intent to leave.

20. * Intervention(s), exposure(s).

Give full and clear descriptions or definitions of the interventions or the exposures to be reviewed. The preferred format includes details of both inclusion and exclusion criteria.

What will be of interest from the included studies are healthcare providers' experiences of their job satisfaction and work-related health when providing Person-Centred Care.

22. * Types of study to be included.

Give details of the study designs (e.g. RCT) that are eligible for inclusion in the review. The preferred format includes both inclusion and exclusion criteria. If there are no restrictions on the types of study, this should be stated.

The review will include studies based on criteria such as:

- Peer-reviewed studies conducted with an ethical approval.

- An English, full-text document of the study exists.

24. * Main outcome(s).

Give the pre-specified main (most important) outcomes of the review, including details of how the outcome is defined and measured and when these measurement are made, if these are part of the review inclusion criteria.

The review aims to further explore healthcare providers' experiences of their job satisfaction and work-related health when providing Person-Centred Care. This could for example include outcomes such as: job strain, stress, stress of conscience and job satisfaction.

Not applicable.

For peer review only - http://bmjopen.bmj.com/site/about/guidelines.xhtml

PROSPERO
International prospective register of systematic reviews

National Institute for Health Research

26. * Data extraction (selection and coding).

Describe how studies will be selected for inclusion. State what data will be extracted or obtained. State how this will be done and recorded.

Study selection:

The process of including articles will be presented in a PRISMA flow chart.

Examples of data that will be extracted: Author names, journal, year/country of publication, research aim/question, study design, sampling method, sample size, participant demographics, data collection method, analysis-method.

28. * Strategy for data synthesis.

Describe the methods you plan to use to synthesise data. This but should be and describe how the proposed approach will be applied to your data. If meta-analysis is planned, describe the models to be used, methods to explore statistical heterogeneity, and software package to be used.

Stage one:

This systematic review will use thematic synthesis, following the steps from Thomas & Harden (2008).

Stage two:

Stage three:

During the whole analysis process, disagreements will be solved through discussions, and if needed, through consultations with other research group members to reach a consensus.

Thomas, J., & Harden, A. (2008). Methods for the thematic synthesis of qualitative research in systematic reviews. BMC Medical Research Methodology, 8(1), 45.

30. * Type and method of review.

Select the type of review, review method and health area from the lists below.

Type of review

Cost effectiveness
No

Diagnostic
No

Epidemiologic
No

Individual patient data (IPD) meta-analysis
No

Intervention
No

Living systematic review

For peer review only

No

Meta-analysis

No

Methodology

No

Narrative synthesis

No

Network meta-analysis

No

Pre-clinical

No

Prevention

No

Prognostic

No

Prospective meta-analysis (PMA)

No

Review of reviews

No

Service delivery

No

Synthesis of qualitative studies

Yes

Systematic review

Yes

Other

No

Health area of the review

Alcohol/substance misuse/abuse

No

Blood and immune system

No

Cancer

No

Cardiovascular

No

For peer review only

Care of the elderly

No

Child health

No

Complementary therapies

No

COVID-19

No

Crime and justice

No

Dental

No

Digestive system

No

Ear, nose and throat

No

Education

No

Endocrine and metabolic disorders

No

Eye disorders

No

General interest

Yes

Genetics

No

Health inequalities/health equity

Yes

Infections and infestations

No

International development

Yes

Mental health and behavioural conditions

Yes

Musculoskeletal

No

For peer review only

PROSPERO
International prospective register of systematic reviews

National Institute for Health Research

Neurological

No

Nursing

No

Obstetrics and gynaecology

No

Oral health

No

Palliative care

No

Perioperative care

No

Physiotherapy

No

Pregnancy and childbirth

No

Public health (including social determinants of health)

No

Rehabilitation

No

Respiratory disorders

No

Service delivery

Yes

Skin disorders

No

Social care

No

Surgery

No

Tropical Medicine

No

Urological

No

Wounds, injuries and accidents

No

Violence and abuse

For peer review only

No

32. * Country.

Select the country in which the review is being carried out. For multi-national collaborations select all the countries involved.

34. Reference and/or URL for published protocol.

If the protocol for this review is published provide details (authors, title and journal details, preferably in Vancouver format)

Add web link to the published protocol.

Or, upload your published protocol here in pdf format. Note that the upload will be publicly accessible.

No I do not make this file publicly available until the review is complete

Please note that the information required in the PROSPERO registration form must be completed in full even if access to a protocol is given.

36. Keywords.

Give words or phrases that best describe the review. Separate keywords with a semicolon or new line. Keywords help PROSPERO users find your review (keywords do not appear in the public record but are included in searches). Be as specific and precise as possible. Avoid acronyms and abbreviations unless these are in wide use.

37. Details of any existing review of the same topic by the same authors.

If you are registering an update of an existing review give details of the earlier versions and include a full bibliographic reference, if available.

38. * Current review status. [1 change]

Update review status when the review is completed and when it is published.New registrations must be ongoing so this field is not editable for initial submission.

Please provide anticipated publication date

Review_Completed_not_published

40. Details of final report/publication(s) or preprints if available.

Leave empty until publication details are available OR you have a link to a preprint (NOTE: this field is not editable for initial submission). List authors, title and journal details preferably in Vancouver format.

For peer review only

PROSPERO
International prospective register of systematic reviews

Give the link to the published review or preprint.

For peer review only

Online Supplementary File 1 – ENTREQ

No	Item	Guide and description	Reported on page #
**1**	Aim	State the research question the synthesis addresses.	2
**2**	Synthesis methodology	Identify the synthesis methodology or theoretical framework which underpins the synthesis, and describe the rationale for choice of methodology *(e.g. meta-ethnography, thematic synthesis, critical interpretive synthesis, grounded theory synthesis, realist synthesis, meta-aggregation, meta-study, framework synthesis).*	5
**3**	Approach to searching	Indicate whether the search was pre-planned (*comprehensive search strategies to seek all available studies)* or iterative (*to seek all available concepts until they theoretical saturation is achieved)*.	3
**4**	Inclusion criteria	Specify the inclusion/exclusion criteria *(e.g. in terms of population, language, year limits, type of publication, study type).*	3
**5**	Data sources	Describe the information sources used (e.g. *electronic databases (MEDLINE, EMBASE, CINAHL, psycINFO, Econlit), grey literature databases (digital thesis, policy reports), relevant organisational websites, experts, information specialists, generic web searches (Google Scholar) hand searching, reference lists)* and when the searches conducted; provide the rationale for using the data sources.	2
**6**	Electronic Search strategy	Describe the literature search *(e.g. provide electronic search strategies with population terms, clinical or health topic terms, experiential or social phenomena related terms, filters for qualitative research, and search limits)*.	2-3
**7**	Study screening methods	Describe the process of study screening and sifting *(e.g. title, abstract and full text review, number of independent reviewers who screened studies).*	4
**8**	Study characteristics	Present the characteristics of the included studies *(e.g. year of publication, country, population, number of participants, data collection, methodology, analysis, research questions).*	6-10
**9**	Study selection results	Identify the number of studies screened and provide reasons for study exclusion *(e,g, for comprehensive searching, provide numbers of studies screened and reasons for exclusion indicated in a figure/flowchart; for iterative searching describe reasons for study exclusion and*	6

For peer review only

No	Item	Guide and description	Reported on page #
		inclusion based on modifications t the research question and/or contribution to theory development).	
**10**	Rationale for appraisal	Describe the rationale and approach used to appraise the included studies or selected findings *(e.g. assessment of conduct (validity and robustness), assessment of reporting (transparency), assessment of content and utility of the findings).*	4
**11**	Appraisal items	State the tools, frameworks and criteria used to appraise the studies or selected findings *(e.g. Existing tools: CASP, QARI, COREQ, Mays and Pope* [25]*; reviewer developed tools; describe the domains assessed: research team, study design, data analysis and interpretations, reporting).*	4
**12**	Appraisal process	Indicate whether the appraisal was conducted independently by more than one reviewer and if consensus was required.	4
**13**	Appraisal results	Present results of the quality assessment and indicate which articles, if any, were weighted/excluded based on the assessment and give the rationale.	4
**14**	Data extraction	Indicate which sections of the primary studies were analysed and how were the data extracted from the primary studies? *(e.g. all text under the headings 'results /conclusions' were extracted electronically and entered into a computer software).*	5
**15**	Software	State the computer software used, if any.	Endnote, NVivo, Rayyan
**16**	Number of reviewers	Identify who was involved in coding and analysis.	5
**17**	Coding	Describe the process for coding of data *(e.g. line by line coding to search for concepts).*	5
**18**	Study comparison	Describe how were comparisons made within and across studies *(e.g. subsequent studies were coded into pre-existing concepts, and new concepts were created when deemed necessary).*	5
**19**	Derivation of themes	Explain whether the process of deriving the themes or constructs was inductive or deductive.	5
**20**	Quotations	Provide quotations from the primary studies to illustrate themes/constructs, and identify whether the quotations were participant quotations of the author's interpretation.	N/A

For peer review only

No	Item	Guide and description	Reported on page #
**21**	Synthesis output	Present rich, compelling and useful results that go beyond a summary of the primary studies (e.g. *new interpretation, models of evidence, conceptual models, analytical framework, development of a new theory or construct).*	11-17, 19

For peer review only

Online Supplementary File 2 – Search strategy

Search strategy				
**Population**	**Exposure**	**Outcome**	**Countries**	**Study design**
"health personnel" "care personnel" "health care provider*" "care provider*" "health care worker*" "health care staff" "caregiver*" "care assistant*" "care aides" "nurse*" "nursing*" "physician*" "physiotherapist*" "physical therapist*" "psychologist*" "social worker*" "health care professional*" "nutritionist*" "case manager*" "medical staff" "general practitioner*"	"person centered" "person centeredness" "client centered" "patient centered" **"patient-centered care"** "relationship centered" "family centered" "patient focused" "person focused" "individualized care" "personalized care" "person-directed care" "individual centered"	**"perception*"** "experience*" "perspective*" **"attitude*"** "stress of conscience" **"job satisfaction" "absenteeism" "presenteeism" "occupational stress" "occupational health" "personnel turnover*" "sick leave" "stress, psychological"** "psychological stress" **"dyssomnia*"** "sleep disorder*" "sleep disturbance*" "moral stress" **"attitude of health personnel"**	"Sweden" "Swedish" "Norway" "Norwegian" "Finland" "Finnish" "Denmark" "Danish" "Iceland" "Icelandic" "United kingdom" "UK" "British" "North Ireland" "North Irish" "Wales" "Welsh" "Scotland" "Scottish" "Ireland" "Irish" "the Netherlands" "Dutch" "Germany" "German" "Spain" "Spanish" "Italy" "Italian" "Belgium" "Belgian" "Portugal" "Portuguese" "Malta" "Maltese" "Estonia" "Estonian" "Slovenia" "Slovenian" "Czech republic" "Czech" "France" "French" "Poland" "polish" "Hungary"	**"qualitative research"** "qualitative stud*" **"focus group*" "grounded theory"** "mixed-method" "ethnography" "phenomenology*" **"interviews as topic"** "interview*" **"hermeneutic*"**

	"Hungarian" "Greece" "Greek" "Latvia" "Latvian" "Serbia" "Serbian"

For peer review only

PubMed 1885

Scopus 1620

Cinahl 249

PubMed searched 21 December 2021

(Sweden OR Swedish OR Norway OR Norwegian OR Finland OR Finnish OR Denmark OR Danish OR Iceland OR Icelandic OR “United Kingdom” OR UK OR British OR North Ireland OR North Irish OR Wales OR Welsh OR Scotland OR Scottish OR Ireland OR Irish OR The Netherlands OR Netherlands OR Dutch OR Germany OR German OR Spain OR Spanish OR Italy OR Italian OR Belgium OR Belgian OR Portugal OR Portuguese OR Malta OR Maltese OR Estonia OR Estonian OR Slovenia OR Slovenian OR Czech Republic OR Czech OR France OR French OR Poland OR Polish OR Hungary OR Hungarian OR Greece OR Greek OR Latvia OR Latvian OR Serbia OR Serbian)

AND

(“health personnel”[MeSH] OR “health personnel”[tiab] OR “care personnel”[tiab] OR “health care provider”[tiab] OR “health care providers”[tiab] OR “care provider”[tiab] OR “care providers”[tiab] OR “health care worker”[tiab] OR “health care staff”[tiab] OR caregiver[tiab] OR caregivers[tiab] OR caregivers[mesh] OR “healthcare worker”[tiab] OR “healthcare workers”[tiab] OR “care assistant”[tiab] OR “care aides”[tiab] OR nurses[mesh] OR nurse[tiab] OR nurses[tiab] OR nursing[tiab] OR physicians[mesh] OR physicians[tiab] OR physician[tiab] OR physiotherapists[tiab] OR “physical therapists”[mesh] OR “physical therapists”[tiab] OR psychologist[tiab] OR “social workers”[mesh] OR “social workers”[tiab] OR “social worker”[tiab] OR “Health care professionals”[tiab] OR “health care professional”[tiab] OR “nutritionist”[tiab] OR nutritionists[mesh] OR “case managers” [tiab] OR “Medical staff”[mesh] OR “Medical staff”[tiab] OR “general practitioners”[tiab] OR “General Practitioners”[mesh])

AND

(“Person centered”[tiab] OR “person centred”[tiab] OR “person-centred”[tiab] OR “person centeredness”[tiab] OR “personcenteredness”[tiab] OR “person centredness”[tiab] OR “client centered”[tiab] OR “client centred”[tiab] OR “patient centered”[tiab] OR “patient centred”[tiab] OR “Patient-Centered Care”[Mesh] OR “relationship centered”[tiab] OR “relationship centred”[tiab] OR “family centred”[tiab] OR “patient focused”[tiab] OR “person

For peer review only

focused"[tiab] OR "individualized care" [tiab] OR "personalized care" [tiab] OR "person-directed care" [tiab] OR "individual centered"[tiab])

AND

(perception[mesh] OR perception[tiab] OR experience[tiab] OR experiences[tiab] OR perspective[tiab] OR attitude[mesh] OR attitude[tiab] OR attitudes[tiab] OR "stress of conscience"[tiab] OR "Job Satisfaction"[Mesh] OR "job satisfaction"[tiab] OR "Absenteeism"[Mesh] OR "absenteeism"[tiab] OR "presenteeism"[Mesh] OR "presenteeism"[tiab] OR "Occupational Stress"[Mesh] OR "occupational stress"[tiab] OR "Personnel Turnover"[Mesh] OR "personnel turnover"[tiab] OR "Sick Leave"[Mesh] OR "sick leave"[tiab] OR "Stress, Psychological"[Mesh] OR "psychological stress"[tiab] OR "Dyssomnias"[Mesh] OR "sleep disorder"[tiab] OR "sleep disorders"[tiab] OR "sleep disturbances"[tiab] OR "occupational health"[Mesh] OR "occupational health"[tiab] OR "moral stress"[tiab] OR "Attitude of Health Personnel"[mesh])

AND

("qualitative research"[mesh] OR "qualitative research"[tiab] OR "qualitative study"[tiab] OR "qualitative studies"[tiab] OR "focus groups"[mesh] OR "focus groups"[tiab] OR "focus group"[tiab] OR "Grounded theory"[mesh] OR "grounded theory"[tiab] OR "mixed-method"[tiab] OR ethnography[tiab] OR phenomenology*[tiab] OR interview*[tiab] OR "interviews as topic"[mesh] OR Hermeneutics[mesh] OR hermeneutic*[tiab])

Scopus searched 21 December 2021

(sweden OR swedish OR norway OR norwegian OR finland OR finnish OR denmark OR danish OR iceland OR icelandic OR "United Kingdom" OR uk OR british OR "North Ireland" OR "North Irish" OR wales OR welsh OR scotland OR scottish OR ireland OR irish OR the AND netherlands OR netherlands OR dutch OR germany OR german OR spain OR spanish OR italy OR italian OR belgium OR belgian OR portugal OR portuguese OR malta OR maltese OR estonia OR estonian OR slovenia OR slovenian OR "Czech Republic" OR czech OR france OR french OR poland OR polish OR hungary OR hungarian OR greece OR greek OR latvia OR latvian OR serbia OR serbian) AND TITLE-ABS-KEY ("health personnel" OR "care personnel" OR "health care provider*" OR "care provider*" OR "health care worker*" OR "health care staff" OR caregiver* OR "healthcare worker*" OR "care assistant*" OR "care

aides" OR nurse* OR nursing OR physician* OR physiotherapist* OR "physical therapist*" OR psychologist* OR "social worker*" OR "Health care professional*" OR "nutritionist*" OR "case manager*" OR "Medical staff" OR "general practitioner*") AND TITLE-ABS-KEY ("Person centered" OR "person centred" OR "person centeredness" OR "personcenteredness" OR "person centredness" OR "client centered" OR "client centred" OR "patient centered" OR "patient centred" OR "Patient-Centered Care" OR "relationship centered" OR "relationship centred" OR "family centred" OR "patient focused" OR "person focused" OR "individualized care" OR "personalized care" OR "person-directed care" OR "individual centered") AND TITLE-ABS-KEY (perception* OR experience* OR perspective* OR attitude* OR "stress of conscience" OR "Job Satisfaction" OR absenteeism OR presenteeism OR "Occupational Stress" OR "Personnel Turnover*" OR "Sick Leave" OR "psychological stress" OR dyssomnia* OR "sleep disorder*" OR "sleep disturbance*" OR "occupational health" OR "moral stress") AND TITLE-ABS-KEY ("qualitative research" OR "qualitative stud*" OR "focus group*" OR "Grounded theory" OR "mixed-method" OR ethnography OR phenomenology OR interview* OR hermeneutic*) AND (LIMIT-TO (PUBYEAR , 2022) OR LIMIT-TO (PUBYEAR , 2021) OR LIMIT-TO (PUBYEAR , 2020) OR LIMIT-TO (PUBYEAR , 2019) OR LIMIT-TO (PUBYEAR , 2018) OR LIMIT-TO (PUBYEAR , 2017) OR LIMIT-TO (PUBYEAR , 2016) OR LIMIT-TO (PUBYEAR , 2015) OR LIMIT-TO (PUBYEAR , 2014) OR LIMIT-TO (PUBYEAR , 2013) OR LIMIT-TO (PUBYEAR , 2012) OR LIMIT-TO (PUBYEAR , 2011) OR LIMIT-TO (PUBYEAR , 2010))

Cinahl searched 21 December 2021

(Sweden OR Swedish OR Norway OR Norwegian OR Finland OR Finnish OR Denmark OR Danish OR Iceland OR Icelandic OR "United Kingdom" OR UK OR British OR "North Ireland" OR "North Irish" OR Wales OR Welsh OR Scotland OR Scottish OR Ireland OR Irish OR The Netherlands OR Netherlands OR Dutch OR Germany OR German OR Spain OR Spanish OR Italy OR Italian OR Belgium OR Belgian OR Portugal OR Portuguese OR Malta OR Maltese OR Estonia OR Estonian OR Slovenia OR Slovenian OR "Czech Republic" OR Czech OR France OR French OR Poland OR Polish OR Hungary OR Hungarian OR Greece OR Greek OR Latvia OR Latvian OR Serbia OR Serbian)

AND

For peer review only

((MH “health personnel+”) OR (TI “health personnel” OR AB “health personnel”) OR (TI “care personnel” OR AB “care personnel”) OR (TI “health care provider” OR AB “health care provider”) OR (TI “health care providers” OR AB “health care providers”) OR (TI “care provider” OR AB “care provider”) OR (TI “care providers” OR AB “care providers”) OR (TI “health care staff” OR AB “health care staff”) OR (TI caregiver OR AB caregiver) OR (TI caregivers OR AB caregivers) OR (MH caregivers+) OR (TI “healthcare worker” OR AB “healthcare worker”) OR (TI “healthcare workers” OR AB “healthcare workers”) OR (TI “care assistant” OR AB “care assistant”) OR (TI “care aides” OR AB “care aides”) OR (MH nurses+) OR (TI nursing OR AB nursing) OR (TI nurse OR AB nurse) OR (TI nurses OR AB nurses) OR (MH physicians+) OR (TI physicians OR AB physicians) OR (TI physician OR AB physician) OR (TI physiotherapists OR AB physiotherapists) OR (MH “physical therapists+”) OR (TI “physical therapists” OR AB “physical therapists”) OR (TI psychologist OR AB psychologist) OR (MH “social workers+”) OR (TI “social workers” OR AB “social workers”) OR (TI “social worker” OR AB “social worker”) OR (TI “Health care professionals” OR AB “Health care professionals”) OR (TI “health care professional” OR AB “health care professional”) OR (TI nutritionist OR AB nutritionist) OR (TI “case managers” OR AB “case managers”) OR (MH “Medical staff+”) OR (TI “Medical staff” OR AB “Medical staff”) OR (TI “general practitioners” OR AB “general practitioners”) OR (MH “Physicians, Family+”))

AND

((TI “Person centered” OR AB “Person centered”) OR (TI “person centred” OR AB “person centred”) OR (TI “person centeredness” OR AB “person centeredness”) OR (TI personcenteredness OR AB personcenteredness) OR (TI “person centredness” OR AB “person centredness”) OR (TI “client centered” OR AB “client centered”) OR (TI “client centred” OR AB “client centred”) OR (TI “patient centered” OR AB “patient centered”) OR (TI “patient centred” OR AB “patient centred”) OR (MH “Patient Centered Care”) OR (TI “relationship centered” OR AB “relationship centered”) OR (TI “relationship centred” OR AB “relationship centred”) OR (TI “family centred” OR AB “family centred”) OR (TI “patient focused” OR AB “patient focused”) OR (TI “person focused” OR AB “person focused”) OR (TI “individualized care” OR AB “individualized care”) OR (TI “personalized care” OR AB “personalized care”) OR (TI “person-directed care” OR AB “person-directed care”) OR (TI “individual centered” OR AB “individual centered”)) AND ((MH perception+) OR (TI perception OR AB perception) OR (TI experience OR AB experience) OR (TI

For peer review only

experiences OR AB experiences) OR (TI perspective OR AB perspective) OR (MH attitude+) OR (TI attitude OR AB attitude) OR (TI attitudes OR AB attitudes) OR (TI "stress of conscience" OR AB "stress of conscience") OR (MH "Job Satisfaction+") OR (TI "job satisfaction" OR AB "job satisfaction") OR (MH Absenteeism+) OR (TI absenteeism OR AB absenteeism) OR (MH presenteeism+) OR (TI presenteeism OR AB presenteeism) OR (MH "Stress, Occupational+") OR (TI "occupational stress" OR AB "occupational stress") OR (MH "Personnel Turnover+") OR (TI "personnel turnover" OR AB "personnel turnover") OR (MH "Sick Leave+") OR (TI "sick leave" OR AB "sick leave") OR (MH "Stress, Psychological+") OR (TI "psychological stress" OR AB "psychological stress") OR (MH Dyssomnias+) OR (TI "sleep disorder" OR AB "sleep disorder") OR (TI "sleep disorders" OR AB "sleep disorders") OR (TI "sleep disturbances" OR AB "sleep disturbances") OR (MH "occupational health+") OR (TI "occupational health" OR AB "occupational health") OR (TI "moral stress" OR AB "moral stress") OR (MH "Attitude of Health Personnel+"))

AND

((MH "qualitative studies+") OR (TI "qualitative research" OR AB "qualitative research") OR (TI "qualitative study" OR AB "qualitative study") OR (TI "qualitative studies" OR AB "qualitative studies") OR (MH "focus groups+") OR (TI "focus groups" OR AB "focus groups") OR (TI "focus group" OR AB "focus group") OR (MH "Grounded theory+") OR (TI "grounded theory" OR AB "grounded theory") OR (TI mixed-method OR AB mixed-method) OR (TI ethnography OR AB ethnography) OR (TI phenomenology* OR AB phenomenology*) OR (TI interview* OR AB interview*) OR (MH "interviews+") OR (MH "Phenomenology") OR (TI hermeneutic* OR AB hermeneutic*))

For peer review only

Online Supplementary File 3 – Screening Tool

Screening Tool

Citation, Title, and Abstract Screening

1. Does the **citation** indicate publication on or after 2010?

    a. Yes or Unsure/Unclear: continue screening

    b. No: stop screening

2. Does the **title or abstract** use English?

    a. Yes or Unsure/Unclear: continue screening

    b. No: stop screening

3. Does the **title or abstract** indicate that the study uses a qualitative design?

    a. Yes or Unsure/Unclear: continue screening

    b. No: stop screening

Keywords: qualitative, interview, focus group, grounded-theory, mixed-method, ethnography, phenomenology, hermeneutic.

4. Does the **title or abstract** indicate that the study concerns PCC?

    a. Yes or Unsure/Unclear: continue screening

    b. No: stop screening

Note: This also includes the different definitions of PCC, e.g., Patient-Centred Care, Relationship-Centred Care, Client-Centred Care etc. (See block 3 in Search Strategy).

5. Does the **title or abstract** indicate that the study concerns HCPs perspectives/experiences of PCC?

    a. Yes or Unsure/Unclear: continue screening

    b. No: stop screening

6. Does the **title or abstract** indicate that the research was conducted in one of the countries from the inclusion criteria?

a. Yes or Unsure/Unclear: continue screening

b. No: stop screening

Countries: Sweden, Norway, Finland, Denmark, Iceland, United Kingdom (England, Scotland, Wales, North Ireland) Ireland, The Netherlands, Germany, Spain, Italy, Belgium, Portugal, Malta, Estonia, Slovenia, Czech Republic, France, Poland, Hungary, Greece, Latvia, Serbia.

7. Does the **title or abstract** indicate that job satisfaction/occupational health in relation to PCC was studied?

a. Yes or Unsure/Unclear: continue screening

b. No: stop screening

Keywords: satisfaction, job satisfaction, job strain, stress, moral stress, stress of conscience, demand, occupational health

Decision: **Should this article be included for full-text read?**

a. **Yes**, all 7 screening questions answered Yes or Unsure/Unclear

b. **No**, at least one answers definitely "No"

review only

Online Supplementary File 4 – SBU Checklist

Bedömning av studier med kvalitativ metodik

UPPDATERAD 2020-10-06

Författare: ______________________ År: ______________

Granskare: ____

Sammanvägd bedömning av metodologiska brister:
Obetydliga eller mindre ☐<br>Måttliga ☐
Stora brister, studien ingår inte i syntesen ☐
Kommentarer:

only

1. Överensstämmelse mellan filosofisk hållning/teori och urval och metodik i studien[1]

Vilken teori eller filosofisk hållning utgick författarna från?

Hänger syfte och fråga ihop med teori/filosofisk hållning?	Ja	Nej	Oklart
	☐	☐	☐

Kommentarer:

2. Deltagare

Hur gjordes urvalet?

**Stödfrågor för bedömning av brister i urvalsförfarandet:**	Ja	Nej	Oklart
Är urvalet lämpligt för att besvara frågan?	☐	☐	☐
Är rekryteringsmetoden lämpligt vald och genomförd?	☐	☐	☐
Finns det allvarliga brister som kan påverka tillförlitligheten?	☐	☐	☐

Kommentarer:

3. Datainsamling

Vilka metoder användes för datainsamling?

Finns det allvarliga brister i datainsamlingen som kan påverka tillförlitligheten?	Ja	Nej	Oklart
	☐	☐	☐

Kommentarer:

4. Analys

Vilka metoder användes för analys?

Stödfrågor för bedömning av brister i analyssteget:	Ja	Nej	Oklart
Är vald analysmetod lämplig och genomförd på ett lämpligt sätt?	☐	☐	☐
Var forskarna reflexiva vid tolkning av data?	☐	☐	☐
Validerades tolkningarna?	☐	☐	☐
Finns det allvarliga brister i analysen som kan påverka tillförlitligheten?	☐	☐	☐

Kommentarer:

5. Forskaren

Vilken bakgrund och kompetens hade forskarna?

Stödfrågor för bedömning av brister:	Ja	Nej	Oklart
Har forskarna någon relation till studiedeltagarna som kan påverka datainsamlingen?	☐	☐	☐
Har forskarna hanterat sin förförståelse på ett acceptabelt sätt?	☐	☐	☐
Var forskarna oberoende av finansiella eller andra förutsättningar som kunde påverka analysen?	☐	☐	☐
Finns det allvarliga brister som kan påverka tillförlitligheten?	☐	☐	☐

Kommentarer:

Online Supplementary File 5 – Quality Assessments

Quality assessment from the SBU checklist

Study	Data collection	Analysis	1. Theory/Philosophy: aim and research question coherent with theory or philosophical approach	2. Participants: relevant sampling; recruitment relevant and well described; ethical considerations relevant and well described	3. Data collection: relevant and well described	4. Data analysis: methodology relevant and well described; researchers showing reflexiveness in their analysis	5. The researcher: researcher–participant relationship well described; researcher pre-understanding well described; conflict of interest described	Methodological quality assessment
Barbosa *et al*[63]	Semi-structured focus groups	Thematic analysis	Yes	Yes	Yes/unclear	Yes	Yes	High
				Limitations: Describe that they ensure participants confidentiality and anonymity, but conducts focus groups where that cannot be ensured. Only focus groups.				
Boersma *et al*[60]	Focus groups	Deductive analysis from the RE-AIM framework	Yes	Yes	Yes/unclear	Yes	Yes/no	Moderate
				Limitations: Researcher-participant relation not described. Potential response bias discussed (with regards to focus group).				

Boström *et al*[49]	Focus group and individual semi-structured interviews	Qualitative content analysis	Yes	Yes	Yes	Yes	Yes/no	Moderate
			Limitations: Researcher-participant relation not described. Not discussing preunderstanding.					
Coyne[62]	Individual interviews with open-ended questions	Grounded theory	Yes	Yes	Unclear	Yes	Yes/no	Moderate
			Limitations: Researcher-participant relation not described. Split focus in the data between families/patients and HCPs.					
Fridberg *et al*,[50] 2021, Sweden	Focus groups, semi-structured dyadic interviews, and semi-structured individual interviews	Deductive–inductive content analysis	Yes	Yes	Unclear	Yes	Yes	Moderate
			Limitations: Potential data collection bias.					
Kadri *et al*[56] (initial study: Rapaport *et al*[81])	Semi-structured individual interviews	Secondary qualitative thematic analysis	Yes	Yes	Unclear	Yes	Yes/no	Moderate
			Limitations: Initial study not having the precise focus relevant for this study which means data could have been more precise, the secondary analysis however relevant for this review. Preunderstanding not described.					
Karlsson *et al*[51]	Semi-structured individual interviews	Systematic text condensation	Yes	Yes	Yes	Yes	Yes/no	High
			Limitations: Declaration of interest not described.					

Kirkley *et al*[59]	Focus groups, telephone interview, individual interviews	Qualitative thematic analysis	Yes	Yes/no	Yes	Yes	Yes/no	Moderate	Limitations: Potential selection bias in the face-to-face interviews. Participants worked in departments with good knowledge in PCC whereas in the telephone interviews the knowledge varied. Relation with participants not described.
Kjörnsberg *et al*[53]	Open-ended informal individual interviews	Latent qualitative content analysis	Yes	Yes/no	Yes	Yes	Yes/no	Moderate	Limitations: Small sample of only 6 RNs. Conflict of interest not described.
Nilsson *et al*[52]	Individual semi-structured interviews	Qualitative content analysis	Yes	Yes	Yes	Yes	Yes/no	Moderate	Limitations: Researcher-participant relationship not described. Preunderstanding not described.
Pinkert *et al*[64]	Semi-structured focus groups	Secondary qualitative content analysis	Yes	Yes	Yes/unclear	Yes	Yes/no	Moderate	Limitations: Cannot find researcher-participant relation due to not getting access to the
Ross *et al*[57]	Individual semi-structured interviews	Framework analysis	Yes	Yes	Yes	Yes	Yes/no	High	Limitations: Researcher-participant relationship not described.
Sjöberg and Forsner[54]	Focus groups, semi-structured individual interviews	Analysis in phenomenography	Yes	Yes/unclear	Yes	Yes	Yes/no	Moderate	Limitations: Relatively low sample size (n = 7). Researcher-participant relationship not described.

Uittenbroek *et al*[61]	Individual interviews with topic-based interview guide	Grounded theory	Yes	Yes/no	Yes	Yes	Yes/no	High
				Limitations: Only female participants. Researcher-participant relationship not described.				
Vassbø *et al*[65]	Individual open-ended interviews	Phenomenological-hermeneutical analysis for research of lived experiences	Yes	Yes/no	Yes	Yes	Yes/no	Moderate
				Limitations: Only female participants. Relationship between some researchers and participants that could lead to potential bias.				
Walker and Deacon[58]	Group interviews and an individual interview	Qualitative directed content analysis	Yes	Yes	Yes/no	Yes	Yes/no	Moderate
				Limitations: Researcher-participant relationship not described.				
Öhman *et al*[55]	Individual interviews with thematic interview guide	Grounded theory, qualitative content analysis	Yes	Yes	Yes	Yes	Yes/no	High
				Limitations: Preunderstanding not described. Researcher-participant relationship not described.				

BMJ Open

Healthcare professionals' experiences of job satisfaction when providing person-centred care: a systematic review of qualitative studies

Journal:	*BMJ Open*
Manuscript ID	bmjopen-2022-071178.R1
Article Type:	Original research
Date Submitted by the Author:	14-Apr-2023

Complete List of Authors:	Gustavsson, Kristoffer; University of Gothenburg Centre for Person-Centred Care<br>van Diepen, Cornelia; University of Gothenburg Centre for Person-Centred Care; Erasmus Universiteit Rotterdam Erasmus School of Health Policy and Management,<br>Fors, Andreas; University of Gothenburg Centre for Person-Centred Care; University of Gothenburg Institute of Health and Care Sciences<br>Axelsson, Malin; Malmö University Department of Care Science<br>Bertilsson, Monica; University of Gothenburg School of Public Health and Community Medicine<br>Hensing, Gunnel; University of Gothenburg School of Public Health and Community Medicine

**Primary Subject Heading**:	Occupational and environmental medicine
Secondary Subject Heading:	Patient-centred medicine, Public health, Qualitative research, Health services research, Mental health
Keywords:	PREVENTIVE MEDICINE, PUBLIC HEALTH, Quality in health care < HEALTH SERVICES ADMINISTRATION & MANAGEMENT, SOCIAL MEDICINE

SCHOLARONE™
Manuscripts

I, the Submitting Author has the right to grant and does grant on behalf of all authors of the Work (as defined in the below author licence), an exclusive licence and/or a non-exclusive licence for contributions from authors who are: i) UK Crown employees; ii) where BMJ has agreed a CC-BY licence shall apply, and/or iii) in accordance with the terms applicable for US Federal Government officers or employees acting as part of their official duties; on a worldwide, perpetual, irrevocable, royalty-free basis to BMJ Publishing Group Ltd ("BMJ") its licensees and where the relevant Journal is co-owned by BMJ to the co-owners of the Journal, to publish the Work in this journal and any other BMJ products and to exploit all rights, as set out in our licence.

The Submitting Author accepts and understands that any supply made under these terms is made by BMJ to the Submitting Author unless you are acting as an employee on behalf of your employer or a postgraduate student of an affiliated institution which is paying any applicable article publishing charge ("APC") for Open Access articles. Where the Submitting Author wishes to make the Work available on an Open Access basis (and intends to pay the relevant APC), the terms of reuse of such Open Access shall be governed by a Creative Commons licence – details of these licences and which Creative Commons licence will apply to this Work are set out in our licence referred to above.

Other than as permitted in any relevant BMJ Author's Self Archiving Policies, I confirm this Work has not been accepted for publication elsewhere, is not being considered for publication elsewhere and does not duplicate material already published. I confirm all authors consent to publication of this Work and authorise the granting of this licence.

For peer review only

Title: Healthcare professionals' experiences of job satisfaction when providing person-centred care: a systematic review of qualitative studies

Kristoffer Gustavsson (ORCID id: 0000-0002-1743-9576),[1] Cornelia van Diepen (0000-0001-6991-9443),[1,2] Andreas Fors (0000-0001-8980-0538),[1,3,4] Malin Axelsson (0000-0001-5493-8334),[5] Monica Bertilsson (0000-0003-2365-2522),[6] Gunnel Hensing (0000-0002-3457-2993)[6]

[1] Centre for Person-Centred Care (GPCC), University of Gothenburg, Gothenburg, Sweden

[2] Erasmus School of Health Policy & Management, Erasmus University Rotterdam, Rotterdam, Netherlands

[3] Institute of Health and Care Sciences, Sahlgrenska Academy, University of Gothenburg, Goteborg, Sweden

[4] Region Västra Götaland, Research, Education, Development and Innovation, Primary Health Care, Gothenburg, Sweden

[5] Department of Care Science, Faculty of Health and Society, Malmö University, Malmö, Sweden

[6] School of Public Health and Community Medicine, Institute of Medicine, Sahlgrenska Academy, University of Gothenburg, Gothenburg, Sweden

Corresponding author:
Andreas Fors
Andreas.fors@gu.se

University of Gothenburg, Sahlgrenska Academy, Institute of Health and Care Sciences
Arvid Wallgrens backe, building 1
Box 457, 405 30 Gothenburg, Sweden

Word count: 3982 (excluding title page, abstract, references, figures and tables)

For peer review only - http://bmjopen.bmj.com/site/about/guidelines.xhtml

ABSTRACT

Objectives: This qualitative systematic review aimed to explore and synthesise healthcare professionals' (HCPs) experiences of job satisfaction when providing person-centred care (PCC) in healthcare settings in Europe.

Method: This systematic review of qualitative studies was followed by a thematic synthesis applying an inductive approach. Studies concerning HCPs and different levels of healthcare in Europe were eligible for inclusion. The CINAHL, Pubmed, and Scopus databases were searched. Study titles, abstracts, and full texts were screened for relevance. Included studies were assessed for methodological quality using a quality appraisal checklist. Data were extracted and synthesised via thematic synthesis, generating analytical themes.

Results: Seventeen studies were included in the final thematic synthesis, and eight analytical themes were derived. Most studies were conducted in Sweden and the United Kingdom and were performed in hospitals, nursing homes, elderly care, and primary care. Thirteen of these studies were qualitative and four used a mixed-method design in which the qualitative part was used for analysis. HCPs experienced challenges adapting to a new remoulded professional role and felt torn and inadequate due to ambiguities between organisational structures, task-oriented care, and PCC. Improved job satisfaction was experienced when providing PCC in line with ethical expectations, patients and colleagues expressed appreciation, and team collaboration improved, while learning new skills generated motivation.

Conclusion: This systematic review found varied experiences among HCPs. Notably, the new professional role was experienced to entail disorientation and uncertainty; importantly, it also entailed experiences of job satisfaction such as meaningfulness, an improved relationship between HCPs and patients, appreciation, and collaboration. To facilitate PCC implementation, healthcare organisations should focus on supporting HCPs through collaborational structures, and resources such as time, space and staffing.

PROSPERO registration number: CRD42022304732

ARTICLE SUMMARY

Strengths and limitations of this study:

For peer review only - http://bmjopen.bmj.com/site/about/guidelines.xhtml

- This is the first systematic review synthesising qualitative research on healthcare professionals' experiences of job satisfaction when providing person-centred care across different healthcare settings in Europe.
- The methodology was thoroughly described, increasing replicability and reliability, and the researchers involved in this review had different professional backgrounds, i.e., nursing, sociology, and social medicine.
- The search strategy was extensive, included a broad range of terms, and covered several databases; a search specialist was consulted in the process.
- The search was limited to studies written in English and published in or since 2010, so some potentially relevant studies might have been missed.
- The included studies had different qualitative methodologies and theoretical approaches, which could affect interpretations and comparisons.

Keywords: healthcare professional, job satisfaction, person-centred care, qualitative synthesis, systematic review

For peer review only

For peer review only - http://bmjopen.bmj.com/site/about/guidelines.xhtml

INTRODUCTION

In Europe, healthcare professionals (HCPs) are at higher risk of exposure to psychosocial risk factors from their work environment than are several other occupations,[1] which can result in adverse health impacts on the individual, such as stress, fatigue, burnout, and physical and emotional demands.[2] The working environment affects job satisfaction, which is a mental state of satisfaction deriving from employees' assessment of their work situation.[3] It includes factors such as accomplishment, praise, social relations, advancement opportunities, and undertaking tasks in line with personal ethical values.[4 5] Low job control can decrease health and job satisfaction,[6] and high stress combined with decreased job satisfaction is associated with high turnover rates in healthcare.[7] With demands on European healthcare systems gradually increasing,[8] recruiting and retaining educated HCPs is difficult, threatening the sustainability of healthcare.[9] Moreover, an under-supported workplace with many stressors can lead to decreased quality of care for patients and increased emotional demands on HCPs.[10]

Simultaneously, European healthcare has been evolving to involve more patient participation.[11] Person-centred care (PCC) is a model of care deriving from an ethical standpoint in which patients' capabilities and needs are emphasised, and care is provided through collaboration between professionals, patients, and family.[12 13] Providing PCC involves some key principles, such as: initiating and focusing partnership by carefully listening to the patient's narrative, with the patient seen as an expert on her/his health; and co-creating a health plan covering the patient's experiences, capabilities, goals, expectations, and social support resources along with medical status, and modifying the health plan according to the circumstances.[12 14 15] PCC communication involves both verbal (e.g., open-ended questions, reflections, and summaries) and non-verbal communication (e.g., displaying welcoming and respectful body language).[14 16]

Working with PCC based on the patient's narrative helps professionals realise their expected ethical standards and provide high-quality care.[17] Working in a more person-centred way requires conducive organisational structure and support, leadership and training, and constructive collaboration within the interprofessional team.[18 19] Several concepts have been used to describe care centred around the patient and her/his family, such as patient centredness or patient-centred care[20 21] or relationship-centred,[22] family-centred,[23] and individualised care,[24] and varied settings require specific approaches of 'centredness' on the

For peer review only - http://bmjopen.bmj.com/site/about/guidelines.xhtml

patient and family.[25 26] PCC entails building a relationship in which patients are involved as active partners in their care and treatment and, more distinctly, emphasises a shift away from a model of care in which patients are often regarded as passive care recipients.[13 15]

In European healthcare, ethical stress and lack of resources are issues affecting HCPs' ability to offer the desired care of the required quality.[27] PCC has been proposed as a model of care with potential to help solve some of these problems, although evidence regarding PCC and its impact on HCP outcomes remains unclear.[28] PCC implementation being increasingly integrated into healthcare systems internationally will expose more HCPs to this model of care. Findings from this systematic review can help enhance the understanding of how HCPs' job satisfaction relates to PCC, and lead to further research and new relevant policies to improve working conditions in healthcare.

Research has primarily investigated the patient outcomes of PCC, showing, for example, increased self-efficacy, improved satisfaction with care, improved symptom control, and clinical outcomes characterised by shorter hospital stays and cost savings.[29] Five reviews of aspects of HCP outcomes and PCC have been identified,[30-35] which presented mixed findings. These reviews focused on specific professions, such as registered nurses (RNs), nurse aides, direct-care workers, and caregivers in residential care or nursing homes,[30-32 34 35] or included only quantitative studies.[30 31 33-35] No reviews have explored qualitative research on job satisfaction from providing PCC across healthcare professions and settings in Europe; this systematic review accordingly aimed to fill this research gap.

AIM

This qualitative systematic review aimed to explore and synthesise HCPs' experiences of job satisfaction when providing PCC in healthcare settings in Europe.

METHODS

Design

A systematic review of qualitative studies was conducted. The research question was 'How do healthcare professionals in Europe, when providing PCC, experience their job satisfaction?' A protocol following the PRISMA-P checklist was written in preparation for the systematic review and registered in PROSPERO on 23 January 2022 (ID: CRD42022304732).[36] A systematic search was executed on 21 December 2021 in the electronic databases CINAHL,

For peer review only - http://bmjopen.bmj.com/site/about/guidelines.xhtml

Pubmed, and Scopus, covering the main research areas relevant to this review. An updated search, using the same search strategy for studies published in the period between January 2021 – March 2023 was conducted in March 2023. Thematic synthesis with an inductive approach was used to analyse data obtained from the relevant studies.[37] The framework for Enhancing Transparency in Reporting the Synthesis of Qualitative Research (ENTREQ)[38] was used for reporting the synthesis (see Online Supplementary File 1).

Eligibility criteria

The eligibility criteria for the literature search in this systematic review are presented in table 1, and were based on the Population, Exposure, Outcome (PEO) framework.[39] The inclusion criterion for the population was HCPs, for exposure it was PCC, and for the outcome it was experiences of job satisfaction. Additionally, studies from all healthcare settings were acceptable, provided they were conducted in European countries with a healthcare system that could be identified according to Rosengren *et al.*[40] Studies had to be qualitative, peer-reviewed, ethically approved, written in English, and published in or since 2010. The included qualitative studies also include the qualitative, but no quantitative, parts of mixed-method studies. The exclusion criteria were quantitative studies and reviews.

Table 1 Eligibility criteria

Inclusion criteria	Exclusion criteria
• Healthcare professionals (Population)<br>• Person-centred care (Exposure)<br>• Experiences of Job Satisfaction (Outcome)<br>• All healthcare settings<br>• Studies conducted in European countries with identified healthcare systems according to Rosengren *et al.*[40]<br>• Qualitative, peer-reviewed, and ethically approved studies, written in English<br>• Published in or since 2010	• Quantitative studies<br>• Reviews

Search strategy

For peer review only - http://bmjopen.bmj.com/site/about/guidelines.xhtml

The search strategy was created by the research group with assistance from a librarian at the Biomedical Library at the University of (name withheld during the review process). It comprised keywords and Medical Subject Heading (MeSH) terms hierarchically structured to facilitate the inclusion of broader and more precise medical and health-related terms.[41] In accordance with the PEO framework, relevant search terms associated with HCPs, PCC, and job satisfaction were defined and put in their associated search blocks.[39] Within each block, the search terms were connected using the Boolean operator 'OR', and the blocks were connected with the Boolean operator 'AND'.[42] Along with the PEO blocks, two additional blocks were added. The first additional search block, 'countries', consisted of the European countries eligible for inclusion, similar to Rosengren *et al*[40] and their mapping of European countries having identifiable and eligible healthcare systems. Rosengren *et al* found 23 eligible European countries and identified three types of healthcare systems: Beveridge (n = 12), Bismarck (n = 10), and Out of Pocket (n = 1).[40] The second additional block was 'study design', which comprised different search terms for qualitative study designs and mixed-method studies (e.g., interviews and focus groups) to ensure that the search would identify all relevant studies of qualitative design. The search strategy is presented in Online Supplementary File 2.

Selection process

The selection of studies was conducted between 25 January 2022 until 11 March 2022. The 3754 records from the database searches were imported into Endnote for reference management and into Rayyan software for screening. The first (A1) and second author (A2) screened titles and abstracts blinded and independently for relevance in Rayyan using a screening tool (see Online Supplementary File 3).[43] The 'detect duplicates' option available in both programs was used to remove 872 duplicates.[44 45] The remaining 2882 studies were screened for title and abstract, and 2807 of those were excluded. Out of the 75 studies, one was not retrieved in full-text. In the second step of the selection process, the remaining 74 studies were downloaded and shared in an Endnote library. The studies were read in full text for relevance against the inclusion criteria by A1 and A2 independently in Endnote. All full-text studies were discussed in a meeting among A1, A2, and A3 until consensus was reached. Fifty six studies were excluded due to incorrect outcome, exposure, or design, or by being conducted in the wrong location, or not being written in English. Eighteen studies were

For peer review only - http://bmjopen.bmj.com/site/about/guidelines.xhtml

considered relevant to the review. After closer reading, one study was interpreted as related to working during the COVID-19 pandemic rather than to person-centred work; therefore, it was excluded at this stage after discussions. The selection process is presented in a PRISMA flow diagram (Figure 1).[46]

Figure 1. The selection of studies presented in a PRISMA flow diagram.[46]

For the updated literature search conducted in March 2023, the same electronic databases and search strategy were used, but the year of publication was limited to 2021-2023. The records were imported into Endnote and screened for relevance by A1. Two relevant studies were identified, which will be incorporated in the discussion section.

Quality assessment

The Swedish Agency for Health Technology Assessment and Assessment for Social Services (SBU) checklist for assessing methodological quality was used to assess the included studies after they were read in full text.[47] This checklist has five domains (i.e., theoretical framework, sampling, data collection, analysis, and researcher role) and 13 items integrated among the five domains (see Online Supplementary File 4).[48]

The studies were initially assessed by A1, and secondly independently assessed by A6, who is a senior researcher and Professor. Both A1 and A6 considered omitting studies indicating low methodological quality. All studies considered relevant were assessed as having moderate or high quality by both assessors. Items in the five domains were related to methodological choices, such as if the aim was coherent with regard to the theoretical stance, if the recruitment was suitable and well conducted, or if the method of analysis was appropriate and carried out properly. The items could be answered with "yes", "no" or "unclear". The combined item answers regarding whether it had serious methodological concerns affecting the reliability of the study resulted in a domain grading of "yes" "no" or "unclear" (see Online Supplementary File 4). The gradings from the five domains were then combined and given an overall rating of the study as follows: no or few concerns = high quality; moderate concerns = moderate quality; and high concerns = low quality, in line with the recommendations of SBU.[49] The studies' ratings varied between moderate (n = 12) and high (n = 5) quality. The most common deficiencies were missing descriptions of the researcher's role and pre-understanding, and how it might have affected the study results. No studies were assessed as

For peer review only - http://bmjopen.bmj.com/site/about/guidelines.xhtml

having low methodological quality, so no studies were excluded due to the quality assessment. The quality assessments are presented in Online Supplementary File 5.

Data extraction and synthesis

The qualitative findings extracted from the included studies were analysed via thematic synthesis. The synthesis followed the three stages described by Thomas and Harden.[37] The synthesis was conducted by A1 supported by A6. All authors then reviewed and discussed the synthesis and themes.

The first stage of the thematic synthesis was coding, which was conducted using NVivo software, in which the text was coded line by line to identify passages of text from the studies in line with the aim and research question of the review. Each text passage received a code describing its meaning. The coding was continuously scrutinised: it was revised with further coding if consistency was lacking, or progressed without revision if no issues emerged.[37]

The second stage involved inductively developing descriptive themes from the data. In this stage, a descriptive theme was formulated to cover the first code from the first study; either the following codes from the same study were grouped under that theme, if the codes were related in meaning and concept, or new descriptive themes were formulated when codes with new thematic concepts emerged. Codes from subsequent studies were then assigned to existing descriptive themes or new themes were formulated, if needed.[37]

The third stage of the process 'went beyond' the content of the primary studies and created a new understanding of the topic. New knowledge was created by utilising the descriptive themes generated and connecting them to the research question to produce analytical themes. The analytical themes were discussed by A1 and A6, and new themes were created if necessary and scrutinised again until they were considered to sufficiently describe the descriptive themes.[37]

Patient and public involvement

This study was conducted without patient or public involvement.

RESULTS

Characteristics of included studies

For peer review only - http://bmjopen.bmj.com/site/about/guidelines.xhtml

The study aimed to explore HCPs' experiences of job satisfaction when providing PCC. The characteristics of the 17 included studies are presented in Online Supplementary File 6. Seven studies were conducted in Sweden,[50-56] four in the United Kingdom (UK),[57-60] two in the Netherlands,[61 62] one in Ireland,[63] one in Portugal,[64] one in Austria and Germany,[65] and one in Sweden, Norway, and Australia.[66]

Most of the studies were qualitative (*n* = 13),[50-55 57-59 62 63 65 66] while four[56 60 61 64] used mixed methods. Eight studies[52 54 56-58 62 63 66] used individual interviews, three[61 64 65] used focus groups, and six[50 51 53 55 59 60] combined individual and focus-group interviews, of which two[51 59] also used dyadic interviews.

The total number of participants in the included studies was 459 (range 6–97).[51 54] Thirteen studies[50-57 61-64 66] reported the gender of participants, who were predominantly women (*n* = 351; 90%). The professions represented were RNs and specialist nurses (*n* = 211),[50-54 56 58 59 62 63 65 66] assistant nurses, students, enrolled nurses, caregivers, direct-care workers, frontline staff (*n* = 189),[51 53 57 58 60 61 64 66] physiotherapists (*n* = 23),[51 55 56 66] occupational therapists (*n* = 15),[51 56 66] physicians (*n* = 10),[51] professional support workers and social workers (*n* = 8),[58 62] and 'other', with the profession not being specified (*n* = 1).[51] Nine studies were conducted in hospitals, [51-55 58 59 63 65] six in elderly-care, residential-care, or nursing homes,[56 57 60 61 64 66] and two in primary-care or GP practices (an organisation of one or more General Practitioners).[50 62]

For peer review only - http://bmjopen.bmj.com/site/about/guidelines.xhtml

Synthesis

Twenty-four descriptive themes were derived from the codes, and eight analytical themes from the descriptive themes (Table 2). The analytical themes were generated by going beyond the content of the primary studies, representing interpretations of the included studies as a whole.[37]

Table 2 Themes from the qualitative synthesis

Analytical themes	Descriptive themes	Studies included in themes
HCPs feeling torn and inadequate	Conflict between organisational structures and PCC<br>Conflict between disease-oriented task care and PCC<br>Feelings of inadequacy and guilt	Coyne,[63] Fridberg *et al*,[51] Kadri *et al*,[57] Kjörnsberg *et al*,[54] Pinkert *et al*,[65] Uittenbroek *et al*,[62]
PCC demanding for HCPs	High workloads hamper the work with PCC<br>PCC not affecting or is negative for workload<br>Stressful due to lack of resources	Boersma *et al*,[61] Boström *et al*,[50] Coyne,[63] Fridberg *et al*,[51] Kadri *et al*,[57] Karlsson *et al*,[52] Kjörnsberg *et al*,[54] Ross *et al*,[58] Sjöberg and Forsner,[55] Uittenbroek *et al*,[62]
Remoulded professional role	Disorientation and uncertainties with new approach and routines<br>Loss of control	Boström *et al*,[50] Fridberg *et al*,[51] Kadri *et al*,[57] Sjöberg and Forsner[55]

For peer review only - http://bmjopen.bmj.com/site/about/guidelines.xhtml

Patients having considerable influence		
Providing PCC meaningful for HCPs	Improved job satisfaction<br>Meaningfulness – making a difference and doing the 'little extra'<br>In line with ethical expectations<br>Improved relationship between HCPs and patients<br>Self-care for improved care	Barbosa *et al*,[64] Boersma *et al*,[61] Fridberg *et al*,[51] Karlsson *et al*,[52] Kjörnsberg *et al*,[54] Nilsson *et al*, [53] Pinkert *et al*,[65] Ross *et al*,[58] Vassbø *et al*,[66] Öhman *et al*,[56]
HCPs feeling appreciated	Gratitude from patients makes working with PCC easier<br>Feeling valued and appreciated by colleagues	Barbosa *et al*,[64] Boersma *et al*,[61] Nilsson *et al*, [53] Uittenbroek *et al*,[62] Vassbø *et al*,[66] Öhman *et al*[56]
Improved team collaboration	Enhanced collaboration improves satisfaction and working context<br>Social support reduces stress and frustration	Boersma *et al*,[61] Kirkley *et al*,[60] Nilsson *et al*, [53] Uittenbroek *et al*,[62] Vassbø *et al*[66]
Workload and stress reduction	Improves flow and workload<br>Reduced stress at work<br>Sense of increased control and reduced anxiety	Barbosa *et al*,[64] Fridberg *et al*,[51] Kjörnsberg *et al*,[54] Vassbø *et al*[66]
Increased personal motivation and commitment	Feeling joy and energy<br>Thriving at work	Barbosa *et al*,[64] Fridberg *et al*,[51] Kadri *et al*,[57] Karlsson *et al*,[52] Vassbø *et al*,[66] Walker and Deacon[59]

For peer review only - http://bmjopen.bmj.com/site/about/guidelines.xhtml

Willingness and motivation to perform ‘ideal’<br>PCC

For peer review only

For peer review only - http://bmjopen.bmj.com/site/about/guidelines.xhtml

Analytical themes

HCPs feeling torn and inadequate

Working in line with PCC was experienced as challenging and could make HCPs feel torn and inadequate. The reason for this was described as a conflict between the desire to perform personalised care fulfilling ethical and professional standards, organisational deficiencies in support and negotiation, and an organisational focus on effectiveness.[54 57 62 63 65] A perceived conflict between disease-oriented task care and PCC was described, with HCPs initially uncertain whether, for example, listening attentively was regarded as a task.[62] HCPs who experienced difficulties balancing PCC with the more task-oriented disease care perceived that they were blamed by others, resulting in feelings of guilt and inadequacy.[51 54]

PCC demanding for HCPs

HCPs described how spending time with patients when providing PCC could be demanding and be hampered by high workloads in the organisation.[50 52 55 63] HCPs found that PCC resulted in the same or even a greater workload than before.[51 54 58 62] HCPs also perceived that it could be stressful to work with PCC due to pressures from insufficient resources in terms of time, space, and staffing.[51 57 63] Some HCPs found that PCC did not influence their job satisfaction, especially when dealing with difficult behaviours of patients, such as when they did not respond well.[61]

Remoulded professional role

HCPs experienced a remoulded professional role when providing PCC. This could result in disorientation and uncertainty due to new structures, routines, and adaptation to new ways of working.[50 55] They perceived their expertise to be compromised and experienced a loss of control, with patients now having more influence over their own care.[55 57] Moreover, HCPs experienced doubt concerning parts of their new role, which was more conversation based than before and felt less valid than the usual, more hands-on approach.[55] Providing PCC could be frustrating with patients who were less communicative and more reserved[55] and in situations in which HCPs did not agree with patients' wishes regarding their care.[51]

Providing PCC meaningful for HCPs

When HCPs could provide PCC in line with ethical expectations and could collaborate with patients, they experienced increased meaningfulness and job satisfaction.[52 53 56 58 65 66] PCC provision was aligned with HCPs' standards and expectations regarding ethics and routines, such as providing equitable and inclusive care, discussing goals and writing health plans with

For peer review only - http://bmjopen.bmj.com/site/about/guidelines.xhtml

patients.[51] Spending more time with patients improved the relationship between HCPs and patients.[51 52 54 58 61 66] Additionally, HCPs described becoming more aware of the importance of their personal well-being when providing PCC, through its connection to patient well-being. HCPs taking better care of themselves could enable care that both patients and HCPs were content with.[64]

HCPs feeling appreciated

Although some HCPs found that PCC did not influence their job satisfaction when dealing with patients presenting difficult behaviours, others found that providing PCC instead helped in the care of these patients, causing them to show appreciation.[61] Patient expressions of gratification with the care were experienced as leading to increased job satisfaction and enhanced energy.[56 61 66] PCC was also described as a practice leading to appreciation from colleagues, as colleagues could understand and acknowledge one another more through collaboration and interaction, increasing satisfaction and meaningfulness at work.[53 62 64 66]

Improved team collaboration

The enhanced awareness, learning, and collaboration with colleagues and organisations derived from PCC was described as improving job satisfaction and the work environment.[61 62 66] HCPs valued the social support that emerged from working with PCC, fostering a supportive culture in which colleagues shared experiences and feelings, and recognised and relieved each other; this was experienced as increasing job satisfaction and reducing stress and frustration.[53 60 66]

Workload and stress reduction

HCPs who implemented PCC experienced a better workflow with new routines[51] and a calmer work environment.[66] Lower workload and reduced stress were experienced from working with PCC and being responsible for fewer patients.[54] Moreover, HCPs described increased control arising from enhanced independence in organising the work.[66] With PCC, HCPs could better deal with situations formerly experienced as stressful, which was described as reducing anxiety.[64]

Increased personal motivation and commitment

Motivation[57 66] and commitment[52 57 59 64] to continue providing PCC increased. The HCPs experienced enhanced joy from the improved connections with patients[51] and increased energy when they could meet patients' needs.[66] Moreover, HCPs working jointly with PCC,

For peer review only - http://bmjopen.bmj.com/site/about/guidelines.xhtml

helping one another and discussing the care, generated motivation and an increased striving to improve their current skills.[66]

DISCUSSION

This systematic review identified, assessed, and synthesised qualitative studies to explore HCPs' experiences of providing PCC by focusing on job satisfaction. In the updated literature search, two additional relevant qualitative studies by Allerby *et al*,[68] and Petersson *et al*[69] were identified. Findings from these studies were in line with findings from this systematic review and will be incorporated into the following discussion.

An interesting finding derived from the synthesis concerned the remoulded professional role. A study by Boström *et al*[70] of RNs' experiences of providing PCC over the phone highlighted that RNs experienced a need to adapt and remould their professional role when working with PCC. Remoulding the professional role was an iterative process in which they had to challenge their thinking and practice, which could generate insecurity;[70] despite being a challenging process, the RNs experienced this remoulding as expanding their professional role.[70] HCPs experiencing development of the professional role when working with PCC was also described in the study by Allerby *et al*.[68] In this systematic review, uncertainty towards the new professional role when providing PCC emerged due to the new practice and routines, and an experienced decrease in control and autonomy.[50 51 55 57] Having a system with a PCC culture with well-supported and committed HCPs is a prerequisite for implementing PCC.[71] Moreover, Moore *et al*[26] described how HCPs must adopt a new professional role when working with PCC. HCPs' integration of PCC is a process that requires time for reflecting on and adapting to the theory and practice of PCC for the specific context.[14] A more equal distribution of power, with patients being more involved and seen as experts in their own care, could be experienced as challenging for HCPs used to traditional care. Summer Meranius *et al*[72] argued that PCC could lead to reduced autonomy and negative health impacts for HCPs. To avoid negative health impacts, overcome the challenges of adapting, and instead expand the professional role, HCPs should be provided with time for the process and be encouraged to reflect on the theory and practice of PCC in their specific setting.

HCPs unable to provide the care desired from an ethical standpoint were also apparent. Similar to the finding of Juthberg and Sundin,[73] guilt and inadequacy could arise from being torn between striving to provide PCC, task-oriented care, and organisational structures.[51 54 55]

For peer review only

For peer review only - http://bmjopen.bmj.com/site/about/guidelines.xhtml

[57 62 63 65] This was also described in the study by Peterson *et al*,[69] where care structures requiring tasks being carried out in high tempo could inhibit PCC. A review by Güney *et al*[32] showed that the organisation and task-oriented care could be barriers to PCC and that improved support could modify organisational barriers. Ethical stress can also emerge from not being able to provide the care desired due to a lack of resources.[27] Furthermore, perceived stress and job dissatisfaction can increase healthcare turnover rates.[7] However, systematic organisational support when providing PCC can reduce stress of conscience,[74] indicating that systematic organisational support could serve as a preventive measure. Providing resources for HCPs working with PCC could thus potentially reduce stress of conscience and improve job satisfaction; in the long term, this might lead to reduced turnover, with HCPs being more satisfied with the care provided.

HCPs also experienced improved job satisfaction and meaningfulness when providing PCC, being engaged, and spending time with patients,[51-54 56 58 61 65 66] and they described reduced workload and stress from working with PCC.[51 54 66] The mixed experiences found in this review regarding engagement, workload, and stress align with Summer Meranius *et al*,[72] suggesting that engagement in patient care is essential for PCC and enhances the relationship between HCPs and patients; however, too much engagement and commitment can also be considered a risk.[72] The contrasting views of workload and stress may be mediated by organisational support. Whereas reduced stress seemed to be related to resources such as enhanced support, increased stress was related to a lack of resources in term of space, time, and staffing. Structures promoting collaboration with colleagues and more time with patients facilitate a conducive working environment, promoting PCC.[71] This underpins the benefits of supporting HCPs with sufficient resources to provide PCC. Moreover, it aligns with WHO's efforts to promote the co-creation of care in the interest of improved working conditions.[75]

The HCPs felt increased appreciation from colleagues when providing PCC.[53 62 64 66] Additionally, improved team collaboration when providing PCC was described as leading to enhanced satisfaction with the work environment.[53 61 62 66] The findings regarding those themes are well aligned with Montgomery *et al*,[76] who found a positive association between well-functioning teamwork and engagement. Similarly, Allerby *et al*[68] found that PCC increased satisfaction for HCPs from the improved work environment, communication and team collaboration. This suggests that collaboration in providing PCC could have a role in improving the health and well-being of HCPs.

For peer review only - http://bmjopen.bmj.com/site/about/guidelines.xhtml

As in the review by van den Pol-Grevelink,[31] increased thriving, motivation, and commitment were also apparent.[52 57 59 64 66] Keyko *et al*[77] found that HCPs engaged in their work will experience increased motivation and well-being, resulting in higher-quality care. Moreover, healthy HCPs are a prerequisite for the consistent provision of PCC.[78] HCPs increasingly valued their personal well-being, which meant that they could provide better, more satisfying care.[64] HCPs providing care that helps them thrive and be satisfied could mean healthier HCPs and decreased turnover in healthcare, facilitating the continuous provision of PCC.

Strengths and limitations

A strength of this review was that its authors had different professional backgrounds, with A1, A3, and A4 coming from nursing, A2 from sociology, and A5 and A6 from social medicine. In the selection and research process, pre-understanding was addressed through regular discussions in the group. Moreover, a librarian was consulted, several databases were searched, a broad range of terms was used, and the reference lists of the included studies were scrutinised. Also, two authors independently screened the titles, abstracts, and full texts for inclusion.

A systematic review was a suitable method for the present research purpose, since the aim was to explore and synthesise findings regarding a particular research question.[79] To demonstrate transparency, reduce the risk of bias, and enable replicability, a PRISMA protocol was published in PROSPERO before data collection, a PRISMA flow diagram was used, and data from extracted studies were presented in tables.[46 80-82] It was a strength that the synthesis strictly followed the steps described by Thomas and Harden,[37] since these have been proven viable for thematically synthesising qualitative experiences of healthcare implementations. The themes derived from the qualitative synthesis can be valuable as guidance for further research or can inform policy and practice concerning HCPs' job satisfaction.[79 83]

Some potentially relevant studies might not have been captured since only English-language studies, specific search terms, and studies published in or since 2010 were included. Recent studies are more likely to capture the current healthcare situation. Additionally, the literature search was performed on 21 December 2021, and studies published after that date were not included in the synthesis of this review. However, an updated search was conducted in March 2023 to capture relevant studies published after the initial literature search.

For peer review only

For peer review only - http://bmjopen.bmj.com/site/about/guidelines.xhtml

The search was delimited regarding the countries included in the eligibility criteria. Two studies were included despite being partly conducted in countries not included in the criteria, which could be considered a limitation. Although Vassbø *et al*[66] collected some of the data in Australia, two of the three countries represented in the data collection (i.e., Sweden and Norway) fit the inclusion criteria and the study contained rich data, so the study was included after discussions. The study by Pinkert *et al*[65] was conducted in Germany and Austria, with the latter not being included in the criteria; however, these two countries have similar healthcare systems and the working conditions in the dementia care and acute settings are similar, so this study was also deemed relevant in order to facilitate a comprehensive overview.[65 84] Additionally, the included studies applied different methodologies and theoretical approaches within qualitative research, which might have affected how the studies were interpreted and compared.

CONCLUSION

This qualitative systematic review identified HCP experiences of job satisfaction when providing PCC in healthcare European health care settings. Noteworthy is the experiences of a new professional role, with its own demands and initially disorientation and uncertainty. Vitally, the new role came with experiences of job satisfaction related to meaningfulness, deepened relationship between HCPs and patients, appreciation from patients and colleagues, and enhanced team collaboration. The findings are in line with theoretical assumptions of PCC not mainly aiming at job satisfaction, but improved relations from partnership and meaningfulness. An implication of our findings are the possibility for healthcare organisations to improve the initial phase of implementing PCC. This could be achieved by facilitating structures promoting team collaboration, and resources such as time, space and staffing. Future studies with a longitudinal follow-up approach with recurrent data collection can contribute with a deeper understanding of the mechanisms involved in the process towards increased job satisfaction when PCC is implemented. It can also contribute with causal inferences not possible from qualitative or cross-sectional studies.

Author contributions

All study authors; K.G (A1), C.v.D (A2), A.F (A3), M.A (A4), M.B (A5), and G.H (A6), were involved in planning and developing the design of the study. K.G created the eligibility criteria and search strategy, which all authors provided feedback on in discussions. K.G

For peer review only - http://bmjopen.bmj.com/site/about/guidelines.xhtml

developed the protocol and screening tool, which was reviewed by A.F, and G.H, who provided comments and suggestions for improvements. K.G and C.v.D screened titles, abstracts, and full-text studies for inclusion, which was discussed with A.F in a meeting. Extraction and synthesis of the data was conducted by K.G, supported by G.H through discussions, and then reviewed by all authors providing comments and suggestions for revisions. Both K.G and G.H conducted the quality assessments. K.G drafted the first version of the manuscript with constructive input from C.v.D, A.F., M.A., M.B, and G.H. Throughout the research process, the manuscript and its analyses, interpretations and conclusions were continuously discussed and critically assessed by all authors. The final version of the manuscript was approved by all authors.

Acknowledgements

We wish to thank Linda Hammarbäck at the Biomedical Library for contributing to the search strategy.

Funding

This work was supported by grants from the AFA Insurance (no.190030), an organisation owned by Sweden's labour market parties, and the University of Gothenburg Centre for Person-Centred Care (GPCC), Sweden. GPCC is funded by the Swedish Government's grant for Strategic Research Areas, Care Sciences (Application to Swedish Research Council no. 2009–1088). The funders have no role in the design of the study, data collection, analysis or interpretation.

Competing interests

None declared.

Patient consent for publication

Not required.

Ethics approval

No formal ethics approval was needed since this review is based on publicly available data from previously published studies.

Provenance and peer review

Not commissioned; externally peer reviewed.

For peer review only - http://bmjopen.bmj.com/site/about/guidelines.xhtml

Data availability statement

Data sharing not applicable as no datasets were generated and/or analysed for this study. This is a systematic review, and all the data used are taken from previously published material.

Supplemental material

For peer review only

For peer review only - http://bmjopen.bmj.com/site/about/guidelines.xhtml

REFERENCES

1. The European Agency for Safety and Health at Work. Third European Survey of Enterprises on New and Emerging Risks (ESENER 3) https://osha.europa.eu/en/publications/third-european-survey-enterprises-new-and-emerging-risks-esener-3/view, 2019.

2. The National Institute for Occupational Safety and Health. Healthcare Workers: Work Stress & Mental Health 2016 [Available from: https://www.cdc.gov/niosh/topics/healthcare/workstress.html accessed 21 February, 2022.

3. Harrison DA, Newman DA, Roth PL. How Important Are Job Attitudes? Meta-Analytic Comparisons of Integrative Behavioral Outcomes and Time Sequences. *Academy of Management journal* 2006;49(2):305-25. doi: 10.5465/AMJ.2006.20786077

4. Broetje S, Jenny GJ, Bauer GF. The Key Job Demands and Resources of Nursing Staff: An Integrative Review of Reviews. *Frontiers in Psychology* 2020;11 doi: 10.3389/fpsyg.2020.00084

5. Bigliardi B, Ivo Dormio A, Galati F, et al. The impact of organizational culture on the job satisfaction of knowledge workers. *VINE Very informal newsletter on library automation* 2012;42(1):36-51. doi: 10.1108/03055721211207752

6. Portoghese I, Galletta M, Coppola RC, et al. Burnout and workload among health care workers: the moderating role of job control. *Saf Health Work* 2014;5(3):152-57. doi: 10.1016/j.shaw.2014.05.004 [published Online First: 2014/06/07]

7. Halter M, Boiko O, Pelone F, et al. The determinants and consequences of adult nursing staff turnover: a systematic review of systematic reviews. *BMC Health Services Research* 2017;17(1):824. doi: 10.1186/s12913-017-2707-0

8. WHO. Spending on health in Europe: entering a new era. https://apps.who.int/iris/bitstream/handle/10665/340910/9789289055079-eng.pdf, 2021.

9. The European Commission. Recruitment and Retention of the Health Workforce in Europe. https://ec.europa.eu/health/other-pages/basic-page/recruitment-and-retention-health-workforce-europe-2015_en, 2015.

10. The European Commission. Occupational health and safety risks in the healthcare sector. https://op.europa.eu/en/publication-detail/-/publication/b29abb0a-f41e-4cb4-b787-4538ac5f0238/language-en/format-PDF/source-251761322, 2010.

For peer review only - http://bmjopen.bmj.com/site/about/guidelines.xhtml

11. Swedish Institute for Standards. Patient involvement in health care - Minimum requirements for person-centred care. https://www.sis.se/en/produkter/health-care-technology/general/health-care-services-in-general/ss-en-173982020/, 2020.

12. Ekman I. The GPCC. *European Heart Journal* 2018;39(7):495-96. doi: 10.1093/eurheartj/ehy029

13. Ekman I. Practising the ethics of person-centred care balancing ethical conviction and moral obligations. *Nurs Philos* 2022;23(3):e12382. doi: 10.1111/nup.12382 [published Online First: 20220225]

14. Britten N, Moore L, Lydahl D, et al. Elaboration of the Gothenburg model of person-centred care. *Health Expectations* 2017;20(3):407-18. doi: https://doi.org/10.1111/hex.12468

15. Ekman I, Swedberg K, Taft C, et al. Person-Centered Care — Ready for Prime Time. *European Journal of Cardiovascular Nursing* 2011;10(4):248-51. doi: 10.1016/j.ejcnurse.2011.06.008

16. Ekman N, Moons P, Taft C, et al. Observable indicators of person-centred care: an interview study with patients, relatives and professionals. *BMJ Open* 2022;12(4):e059308. doi: 10.1136/bmjopen-2021-059308

17. Epstein RM, Fiscella K, Lesser CS, et al. Why The Nation Needs A Policy Push On Patient-Centered Health Care. *Health Affairs* 2010;29(8):1489-95.

18. Vennedey V, Hower KI, Hillen H, et al. Patients' perspectives of facilitators and barriers to patient-centred care: insights from qualitative patient interviews. *BMJ Open* 2020;10(5):e033449. doi: 10.1136/bmjopen-2019-033449

19. Chenoweth L, Jeon Y-H, Stein-Parbury J, et al. PerCEN trial participant perspectives on the implementation and outcomes of person-centered dementia care and environments. *International Psychogeriatrics* 2015;27(12):2045-57. doi: 10.1017/S1041610215001350 [published Online First: 2015/08/26]

20. Mead N, Bower P. Patient-centredness: a conceptual framework and review of the empirical literature. *Soc Sci Med* 2000;51(7):1087-110. doi: 10.1016/s0277-9536(00)00098-8

21. Epstein RM. The science of patient-centered care. *J Fam Pract* 2000;49(9):805-7.

22. Beach MC, Inui T, Relationship-Centered Care Research N. Relationship-centered care. A constructive reframing. *J Gen Intern Med* 2006;21 Suppl 1(Suppl 1):S3-S8. doi: 10.1111/j.1525-1497.2006.00302.x

23. Kuo DZ, Houtrow AJ, Arango P, et al. Family-centered care: current applications and future directions in pediatric health care. *Matern Child Health J* 2012;16(2):297-305. doi: 10.1007/s10995-011-0751-7

24. Weiner SJ. Contextualizing medical decisions to individualize care: lessons from the qualitative sciences. *J Gen Intern Med* 2004;19(3):281-85. doi: 10.1111/j.1525-1497.2004.30261.x

25. Hughes JC, Bamford C, May C. Types of centredness in health care: themes and concepts. *Med Health Care Philos* 2008;11(4):455-63. doi: 10.1007/s11019-008-9131-5 [published Online First: 20080409]

For peer review only - http://bmjopen.bmj.com/site/about/guidelines.xhtml

26. Moore L, Britten N, Lydahl D, et al. Barriers and facilitators to the implementation of person-centred care in different healthcare contexts. *Scand J Caring Sci* 2017;31(4):662-73. doi: 10.1111/scs.12376 [published Online First: 2016/11/08]

27. Aiken LH, Sloane DM, Bruyneel L, et al. Nurses' reports of working conditions and hospital quality of care in 12 countries in Europe. *International Journal of Nursing Studies* 2013;50(2):143-53. doi: https://doi.org/10.1016/j.ijnurstu.2012.11.009

28. van Diepen C, Fors A, Ekman I, et al. Associations between person-centred care and job strain, stress of conscience, and intent to leave among hospital personnel. *Journal of Clinical Nursing* 2022;31(5-6):612-22. doi: https://doi.org/10.1111/jocn.15919

29. Britten N, Ekman I, Naldemirci Ö, et al. Learning from Gothenburg model of person centred healthcare. *BMJ* 2020;370:m2738. doi: 10.1136/bmj.m2738

30. Rajamohan S, Porock D, Chang Y-P. Understanding the Relationship Between Staff and Job Satisfaction, Stress, Turnover, and Staff Outcomes in the Person-Centered Care Nursing Home Arena. *Journal of Nursing Scholarship* 2019;51(5):560-68. doi: https://doi.org/10.1111/jnu.12488

31. van den Pol-Grevelink A, Jukema JS, Smits CHM. Person-centred care and job satisfaction of caregivers in nursing homes: a systematic review of the impact of different forms of person-centred care on various dimensions of job satisfaction. *International Journal of Geriatric Psychiatry* 2012;27(3):219-29. doi: https://doi.org/10.1002/gps.2719

32. Güney S, Karadağ A, El-Masri M. Perceptions and experiences of person-centered care among nurses and nurse aides in long term residential care facilities: A systematic review of qualitative studies. *Geriatric Nursing* 2021;42(4):816-24. doi: https://doi.org/10.1016/j.gerinurse.2021.04.005

33. van Diepen C, Fors A, Ekman I, et al. Association between person-centred care and healthcare providers' job satisfaction and work-related health: a scoping review. *BMJ Open* 2020;10(12):e042658. doi: 10.1136/bmjopen-2020-042658 [published Online First: 2020/12/10]

34. Barbosa A, Sousa L, Nolan M, et al. Effects of Person-Centered Care Approaches to Dementia Care on Staff: A Systematic Review. *Am J Alzheimers Dis Other Demen* 2015;30(8):713-22. doi: 10.1177/1533317513520213 [published Online First: 2014/01/23]

35. Brownie S, Nancarrow S. Effects of person-centered care on residents and staff in aged-care facilities: a systematic review. *Clin Interv Aging* 2013;8:1-10. doi: 10.2147/cia.S38589 [published Online First: 20130103]

36. Shamseer L, Moher D, Clarke M, et al. Preferred reporting items for systematic review and meta-analysis protocols (PRISMA-P) 2015: elaboration and explanation. *BMJ : British Medical Journal* 2015;349:g7647. doi: 10.1136/bmj.g7647

37. Thomas J, Harden A. Methods for the thematic synthesis of qualitative research in systematic reviews. *BMC Medical Research Methodology* 2008;8(1):45. doi: 10.1186/1471-2288-8-45

38. Tong A, Flemming K, McInnes E, et al. Enhancing transparency in reporting the synthesis of qualitative research: ENTREQ. *BMC Medical Research Methodology* 2012;12(1):181. doi: 10.1186/1471-2288-12-181

For peer review only - http://bmjopen.bmj.com/site/about/guidelines.xhtml

39. Bettany-Saltikov J. How to do a systematic literature review in nursing : a step-by-step guide. 2. ed. ed: London : McGraw-Hill Education/Open University Press 2016.

40. Rosengren K, Brannefors P, Carlström E. Adoption of the concept of person-centred care into discourse in Europe: a systematic literature review. *Journal Of Health Organization And Management, 2021, Vol 35, Iss 9, pp 265-280* 2021;35(9):265-80. doi: 10.1108/JHOM-01-2021-0008

41. National Library of Medicine. Medical Subject Headings 2022 [Available from: https://www.nlm.nih.gov/mesh/meshhome.html accessed 11 February, 2022.

42. Sayers A. Tips and tricks in performing a systematic review--chapter 4. *Br J Gen Pract* 2008;58(547):136-36. doi: 10.3399/bjgp08X277168

43. Polanin JR, Pigott TD, Espelage DL, et al. Best practice guidelines for abstract screening large-evidence systematic reviews and meta-analyses. *Research Synthesis Methods* 2019;10(3):330-42. doi: https://doi.org/10.1002/jrsm.1354

44. Bramer WM, Giustini D, de Jonge GB, et al. De-duplication of database search results for systematic reviews in EndNote. *J Med Libr Assoc* 2016;104(3):240-43. doi: 10.3163/1536-5050.104.3.014

45. Ouzzani M, Hammady H, Fedorowicz Z, et al. Rayyan-a web and mobile app for systematic reviews. *Syst Rev* 2016;5(1):210. doi: 10.1186/s13643-016-0384-4 [published Online First: 2016/12/07]

46. Page MJ, McKenzie JE, Bossuyt PM, et al. The PRISMA 2020 statement: an updated guideline for reporting systematic reviews. *BMJ* 2021;372:n71. doi: 10.1136/bmj.n71

47. SBU. Appraisal of Studies with Qualitative Methodology. https://www.sbu.se/globalassets/ebm/bedomning_studier_kvalitativ_metodik.pdf, 2020.

48. SBU. Guidance for Assessment of Studies with Qualitative Methodology. https://www.sbu.se/globalassets/ebm/vagledning_granskning_studier_kvalitativ_metodik.pdf, 2020.

49. SBU. SBU:s metodbok. https://www.sbu.se/sv/metod/sbus-metodbok/?pub=48286#48490, 2020.

50. Boström E, Isaksson U, Lundman B, et al. Patient-centred care in type 2 diabetes - an altered professional role for diabetes specialist nurses. *Scand J Caring Sci* 2014;28(4):675-82. doi: 10.1111/scs.12092

51. Fridberg H, Wallin L, Tistad M. The innovation characteristics of person-centred care as perceived by healthcare professionals: an interview study employing a deductive-inductive content analysis guided by the consolidated framework for implementation research. *BMC Health Services Research* 2021;21(1):904. doi: 10.1186/s12913-021-06942-y

52. Karlsson AC, Gunningberg L, Bäckström J, et al. Registered nurses' perspectives of work satisfaction, patient safety and intention to stay - A double-edged sword. *J Nurs Manag* 2019;27(7):1359-65. doi: 10.1111/jonm.12816

53. Nilsson A, Edvardsson D, Rushton C. Nurses' descriptions of person-centred care for older people in an acute medical ward-On the individual, team and organisational levels'. *J Clin Nurs* 2019;28(7):1251-59. doi: https://doi.org/10.1111/jocn.14738

For peer review only - http://bmjopen.bmj.com/site/about/guidelines.xhtml

54. Kjörnsberg A, Karlsson L, Babra A, et al. Registered nurses' opinions about patient focused care. *Australian Journal of Advanced Nursing* 2010;28(1):35-44.

55. Sjöberg V, Forsner M. Shifting roles: physiotherapists' perception of person-centered care during a pre-implementation phase in the acute hospital setting - A phenomenographic study. *Physiother Theory Pract* 2020:1-11. doi: 10.1080/09593985.2020.1809042

56. Öhman A, Keisu BI, Enberg B. Team social cohesion, professionalism, and patient-centeredness: Gendered care work, with special reference to elderly care - a mixed methods study. *BMC Health Serv Res* 2017;17(1):381. doi: https://doi.org/10.1186/s12913-017-2326-9

57. Kadri A, Rapaport P, Livingston G, et al. Care workers, the unacknowledged persons in person-centred care: A secondary qualitative analysis of UK care home staff interviews. *PLoS One* 2018;13(7):e0200031. doi: 10.1371/journal.pone.0200031

58. Ross H, Tod AM, Clarke A. Understanding and achieving person-centred care: the nurse perspective. *J Clin Nurs* 2015;24(9):1223-33. doi: 10.1111/jocn.12662

59. Walker W, Deacon K. Nurses' experiences of caring for the suddenly bereaved in adult acute and critical care settings, and the provision of person-centred care: A qualitative study. *Intensive Crit Care Nurs* 2016;33:39-47. doi: 10.1016/j.iccn.2015.12.005

60. Kirkley C, Bamford C, Poole M, et al. The impact of organisational culture on the delivery of person-centred care in services providing respite care and short breaks for people with dementia. *Health Soc Care Community* 2011;19(4):438-48. doi: 10.1111/j.1365-2524.2011.00998.x

61. Boersma P, Dröes RM, Lissenberg-Witte BI, et al. Does working with the Veder Contact Method influence the job satisfaction of caregivers? A non-randomized controlled trial in nursing homes for people with dementia. *Int Psychogeriatr* 2017;29(12):2017-32. doi: 10.1017/S1041610217001582

62. Uittenbroek RJ, van der Mei SF, Slotman K, et al. Experiences of case managers in providing person-centered and integrated care based on the Chronic Care Model: A qualitative study on embrace. *PLoS One* 2018;13(11):e0207109. doi: 10.1371/journal.pone.0207109

63. Coyne I. Families and health-care professionals' perspectives and expectations of family-centred care: hidden expectations and unclear roles. *Health Expect* 2015;18(5):796-808. doi: 10.1111/hex.12104

64. Barbosa A, Nolan M, Sousa L, et al. Supporting direct care workers in dementia care: effects of a psychoeducational intervention. *Am J Alzheimers Dis Other Demen* 2015;30(2):130-8. doi: https://doi.org/10.1177/1533317514550331

65. Pinkert C, Faul E, Saxer S, et al. Experiences of nurses with the care of patients with dementia in acute hospitals: A secondary analysis. *J Clin Nurs* 2018;27(1):162-72. doi: 10.1111/jocn.13864

66. Vassbø TK, Kirkevold M, Edvardsson D, et al. The meaning of working in a person-centred way in nursing homes: a phenomenological-hermeneutical study. *BMC Nurs* 2019;18:45. doi: https://doi.org/10.1186/s12912-019-0372-9

67. Rapaport P, Livingston G, Hamilton O, et al. How do care home staff understand, manage and respond to agitation in people with dementia? A qualitative study. *BMJ Open* 2018;8(6):e022260. doi: 10.1136/bmjopen-2018-022260

For peer review only - http://bmjopen.bmj.com/site/about/guidelines.xhtml

68. Allerby K, Goulding A, Ali L, et al. Increasing person-centeredness in psychosis inpatient care: staff experiences from the Person-Centered Psychosis Care (PCPC) project. *BMC Health Services Research* 2022;22(1):596. doi: 10.1186/s12913-022-08008-z

69. Petersson Å, Hellström A, Assarsson J, et al. Following a standardised pathway: Healthcare professionals' perspectives on person-centred care within ERAS for patients with colorectal cancer. *Journal of Clinical Nursing* 2022;n/a(n/a) doi: https://doi.org/10.1111/jocn.16562

70. Boström E, Ali L, Fors A, et al. Registered nurses' experiences of communication with patients when practising person-centred care over the phone: a qualitative interview study. *BMC Nurs* 2020;19:54. doi: https://doi.org/10.1186/s12912-020-00448-4

71. Santana MJ, Manalili K, Jolley RJ, et al. How to practice person-centred care: A conceptual framework. *Health Expectations* 2018;21(2):429-40. doi: https://doi.org/10.1111/hex.12640

72. Summer Meranius M, Holmström IK, Håkansson J, et al. Paradoxes of person-centred care: A discussion paper. *Nursing Open* 2020;7(5):1321-29. doi: https://doi.org/10.1002/nop2.520

73. Juthberg C, Sundin K. Registered nurses' and nurse assistants' lived experience of troubled conscience in their work in elderly care--a phenomenological hermeneutic study. *Int J Nurs Stud* 2010;47(1):20-9. doi: 10.1016/j.ijnurstu.2009.06.001 [published Online First: 20090701]

74. Backman A, Sjögren K, Lövheim H, et al. The influence of nursing home managers' leadership on person-centred care and stress of conscience: A cross-sectional study. *BMC Nursing* 2021;20(1):200. doi: 10.1186/s12912-021-00718-9

75. WHO. WHO global strategy on integrated people-centred health services 2016-2026. https://interprofessional.global/wp-content/uploads/2019/11/WHO-2015-Global-strategy-on-integrated-people-centred-health-services-2016-2026.pdf, 2016.

76. Montgomery A, Spânu F, Băban A, et al. Job demands, burnout, and engagement among nurses: A multi-level analysis of ORCAB data investigating the moderating effect of teamwork. *Burnout Research* 2015;2(2):71-79. doi: https://doi.org/10.1016/j.burn.2015.06.001

77. Keyko K, Cummings GG, Yonge O, et al. Work engagement in professional nursing practice: A systematic review. *Int J Nurs Stud* 2016;61:142-64. doi: 10.1016/j.ijnurstu.2016.06.003 [published Online First: 2016/06/29]

78. Hower KI, Vennedey V, Hillen HA, et al. Implementation of patient-centred care: which organisational determinants matter from decision maker's perspective? Results from a qualitative interview study across various health and social care organisations. *BMJ Open* 2019;9(4):e027591. doi: 10.1136/bmjopen-2018-027591

79. Snyder H. Literature review as a research methodology: An overview and guidelines. *Journal of Business Research* 2019;104:333-39. doi: https://doi.org/10.1016/j.jbusres.2019.07.039

80. Booth A, Clarke M, Ghersi D, et al. An international registry of systematic-review protocols. *The Lancet* 2011;377(9760):108-09. doi: https://doi.org/10.1016/S0140-6736(10)60903-8

For peer review only - http://bmjopen.bmj.com/site/about/guidelines.xhtml

81. Xiao Y, Watson M. Guidance on Conducting a Systematic Literature Review. *Journal of Planning Education and Research* 2017;39(1):93-112. doi: 10.1177/0739456X17723971

82. Butler A, Hall H, Copnell B. A Guide to Writing a Qualitative Systematic Review Protocol to Enhance Evidence-Based Practice in Nursing and Health Care. *Worldviews on Evidence-Based Nursing* 2016;13(3):241-49. doi: https://doi.org/10.1111/wvn.12134

83. Noyes J, Booth A, Cargo M, et al. Qualitative evidence. Cochrane Handbook for Systematic Reviews of Interventions2019:525-45.

84. The European Parliament. Health Care Systems in the EU: A Comparative Study. https://www.europarl.europa.eu/workingpapers/saco/pdf/101_en.pdf, 1998.

For peer review only

For peer review only - http://bmjopen.bmj.com/site/about/guidelines.xhtml

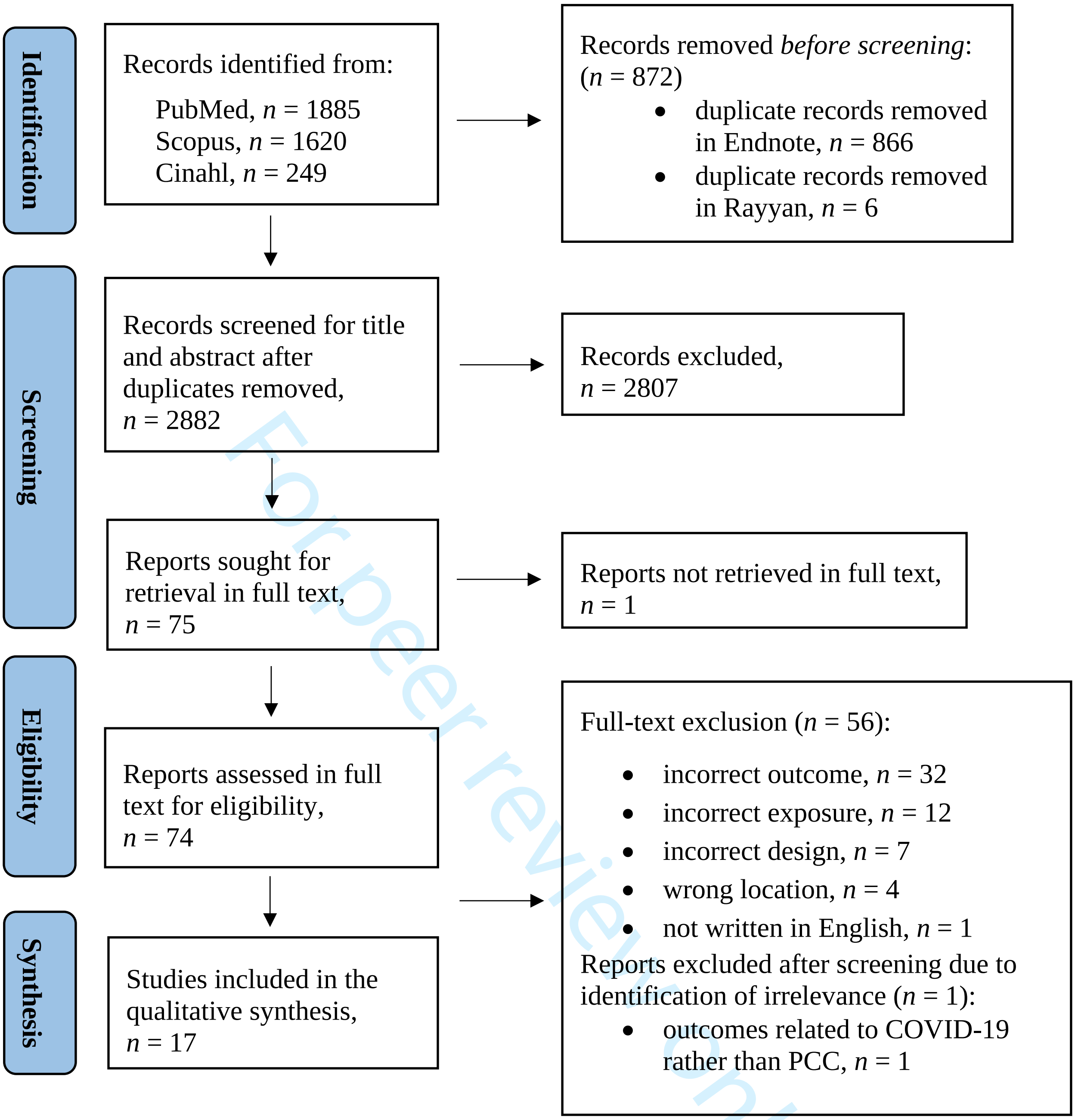

For peer review only

Online Supplementary File 1 – ENTREQ

No	Item	Guide and description	Reported on page #
**1**	Aim	State the research question the synthesis addresses.	5
**2**	Synthesis methodology	Identify the synthesis methodology or theoretical framework which underpins the synthesis, and describe the rationale for choice of methodology *(e.g. meta-ethnography, thematic synthesis, critical interpretive synthesis, grounded theory synthesis, realist synthesis, meta-aggregation, meta-study, framework synthesis).*	9
**3**	Approach to searching	Indicate whether the search was pre-planned (*comprehensive search strategies to seek all available studies)* or iterative (*to seek all available concepts until they theoretical saturation is achieved)*.	6
**4**	Inclusion criteria	Specify the inclusion/exclusion criteria *(e.g. in terms of population, language, year limits, type of publication, study type).*	6
**5**	Data sources	Describe the information sources used (e.g. *electronic databases (MEDLINE, EMBASE, CINAHL, psycINFO, Econlit), grey literature databases (digital thesis, policy reports), relevant organisational websites, experts, information specialists, generic web searches (Google Scholar) hand searching, reference lists)* and when the searches conducted; provide the rationale for using the data sources.	5-6
**6**	Electronic Search strategy	Describe the literature search *(e.g. provide electronic search strategies with population terms, clinical or health topic terms, experiential or social phenomena related terms, filters for qualitative research, and search limits).*	6-7
**7**	Study screening methods	Describe the process of study screening and sifting *(e.g. title, abstract and full text review, number of independent reviewers who screened studies).*	4
**8**	Study characteristics	Present the characteristics of the included studies *(e.g. year of publication, country, population, number of participants, data collection, methodology, analysis, research questions).*	7-8
**9**	Study selection results	Identify the number of studies screened and provide reasons for study exclusion *(e,g, for comprehensive searching, provide numbers of studies screened and reasons for exclusion indicated in a figure/flowchart; for iterative searching describe reasons for study exclusion and*	7-8

For peer review only

No	Item	Guide and description	Reported on page #
		inclusion based on modifications t the research question and/or contribution to theory development).	
**10**	Rationale for appraisal	Describe the rationale and approach used to appraise the included studies or selected findings *(e.g. assessment of conduct (validity and robustness), assessment of reporting (transparency), assessment of content and utility of the findings).*	8-9
**11**	Appraisal items	State the tools, frameworks and criteria used to appraise the studies or selected findings *(e.g. Existing tools: CASP, QARI, COREQ, Mays and Pope* [25]*; reviewer developed tools; describe the domains assessed: research team, study design, data analysis and interpretations, reporting).*	8
**12**	Appraisal process	Indicate whether the appraisal was conducted independently by more than one reviewer and if consensus was required.	8
**13**	Appraisal results	Present results of the quality assessment and indicate which articles, if any, were weighted/excluded based on the assessment and give the rationale.	8-9
**14**	Data extraction	Indicate which sections of the primary studies were analysed and how were the data extracted from the primary studies? *(e.g. all text under the headings 'results /conclusions' were extracted electronically and entered into a computer software).*	9
**15**	Software	State the computer software used, if any.	Endnote, NVivo, Rayyan
**16**	Number of reviewers	Identify who was involved in coding and analysis.	9
**17**	Coding	Describe the process for coding of data *(e.g. line by line coding to search for concepts).*	9
**18**	Study comparison	Describe how were comparisons made within and across studies *(e.g. subsequent studies were coded into pre-existing concepts, and new concepts were created when deemed necessary).*	9
**19**	Derivation of themes	Explain whether the process of deriving the themes or constructs was inductive or deductive.	9
**20**	Quotations	Provide quotations from the primary studies to illustrate themes/constructs, and identify whether the quotations were participant quotations of the author's interpretation.	N/A

For peer review only

No	Item	Guide and description	Reported on page #
**21**	Synthesis output	Present rich, compelling and useful results that go beyond a summary of the primary studies (e.g. *new interpretation, models of evidence, conceptual models, analytical framework, development of a new theory or construct).*	20-23

For peer review only

Online Supplementary File 2 – Search strategy

Search strategy				
**Population**	**Exposure**	**Outcome**	**Countries**	**Study design**
“health personnel” “care personnel” “health care provider*” “care provider*” “health care worker*” “health care staff” “caregiver*” “care assistant*” “care aides” “nurse*” “nursing*” “physician*” “physiotherapist*” “physical therapist*” “psychologist*” “social worker*” “health care professional*” “nutritionist*” “case manager*” “medical staff” “general practitioner*”	“person centered” “person centeredness” “client centered” “patient centered” **“patient-centered care”** “relationship centered” “family centered” “patient focused” “person focused” “individualized care” “personalized care” “person-directed care” “individual centered”	**“perception*”** “experience*” “perspective*” **“attitude*”** “stress of conscience” **“job satisfaction” “absenteeism” “presenteeism” “occupational stress” “occupational health” “personnel turnover*” “sick leave” “stress, psychological”** “psychological stress” **“dyssomnia*”** “sleep disorder*” “sleep disturbance*” “moral stress” **“attitude of health personnel”**	“Sweden” “Swedish” “Norway” “Norwegian” “Finland” “Finnish” “Denmark” “Danish” “Iceland” “Icelandic” “United kingdom” “UK” “British” “North Ireland” “North Irish” “Wales” “Welsh” “Scotland” “Scottish” “Ireland” “Irish” “the Netherlands” “Dutch” “Germany” “German” “Spain” “Spanish” “Italy” “Italian” “Belgium” “Belgian” “Portugal” “Portuguese” “Malta” “Maltese” “Estonia” “Estonian” “Slovenia” “Slovenian” “Czech republic” “Czech” “France” “French” “Poland” “polish” “Hungary”	**“qualitative research”** “qualitative stud*” **“focus group*” “grounded theory”** “mixed-method” “ethnography” “phenomenology*” **“interviews as topic”** “interview*” **“hermeneutic*”**

“Hungarian” “Greece” “Greek” “Latvia” “Latvian” “Serbia” “Serbian”

For peer review only

PubMed 1885

Scopus 1620

Cinahl 249

PubMed searched 21 December 2021

(Sweden OR Swedish OR Norway OR Norwegian OR Finland OR Finnish OR Denmark OR Danish OR Iceland OR Icelandic OR “United Kingdom” OR UK OR British OR North Ireland OR North Irish OR Wales OR Welsh OR Scotland OR Scottish OR Ireland OR Irish OR The Netherlands OR Netherlands OR Dutch OR Germany OR German OR Spain OR Spanish OR Italy OR Italian OR Belgium OR Belgian OR Portugal OR Portuguese OR Malta OR Maltese OR Estonia OR Estonian OR Slovenia OR Slovenian OR Czech Republic OR Czech OR France OR French OR Poland OR Polish OR Hungary OR Hungarian OR Greece OR Greek OR Latvia OR Latvian OR Serbia OR Serbian)

AND

(“health personnel”[MeSH] OR “health personnel”[tiab] OR “care personnel”[tiab] OR “health care provider”[tiab] OR “health care providers”[tiab] OR “care provider”[tiab] OR “care providers”[tiab] OR “health care worker”[tiab] OR “health care staff”[tiab] OR caregiver[tiab] OR caregivers[tiab] OR caregivers[mesh] OR “healthcare worker”[tiab] OR “healthcare workers”[tiab] OR “care assistant”[tiab] OR “care aides”[tiab] OR nurses[mesh] OR nurse[tiab] OR nurses[tiab] OR nursing[tiab] OR physicians[mesh] OR physicians[tiab] OR physician[tiab] OR physiotherapists[tiab] OR “physical therapists”[mesh] OR “physical therapists”[tiab] OR psychologist[tiab] OR “social workers”[mesh] OR “social workers”[tiab] OR “social worker”[tiab] OR “Health care professionals”[tiab] OR “health care professional”[tiab] OR “nutritionist”[tiab] OR nutritionists[mesh] OR “case managers” [tiab] OR “Medical staff”[mesh] OR “Medical staff”[tiab] OR “general practitioners”[tiab] OR “General Practitioners”[mesh])

AND

(“Person centered”[tiab] OR “person centred”[tiab] OR “person-centred”[tiab] OR “person centeredness”[tiab] OR “personcenteredness”[tiab] OR “person centredness”[tiab] OR “client centered”[tiab] OR “client centred”[tiab] OR “patient centered”[tiab] OR “patient centred”[tiab] OR “Patient-Centered Care”[Mesh] OR “relationship centered”[tiab] OR “relationship centred”[tiab] OR “family centred”[tiab] OR “patient focused”[tiab] OR “person

For peer review only

focused”[tiab] OR “individualized care” [tiab] OR “personalized care” [tiab] OR “person-directed care” [tiab] OR “individual centered”[tiab])

AND

(perception[mesh] OR perception[tiab] OR experience[tiab] OR experiences[tiab] OR perspective[tiab] OR attitude[mesh] OR attitude[tiab] OR attitudes[tiab] OR “stress of conscience”[tiab] OR “Job Satisfaction”[Mesh] OR “job satisfaction”[tiab] OR “Absenteeism”[Mesh] OR “absenteeism”[tiab] OR “presenteeism”[Mesh] OR “presenteeism”[tiab] OR “Occupational Stress”[Mesh] OR “occupational stress”[tiab] OR “Personnel Turnover”[Mesh] OR “personnel turnover”[tiab] OR “Sick Leave”[Mesh] OR “sick leave”[tiab] OR “Stress, Psychological”[Mesh] OR “psychological stress”[tiab] OR “Dyssomnias”[Mesh] OR “sleep disorder”[tiab] OR “sleep disorders”[tiab] OR “sleep disturbances”[tiab] OR “occupational health”[Mesh] OR “occupational health”[tiab] OR “moral stress”[tiab] OR “Attitude of Health Personnel”[mesh])

AND

(“qualitative research”[mesh] OR “qualitative research”[tiab] OR “qualitative study”[tiab] OR “qualitative studies”[tiab] OR “focus groups”[mesh] OR “focus groups”[tiab] OR “focus group”[tiab] OR “Grounded theory”[mesh] OR “grounded theory”[tiab] OR “mixed-method”[tiab] OR ethnography[tiab] OR phenomenology*[tiab] OR interview*[tiab] OR “interviews as topic”[mesh] OR Hermeneutics[mesh] OR hermeneutic*[tiab])

Scopus searched 21 December 2021

(sweden OR swedish OR norway OR norwegian OR finland OR finnish OR denmark OR danish OR iceland OR icelandic OR “United Kingdom” OR uk OR british OR “North Ireland” OR “North Irish” OR wales OR welsh OR scotland OR scottish OR ireland OR irish OR the AND netherlands OR netherlands OR dutch OR germany OR german OR spain OR spanish OR italy OR italian OR belgium OR belgian OR portugal OR portuguese OR malta OR maltese OR estonia OR estonian OR slovenia OR slovenian OR “Czech Republic” OR czech OR france OR french OR poland OR polish OR hungary OR hungarian OR greece OR greek OR latvia OR latvian OR serbia OR serbian) AND TITLE-ABS-KEY (“health personnel” OR “care personnel” OR “health care provider*” OR “care provider*” OR “health care worker*” OR “health care staff” OR caregiver* OR “healthcare worker*” OR “care assistant*” OR “care

For peer review only

aides" OR nurse* OR nursing OR physician* OR physiotherapist* OR "physical therapist*" OR psychologist* OR "social worker*" OR "Health care professional*" OR "nutritionist*" OR "case manager*" OR "Medical staff" OR "general practitioner*") AND TITLE-ABS-KEY ("Person centered" OR "person centred" OR "person centeredness" OR "personcenteredness" OR "person centredness" OR "client centered" OR "client centred" OR "patient centered" OR "patient centred" OR "Patient-Centered Care" OR "relationship centered" OR "relationship centred" OR "family centred" OR "patient focused" OR "person focused" OR "individualized care" OR "personalized care" OR "person-directed care" OR "individual centered") AND TITLE-ABS-KEY (perception* OR experience* OR perspective* OR attitude* OR "stress of conscience" OR "Job Satisfaction" OR absenteeism OR presenteeism OR "Occupational Stress" OR "Personnel Turnover*" OR "Sick Leave" OR "psychological stress" OR dyssomnia* OR "sleep disorder*" OR "sleep disturbance*" OR "occupational health" OR "moral stress") AND TITLE-ABS-KEY ("qualitative research" OR "qualitative stud*" OR "focus group*" OR "Grounded theory" OR "mixed-method" OR ethnography OR phenomenology OR interview* OR hermeneutic*) AND (LIMIT-TO (PUBYEAR , 2022) OR LIMIT-TO (PUBYEAR , 2021) OR LIMIT-TO (PUBYEAR , 2020) OR LIMIT-TO (PUBYEAR , 2019) OR LIMIT-TO (PUBYEAR , 2018) OR LIMIT-TO (PUBYEAR , 2017) OR LIMIT-TO (PUBYEAR , 2016) OR LIMIT-TO (PUBYEAR , 2015) OR LIMIT-TO (PUBYEAR , 2014) OR LIMIT-TO (PUBYEAR , 2013) OR LIMIT-TO (PUBYEAR , 2012) OR LIMIT-TO (PUBYEAR , 2011) OR LIMIT-TO (PUBYEAR , 2010))

Cinahl searched 21 December 2021

(Sweden OR Swedish OR Norway OR Norwegian OR Finland OR Finnish OR Denmark OR Danish OR Iceland OR Icelandic OR "United Kingdom" OR UK OR British OR "North Ireland" OR "North Irish" OR Wales OR Welsh OR Scotland OR Scottish OR Ireland OR Irish OR The Netherlands OR Netherlands OR Dutch OR Germany OR German OR Spain OR Spanish OR Italy OR Italian OR Belgium OR Belgian OR Portugal OR Portuguese OR Malta OR Maltese OR Estonia OR Estonian OR Slovenia OR Slovenian OR "Czech Republic" OR Czech OR France OR French OR Poland OR Polish OR Hungary OR Hungarian OR Greece OR Greek OR Latvia OR Latvian OR Serbia OR Serbian)

AND

For peer review only

((MH “health personnel+”) OR (TI “health personnel” OR AB “health personnel”) OR (TI “care personnel” OR AB “care personnel”) OR (TI “health care provider” OR AB “health care provider”) OR (TI “health care providers” OR AB “health care providers”) OR (TI “care provider” OR AB “care provider”) OR (TI “care providers” OR AB “care providers”) OR (TI “health care staff” OR AB “health care staff”) OR (TI caregiver OR AB caregiver) OR (TI caregivers OR AB caregivers) OR (MH caregivers+) OR (TI “healthcare worker” OR AB “healthcare worker”) OR (TI “healthcare workers” OR AB “healthcare workers”) OR (TI “care assistant” OR AB “care assistant”) OR (TI “care aides” OR AB “care aides”) OR (MH nurses+) OR (TI nursing OR AB nursing) OR (TI nurse OR AB nurse) OR (TI nurses OR AB nurses) OR (MH physicians+) OR (TI physicians OR AB physicians) OR (TI physician OR AB physician) OR (TI physiotherapists OR AB physiotherapists) OR (MH “physical therapists+”) OR (TI “physical therapists” OR AB “physical therapists”) OR (TI psychologist OR AB psychologist) OR (MH “social workers+”) OR (TI “social workers” OR AB “social workers”) OR (TI “social worker” OR AB “social worker”) OR (TI “Health care professionals” OR AB “Health care professionals”) OR (TI “health care professional” OR AB “health care professional”) OR (TI nutritionist OR AB nutritionist) OR (TI “case managers” OR AB “case managers”) OR (MH “Medical staff+”) OR (TI “Medical staff” OR AB “Medical staff”) OR (TI “general practitioners” OR AB “general practitioners”) OR (MH “Physicians, Family+”))

AND

((TI “Person centered” OR AB “Person centered”) OR (TI “person centred” OR AB “person centred”) OR (TI “person centeredness” OR AB “person centeredness”) OR (TI personcenteredness OR AB personcenteredness) OR (TI “person centredness” OR AB “person centredness”) OR (TI “client centered” OR AB “client centered”) OR (TI “client centred” OR AB “client centred”) OR (TI “patient centered” OR AB “patient centered”) OR (TI “patient centred” OR AB “patient centred”) OR (MH “Patient Centered Care”) OR (TI “relationship centered” OR AB “relationship centered”) OR (TI “relationship centred” OR AB “relationship centred”) OR (TI “family centred” OR AB “family centred”) OR (TI “patient focused” OR AB “patient focused”) OR (TI “person focused” OR AB “person focused”) OR (TI “individualized care” OR AB “individualized care”) OR (TI “personalized care” OR AB “personalized care”) OR (TI “person-directed care” OR AB “person-directed care”) OR (TI “individual centered” OR AB “individual centered”)) AND ((MH perception+) OR (TI perception OR AB perception) OR (TI experience OR AB experience) OR (TI

For peer review only

experiences OR AB experiences) OR (TI perspective OR AB perspective) OR (MH attitude+) OR (TI attitude OR AB attitude) OR (TI attitudes OR AB attitudes) OR (TI “stress of conscience” OR AB “stress of conscience”) OR (MH “Job Satisfaction+”) OR (TI “job satisfaction” OR AB “job satisfaction”) OR (MH Absenteeism+) OR (TI absenteeism OR AB absenteeism) OR (MH presenteeism+) OR (TI presenteeism OR AB presenteeism) OR (MH “Stress, Occupational+”) OR (TI “occupational stress” OR AB “occupational stress”) OR (MH “Personnel Turnover+”) OR (TI “personnel turnover” OR AB “personnel turnover”) OR (MH “Sick Leave+”) OR (TI “sick leave” OR AB “sick leave”) OR (MH “Stress, Psychological+”) OR (TI “psychological stress” OR AB “psychological stress”) OR (MH Dyssomnias+) OR (TI “sleep disorder” OR AB “sleep disorder”) OR (TI “sleep disorders” OR AB “sleep disorders”) OR (TI “sleep disturbances” OR AB “sleep disturbances”) OR (MH “occupational health+”) OR (TI “occupational health” OR AB “occupational health”) OR (TI “moral stress” OR AB “moral stress”) OR (MH “Attitude of Health Personnel+”))

AND

((MH “qualitative studies+”) OR (TI “qualitative research” OR AB “qualitative research”) OR (TI “qualitative study” OR AB “qualitative study”) OR (TI “qualitative studies” OR AB “qualitative studies”) OR (MH “focus groups+”) OR (TI “focus groups” OR AB “focus groups”) OR (TI “focus group” OR AB “focus group”) OR (MH “Grounded theory+”) OR (TI “grounded theory” OR AB “grounded theory”) OR (TI mixed-method OR AB mixed-method) OR (TI ethnography OR AB ethnography) OR (TI phenomenology* OR AB phenomenology*) OR (TI interview* OR AB interview*) OR (MH “interviews+”) OR (MH “Phenomenology”) OR (TI hermeneutic* OR AB hermeneutic*))

For peer review only

Online Supplementary File 3 – Screening Tool

Screening Tool

Citation, Title, and Abstract Screening

1. Does the **citation** indicate publication on or after 2010?

    a. Yes or Unsure/Unclear: continue screening

    b. No: stop screening

2. Does the **title or abstract** use English?

    a. Yes or Unsure/Unclear: continue screening

    b. No: stop screening

3. Does the **title or abstract** indicate that the study uses a qualitative design?

    a. Yes or Unsure/Unclear: continue screening

    b. No: stop screening

Keywords: qualitative, interview, focus group, grounded-theory, mixed-method, ethnography, phenomenology, hermeneutic.

4. Does the **title or abstract** indicate that the study concerns PCC?

    a. Yes or Unsure/Unclear: continue screening

    b. No: stop screening

Note: This also includes the different definitions of PCC, e.g., Patient-Centred Care, Relationship-Centred Care, Client-Centred Care etc. (See block 3 in Search Strategy).

5. Does the **title or abstract** indicate that the study concerns HCPs perspectives/experiences of PCC?

    a. Yes or Unsure/Unclear: continue screening

    b. No: stop screening

6. Does the **title or abstract** indicate that the research was conducted in one of the countries from the inclusion criteria?

a. Yes or Unsure/Unclear: continue screening

b. No: stop screening

Countries: Sweden, Norway, Finland, Denmark, Iceland, United Kingdom (England, Scotland, Wales, North Ireland) Ireland, The Netherlands, Germany, Spain, Italy, Belgium, Portugal, Malta, Estonia, Slovenia, Czech Republic, France, Poland, Hungary, Greece, Latvia, Serbia.

7. Does the **title or abstract** indicate that job satisfaction/occupational health in relation to PCC was studied?

a. Yes or Unsure/Unclear: continue screening

b. No: stop screening

Keywords: satisfaction, job satisfaction, job strain, stress, moral stress, stress of conscience, demand, occupational health

Decision: **Should this article be included for full-text read?**

a. **Yes**, all 7 screening questions answered Yes or Unsure/Unclear

b. **No**, at least one answers definitely "No"

review only

Online Supplementary File 4 – SBU Checklist

Bedömning av studier med kvalitativ metodik

UPPDATERAD 2020-10-06

Författare: ________________ År: __________

Granskare: ____________________________

Sammanvägd bedömning av metodologiska brister:
Obetydliga eller mindre ☐<br>Måttliga ☐
Stora brister, studien ingår inte i syntesen ☐

Kommentarer:

only

1. Överensstämmelse mellan filosofisk hållning/teori och urval och metodik i studien[1]

Vilken teori eller filosofisk hållning utgick författarna från?

	Ja	Nej	Oklart
Hänger syfte och fråga ihop med teori/filosofisk hållning?	☐	☐	☐

Kommentarer:

2. Deltagare

Hur gjordes urvalet?

**Stödfrågor för bedömning av brister i urvalsförfarandet:**	Ja	Nej	Oklart
Är urvalet lämpligt för att besvara frågan?	☐	☐	☐
Är rekryteringsmetoden lämpligt vald och genomförd?	☐	☐	☐
Finns det allvarliga brister som kan påverka tillförlitligheten?	☐	☐	☐

Kommentarer:

3. Datainsamling

Vilka metoder användes för datainsamling?

	Ja	Nej	Oklart
Finns det allvarliga brister i datainsamlingen som kan påverka tillförlitligheten?	☐	☐	☐

Kommentarer:

4. Analys

Vilka metoder användes för analys?

Stödfrågor för bedömning av brister i analyssteget:	Ja	Nej	Oklart
Är vald analysmetod lämplig och genomförd på ett lämpligt sätt?	☐	☐	☐
Var forskarna reflexiva vid tolkning av data?	☐	☐	☐
Validerades tolkningarna?	☐	☐	☐
Finns det allvarliga brister i analysen som kan påverka tillförlitligheten?	☐	☐	☐

Kommentarer:

5. Forskaren

Vilken bakgrund och kompetens hade forskarna?

Stödfrågor för bedömning av brister:	Ja	Nej	Oklart
Har forskarna någon relation till studiedeltagarna som kan påverka datainsamlingen?	☐	☐	☐
Har forskarna hanterat sin förförståelse på ett acceptabelt sätt?	☐	☐	☐
Var forskarna oberoende av finansiella eller andra förutsättningar som kunde påverka analysen?	☐	☐	☐
Finns det allvarliga brister som kan påverka tillförlitligheten?	☐	☐	☐

Kommentarer:

Online Supplementary File 5 – Quality Assessments

Quality assessment from the SBU checklist

Study	Data collection	Analysis	1. Theory/Philosophy: aim and research question coherent with theory or philosophical approach	2. Participants: relevant sampling; recruitment relevant and well described; ethical considerations relevant and well described	3. Data collection: relevant and well described	4. Data analysis: methodology relevant and well described; researchers showing reflexiveness in their analysis	5. The researcher: researcher–participant relationship well described; researcher pre-understanding well described; conflict of interest described	Methodological quality assessment
Barbosa *et al*[63]	Semi-structured focus groups	Thematic analysis	Yes	Yes	Yes/unclear	Yes	Yes	High
				Limitations: Ensure participants confidentiality and anonymity, but conducts focus groups where that cannot be ensured. Only one moderator for focus groups. Brief description of analysis.				
Boersma *et al*[60]	Focus groups	Deductive analysis from the RE-AIM framework	Yes	Yes	Yes/unclear	Yes	Yes/no	Moderate
				Limitations: Researcher-participant relation not described. Potential response bias discussed (with regards to focus group).				

Boström *et al*[49]	Focus group and individual semi-structured interviews	Qualitative content analysis	Yes	Yes	Yes	Yes	Yes/no	Moderate
			Limitations: Researcher-participant relation not described. Not discussing preunderstanding.					
Coyne[62]	Individual interviews with open-ended questions	Grounded theory	Yes	Yes	Unclear	Yes	Yes/no	Moderate
			Limitations: Researcher-participant relation not described. Split focus in the data between families/patients and HCPs.					
Fridberg *et al*[50]	Focus groups, semi-structured dyadic interviews, and semi-structured individual interviews	Deductive–inductive content analysis	Yes	Yes	Unclear	Yes	Yes	Moderate
			Limitations: Potential data collection bias.					
Kadri *et al*[56] (initial study: Rapaport *et al*[66])	Semi-structured individual interviews	Secondary qualitative thematic analysis	Yes	Yes	Unclear	Yes	Yes/no	Moderate
			Limitations: Initial study not having the precise focus relevant for this study which means data could have been more precise, the secondary analysis however relevant for this review. Preunderstanding not described.					
Karlsson *et al*[51]	Semi-structured individual interviews	Systematic text condensation	Yes	Yes	Yes	Yes	Yes/no	High
			Limitations: Declaration of interest not described.					

For peer review only

Kirkley *et al*[59]	Focus groups, telephone interview, individual interviews	Qualitative thematic analysis	Yes	Yes/no	Yes	Yes	Yes/no	Moderate
				Limitations: Potential selection bias in the face-to-face interviews. Participants worked in departments with good knowledge in PCC whereas in the telephone interviews the knowledge varied. Relation with participants not described.				
Kjörnsberg *et al*[53]	Open-ended informal individual interviews	Latent qualitative content analysis	Yes	Yes/no	Yes	Yes	Yes/no	Moderate
				Limitations: Small sample of only 6 RNs. Conflict of interest not described.				
Nilsson *et al*[52]	Individual semi-structured interviews	Qualitative content analysis	Yes	Yes	Yes	Yes	Yes/no	Moderate
				Limitations: Researcher-participant relationship not described. Preunderstanding not described.				
Pinkert *et al*[64]	Semi-structured focus groups	Secondary qualitative content analysis	Yes	Yes	Yes/unclear	Yes	Yes/no	Moderate
				Limitations: Cannot find researcher-participant relation due to not getting access to the initial study. Only focus groups.				
Ross *et al*[57]	Individual semi-structured interviews	Framework analysis	Yes	Yes	Yes	Yes	Yes/no	High
				Limitations: Researcher-participant relationship unclear and potential bias not described.				

Sjöberg and Forsner[54]	Focus groups, semi-structured individual interviews	Analysis in phenomenography	Yes	Yes/unclear	Yes	Yes	Yes/no	Moderate
				Limitations: Relatively low sample size (n = 7). Researcher-participant relationship not described.				
Uittenbroek *et al*[61]	Individual interviews with topic-based interview guide	Grounded theory	Yes	Yes/no	Yes	Yes	Yes/no	High
				Limitations: Only female participants. Researcher-participant relationship not described.				
Vassbø *et al*[65]	Individual open-ended interviews	Phenomenological-hermeneutical analysis for research of lived experiences	Yes	Yes/no	Yes	Yes	Yes/no	Moderate
				Limitations: Only female participants. Relationship between some researchers and participants that could lead to potential bias.				
Walker and Deacon[58]	Group interviews and an individual interview	Qualitative directed content analysis	Yes	Yes	Yes/no	Yes	Yes/no	Moderate
				Limitations: Researcher-participant relationship not described.				
Öhman *et al*[55]	Individual interviews with thematic interview guide	Grounded theory, qualitative content analysis	Yes	Yes	Yes	Yes	Yes/no	High
				Limitations: Preunderstanding not described. Researcher-participant relationship not described.				

Online Supplementary File 6 - Characteristics of included studies					
**Author, year, country**	**Aim**	**Data collection method**	**Analysis method**	**Context**	**HCPs**
Barbosa *et al*,[64] 2015, Portugal	To explore direct-care workers' experiences of a psychoeducational intervention including person-centred care	Semi-structured focus groups (mixed methods)	Thematic analysis	Four residential care facilities	Direct-care workers (*n* = 50) (8 focus groups) (all women)
Boersma *et al*,[61] 2017, The Netherlands	To explore how caregivers working with a person-centred care method (the Veder method) experience their job satisfaction	Focus groups (mixed methods)	Deductive analysis from the RE-AIM framework	Six nursing home wards	Professional caregivers (*n* = 42) (6 focus groups) (40 women, 2 men)
Boström *et al*,[50] 2014, Sweden	To describe how nurses specialising in diabetes care experience patient-centred care during an intervention for type 2 diabetes	Focus group and individual semi-structured interviews	Qualitative content analysis	Primary health care centres in northern Sweden	Diabetes specialist nurses (*n* = 10) (2 focus groups, 10 individual interviews, same participants) (all women)
Coyne,[63] 2015, Ireland	To explore families' and nurses' experiences and perceptions of family-centred care	Individual interviews using open-ended questions	Constructivist grounded theory	One general hospital ward and one surgical ward for children in a children's hospital, and one surgical	RNs (*n* = 18) (17 women, 1 man)

				ward for children in a general district hospital	
Fridberg *et al*,[51] 2021, Sweden	To describe HCPs' perceptions and experiences of PCC implementation	Focus groups, semi-structured dyadic interviews, and semi-structured individual interviews	Deductive–inductive content analysis	Six healthcare units in a region in central Sweden	97 in total<br>Assistant nurses (*n* = 30), RNs (*n* = 40), physicians (*n* = 10), physiotherapists (*n* = 9), occupational therapists (*n* = 7), 'other' (not specified) (*n* = 1)<br>(65 participants in 15 focus groups, 10 participants in 5 dyadic interviews, 22 individual interviews)<br>(80 women, 17 men)
Kadri *et al*,[57] 2018, UK (Initial study: Rapaport *et al*[67])	To explore home-care staff members' experiences and perceptions of their personhood when working with person-centred care	Semi-structured individual interviews	Secondary qualitative thematic analysis	Six care homes in the UK: three residential care homes and three nursing homes	Care home staff (*n* = 25)<br>(17 women, 8 men)
Karlsson *et al*,[52] 2019, Sweden	To describe RNs' job satisfaction	Semi-structured individual interviews	Systematic text condensation	One university hospital and one county hospital from different healthcare regions in Sweden	RNs (*n* = 25)<br>(23 women, 2 men)
Kirkley *et al*,[60] 2011, UK: England, Scotland	To explore cultural organisational perspectives in the provision of person-centred care	Focus groups, individual interviews (mixed methods)	Qualitative thematic analysis	Organisations in England and Scotland providing respite care	Frontline staff (*n* = 17)

Kjörnsberg *et al*,[54] 2010, Sweden	To investigate RNs' experiences of transitioning to patient-focused care	Open-ended informal individual interviews	Latent qualitative content analysis	A ward for infectious diseases at a university hospital in central Sweden	RNs (*n* = 6) (all women)
Nilsson *et al*,[53] 2019, Sweden	To explore RNs' experiences of working with person-centred care	Individual semi-structured interviews, focus groups	Qualitative content analysis	An acute ward at a university hospital in Sweden	14 in total RNs, enrolled nurses (12 women, 2 men)
Pinkert *et al*,[65] 2018, Austria and Germany	To describe the experiences of RNs working with dementia patients in acute-care hospital contexts	Semi-structured focus groups	Secondary qualitative content analysis	Acute-care settings in four Austrian hospitals and five German hospitals	RNs (*n* = 68) RNs from Austrian wards (*n* = 46) and German wards (*n* = 22) (12 focus groups)
Ross *et al*,[58] 2015, UK	To describe nurses' perspectives on facilitators of person-centred care	Individual semi-structured interviews	Framework analysis	An acute ward at a UK teaching hospital	14 in total RNs (*n* = 7), support workers (*n* = 3), student nurses (*n* = 4)
Sjöberg and Forsner,[55] 2020, Sweden	To explore physiotherapists' varying perceptions of person-centred care	Focus groups, semi-structured individual interviews	Analysis in phenomenography in accordance with Sjöström and Dahlgren (2002)	Wards for acute medicine, rheumatology, surgery, and orthopaedics	Physiotherapists (*n* = 7) (7 individual interviews, 1 focus group with 3 participants from individual interviews) (6 women, 1 man)
Uittenbroek *et al*,[62] 2018, The Netherlands	To explore case managers' perceptions of their professional role when working with person-centred care	Individual interviews using topic-based interview guide	Classical grounded theory	GP practices in northern Netherlands	11 in total RNs (*n* = 6), social workers (*n* = 5) (All women)

Vassbø *et al*,[66] 2019, Australia, Norway, and Sweden	To illuminate how HCPs in nursing homes experience the meaning of person-centred care focusing on their job satisfaction	Individual open-ended interviews	Phenomenological–hermeneutical analysis for research into lived experiences	Three nursing homes: one in a city in Norway, one in rural Sweden, and one in rural Australia	29 in total<br>RNs (*n* = 10), enrolled nurses (*n* = 12), care assistants (*n* = 2), personal carers (*n* = 2), occupational therapists (*n* = 2), physiotherapist (*n* = 1) (all women)
Walker and Deacon,[59] 2016, UK	To explore nurses' experiences of person-centred care of the suddenly bereaved	Group interviews, dyadic interview, and individual interview	Qualitative directed content analysis	A hospital with critical and acute-care services	RNs (*n* = 9)<br>(2 group interviews with 7 participants, 1 dyadic and 1 individual interview)
Öhman *et al*,[56] 2017, Sweden	To explore and improve knowledge of the factors contributing to HCPs' job satisfaction	Individual interviews using thematic interview guide (mixed methods)	Constructivist grounded theory, qualitative content analysis	Six elderly-care facilities in Sweden	17 in total<br>RNs (*n* = 5), physiotherapists (*n* = 6), occupational therapists (*n* = 6)<br>(14 women, 3 men)

only

BMJ Open

Healthcare professionals' experiences of job satisfaction when providing person-centred care: a systematic review of qualitative studies

Journal:	*BMJ Open*
Manuscript ID	bmjopen-2022-071178.R2
Article Type:	Original research
Date Submitted by the Author:	12-May-2023

Complete List of Authors:	Gustavsson, Kristoffer; University of Gothenburg Centre for Person-Centred Care<br>van Diepen, Cornelia; University of Gothenburg Centre for Person-Centred Care; Erasmus Universiteit Rotterdam Erasmus School of Health Policy and Management,<br>Fors, Andreas; University of Gothenburg Centre for Person-Centred Care; University of Gothenburg Institute of Health and Care Sciences<br>Axelsson, Malin; Malmö University Department of Care Science<br>Bertilsson, Monica; University of Gothenburg School of Public Health and Community Medicine<br>Hensing, Gunnel; University of Gothenburg School of Public Health and Community Medicine

**Primary Subject Heading**:	Occupational and environmental medicine
Secondary Subject Heading:	Patient-centred medicine, Public health, Qualitative research, Health services research, Mental health
Keywords:	PREVENTIVE MEDICINE, PUBLIC HEALTH, Quality in health care < HEALTH SERVICES ADMINISTRATION & MANAGEMENT, SOCIAL MEDICINE

SCHOLARONE™
Manuscripts

I, the Submitting Author has the right to grant and does grant on behalf of all authors of the Work (as defined in the below author licence), an exclusive licence and/or a non-exclusive licence for contributions from authors who are: i) UK Crown employees; ii) where BMJ has agreed a CC-BY licence shall apply, and/or iii) in accordance with the terms applicable for US Federal Government officers or employees acting as part of their official duties; on a worldwide, perpetual, irrevocable, royalty-free basis to BMJ Publishing Group Ltd ("BMJ") its licensees and where the relevant Journal is co-owned by BMJ to the co-owners of the Journal, to publish the Work in this journal and any other BMJ products and to exploit all rights, as set out in our licence.

The Submitting Author accepts and understands that any supply made under these terms is made by BMJ to the Submitting Author unless you are acting as an employee on behalf of your employer or a postgraduate student of an affiliated institution which is paying any applicable article publishing charge ("APC") for Open Access articles. Where the Submitting Author wishes to make the Work available on an Open Access basis (and intends to pay the relevant APC), the terms of reuse of such Open Access shall be governed by a Creative Commons licence – details of these licences and which Creative Commons licence will apply to this Work are set out in our licence referred to above.

Other than as permitted in any relevant BMJ Author's Self Archiving Policies, I confirm this Work has not been accepted for publication elsewhere, is not being considered for publication elsewhere and does not duplicate material already published. I confirm all authors consent to publication of this Work and authorise the granting of this licence.

For peer review only

Title: Healthcare professionals' experiences of job satisfaction when providing person-centred care: a systematic review of qualitative studies

Kristoffer Gustavsson (ORCID id: 0000-0002-1743-9576),[1] Cornelia van Diepen (0000-0001-6991-9443),[1,2] Andreas Fors (0000-0001-8980-0538),[1,3,4] Malin Axelsson (0000-0001-5493-8334),[5] Monica Bertilsson (0000-0003-2365-2522),[6] Gunnel Hensing (0000-0002-3457-2993)[6]

[1] Centre for Person-Centred Care (GPCC), University of Gothenburg, Gothenburg, Sweden

[2] Erasmus School of Health Policy & Management, Erasmus University Rotterdam, Rotterdam, Netherlands

[3] Institute of Health and Care Sciences, Sahlgrenska Academy, University of Gothenburg, Goteborg, Sweden

[4] Region Västra Götaland, Research, Education, Development and Innovation, Primary Health Care, Gothenburg, Sweden

[5] Department of Care Science, Faculty of Health and Society, Malmö University, Malmö, Sweden

[6] School of Public Health and Community Medicine, Institute of Medicine, Sahlgrenska Academy, University of Gothenburg, Gothenburg, Sweden

Corresponding author:
Andreas Fors
Andreas.fors@gu.se

University of Gothenburg, Sahlgrenska Academy, Institute of Health and Care Sciences
Arvid Wallgrens backe, building 1
Box 457, 405 30 Gothenburg, Sweden

Word count: 3982 (excluding title page, abstract, references, figures and tables)

For peer review only - http://bmjopen.bmj.com/site/about/guidelines.xhtml

ABSTRACT

Objectives: This qualitative systematic review aimed to explore and synthesise healthcare professionals' (HCPs) experiences of job satisfaction when providing person-centred care (PCC) in healthcare settings in Europe.

Method: This systematic review of qualitative studies was followed by a thematic synthesis applying an inductive approach. Studies concerning HCPs and different levels of healthcare in Europe were eligible for inclusion. The CINAHL, Pubmed, and Scopus databases were searched. Study titles, abstracts, and full texts were screened for relevance. Included studies were assessed for methodological quality using a quality appraisal checklist. Data were extracted and synthesised via thematic synthesis, generating analytical themes.

Results: Seventeen studies were included in the final thematic synthesis, and eight analytical themes were derived. Most studies were conducted in Sweden and the United Kingdom and were performed in hospitals, nursing homes, elderly care, and primary care. Thirteen of these studies were qualitative and four used a mixed-method design in which the qualitative part was used for analysis. HCPs experienced challenges adapting to a new remoulded professional role and felt torn and inadequate due to ambiguities between organisational structures, task-oriented care, and PCC. Improved job satisfaction was experienced when providing PCC in line with ethical expectations, patients and colleagues expressed appreciation, and team collaboration improved, while learning new skills generated motivation.

Conclusion: This systematic review found varied experiences among HCPs. Notably, the new professional role was experienced to entail disorientation and uncertainty; importantly, it also entailed experiences of job satisfaction such as meaningfulness, an improved relationship between HCPs and patients, appreciation, and collaboration. To facilitate PCC implementation, healthcare organisations should focus on supporting HCPs through collaborational structures, and resources such as time, space and staffing.

PROSPERO registration number: CRD42022304732

ARTICLE SUMMARY

Strengths and limitations of this study:

For peer review only - http://bmjopen.bmj.com/site/about/guidelines.xhtml

- This is the first systematic review synthesising qualitative research on healthcare professionals' experiences of job satisfaction when providing person-centred care across different healthcare settings in Europe.
- The methodology was thoroughly described, increasing replicability and reliability, and the researchers involved in this review had different professional backgrounds, i.e., nursing, sociology, and social medicine.
- The search strategy was extensive, included a broad range of terms, and covered several databases; a search specialist was consulted in the process.
- The search was limited to studies written in English and published in or since 2010, so some potentially relevant studies might have been missed.
- The included studies had different qualitative methodologies and theoretical approaches, which could affect interpretations and comparisons.

Keywords: healthcare professional, job satisfaction, person-centred care, qualitative synthesis, systematic review

For peer review only

INTRODUCTION

In Europe, healthcare professionals (HCPs) are at higher risk of exposure to psychosocial risk factors from their work environment than are several other occupations,[1] which can result in adverse health impacts on the individual, such as stress, fatigue, burnout, and physical and emotional demands.[2] The working environment affects job satisfaction, which is a mental state of satisfaction deriving from employees' assessment of their work situation.[3] It includes factors such as accomplishment, praise, social relations, advancement opportunities, and undertaking tasks in line with personal ethical values.[4,5] Low job control can decrease health and job satisfaction,[6] and high stress combined with decreased job satisfaction is associated with high turnover rates in healthcare.[7] With demands on European healthcare systems gradually increasing,[8] recruiting and retaining educated HCPs is difficult, threatening the sustainability of healthcare.[9] Moreover, an under-supported workplace with many stressors can lead to decreased quality of care for patients and increased emotional demands on HCPs.[10]

Simultaneously, European healthcare has been evolving to involve more patient participation.[11] Person-centred care (PCC) is a model of care deriving from an ethical standpoint in which patients' capabilities and needs are emphasised, and care is provided through collaboration between professionals, patients, and family.[12,13] Providing PCC involves some key principles, such as: initiating and focusing partnership by carefully listening to the patient's narrative, with the patient seen as an expert on her/his health; and co-creating a health plan covering the patient's experiences, capabilities, goals, expectations, and social support resources along with medical status, and modifying the health plan according to the circumstances.[12,14,15] PCC communication involves both verbal (e.g., open-ended questions, reflections, and summaries) and non-verbal communication (e.g., displaying welcoming and respectful body language).[14,16]

Working with PCC based on the patient's narrative helps professionals realise their expected ethical standards and provide high-quality care.[17] Working in a more person-centred way requires conducive organisational structure and support, leadership and training, and constructive collaboration within the interprofessional team.[18,19] Several concepts have been used to describe care centred around the patient and her/his family, such as patient centredness or patient-centred care[20,21] or relationship-centred,[22] family-centred,[23] and individualised care,[24] and varied settings require specific approaches of 'centredness' on the

For peer review only - http://bmjopen.bmj.com/site/about/guidelines.xhtml

patient and family.[25 26] PCC entails building a relationship in which patients are involved as active partners in their care and treatment and, more distinctly, emphasises a shift away from a model of care in which patients are often regarded as passive care recipients.[13 15]

In European healthcare, ethical stress and lack of resources are issues affecting HCPs' ability to offer the desired care of the required quality.[27] PCC has been proposed as a model of care with potential to help solve some of these problems, although evidence regarding PCC and its impact on HCP outcomes remains unclear.[28] PCC implementation being increasingly integrated into healthcare systems internationally will expose more HCPs to this model of care. Findings from this systematic review can help enhance the understanding of how HCPs' job satisfaction relates to PCC, and lead to further research and new relevant policies to improve working conditions in healthcare.

Research has primarily investigated the patient outcomes of PCC, showing, for example, increased self-efficacy, improved satisfaction with care, improved symptom control, and clinical outcomes characterised by shorter hospital stays and cost savings.[29] Five reviews of aspects of HCP outcomes and PCC have been identified,[30-35] which presented mixed findings. These reviews focused on specific professions, such as registered nurses (RNs), nurse aides, direct-care workers, and caregivers in residential care or nursing homes,[30-32 34 35] or included only quantitative studies.[30 31 33-35] No reviews have explored qualitative research on job satisfaction from providing PCC across healthcare professions and settings in Europe; this systematic review accordingly aimed to fill this research gap.

AIM

This qualitative systematic review aimed to explore and synthesise HCPs' experiences of job satisfaction when providing PCC in healthcare settings in Europe.

METHODS

Design

A systematic review of qualitative studies was conducted. The research question was 'How do healthcare professionals in Europe, when providing PCC, experience their job satisfaction?' A protocol following the PRISMA-P checklist was written in preparation for the systematic review and registered in PROSPERO on 23 January 2022 (ID: CRD42022304732).[36] A systematic search was executed on 21 December 2021 in the electronic databases CINAHL,

For peer review only - http://bmjopen.bmj.com/site/about/guidelines.xhtml

150 Pubmed, and Scopus, covering the main research areas relevant to this review. An updated search, using the same search strategy for studies published in the period between December 2021 – March 2023 was conducted in March 2023. Thematic synthesis with an inductive approach was used to analyse data obtained from the relevant studies.[37] The framework for Enhancing Transparency in Reporting the Synthesis of Qualitative Research (ENTREQ)[38] was used for reporting the synthesis (see Online Supplementary File 1).

Eligibility criteria

The eligibility criteria for the literature search in this systematic review are presented in table 1, and were based on the Population, Exposure, Outcome (PEO) framework.[39] The inclusion criterion for the population was HCPs, for exposure it was PCC, and for the outcome it was experiences of job satisfaction. Additionally, studies from all healthcare settings were acceptable, provided they were conducted in European countries with a healthcare system that could be identified according to Rosengren *et al.*[40] Studies had to be qualitative, peer-reviewed, ethically approved, written in English, and published in or since 2010. The included qualitative studies also include the qualitative, but no quantitative, parts of mixed-method studies. The exclusion criteria were quantitative studies and reviews.

Table 1 Eligibility criteria

Inclusion criteria	Exclusion criteria
• Healthcare professionals (Population)<br>• Person-centred care (Exposure)<br>• Experiences of Job Satisfaction (Outcome)<br>• All healthcare settings<br>• Studies conducted in European countries with identified healthcare systems according to Rosengren *et al.*[40]<br>• Qualitative, peer-reviewed, and ethically approved studies, written in English<br>• Published in or since 2010	• Quantitative studies<br>• Reviews

Search strategy

For peer review only - http://bmjopen.bmj.com/site/about/guidelines.xhtml

The search strategy was created by the research group with assistance from a librarian at the Biomedical Library at the University of (name withheld during the review process). It comprised keywords and Medical Subject Heading (MeSH) terms hierarchically structured to facilitate the inclusion of broader and more precise medical and health-related terms.[41] In accordance with the PEO framework, relevant search terms associated with HCPs, PCC, and job satisfaction were defined and put in their associated search blocks.[39] Within each block, the search terms were connected using the Boolean operator 'OR', and the blocks were connected with the Boolean operator 'AND'.[42] Along with the PEO blocks, two additional blocks were added. The first additional search block, 'countries', consisted of the European countries eligible for inclusion, similar to Rosengren *et al*[40] and their mapping of European countries having identifiable and eligible healthcare systems. Rosengren *et al* found 23 eligible European countries and identified three types of healthcare systems: Beveridge (n = 12), Bismarck (n = 10), and Out of Pocket (n = 1).[40] The second additional block was 'study design', which comprised different search terms for qualitative study designs and mixed-method studies (e.g., interviews and focus groups) to ensure that the search would identify all relevant studies of qualitative design. The search strategy is presented in Online Supplementary File 2.

Selection process

The selection of studies was conducted between 25 January 2022 until 11 March 2022. The 3754 records from the database searches were imported into Endnote for reference management and into Rayyan software for screening. The first (A1) and second author (A2) screened titles and abstracts blinded and independently for relevance in Rayyan using a screening tool (see Online Supplementary File 3).[43] The 'detect duplicates' option available in both programs was used to remove 872 duplicates.[44 45] The remaining 2882 studies were screened for title and abstract, and 2807 of those were excluded. Out of the 75 studies, one was not retrieved in full-text. In the second step of the selection process, the remaining 74 studies were downloaded and shared in an Endnote library. The studies were read in full text for relevance against the inclusion criteria by A1 and A2 independently in Endnote. All full-text studies were discussed in a meeting among A1, A2, and A3 until consensus was reached. Fifty six studies were excluded due to incorrect outcome, exposure, or design, or by being conducted in the wrong location, or not being written in English. Eighteen studies were

For peer review only - http://bmjopen.bmj.com/site/about/guidelines.xhtml

considered relevant to the review. After closer reading, one study was interpreted as related to working during the COVID-19 pandemic rather than to person-centred work; therefore, it was excluded at this stage after discussions. The selection process is presented in a PRISMA flow diagram (Figure 1).[46]

Figure 1. The selection of studies presented in a PRISMA flow diagram.[46]

For the updated literature search conducted in March 2023, the same electronic databases and search strategy were used, but the year of publication was limited to 2021-2023. The records were imported into Endnote and screened for relevance by A1. Two relevant studies were identified, which will be incorporated in the discussion section.

Quality assessment

The Swedish Agency for Health Technology Assessment and Assessment for Social Services (SBU) checklist for assessing methodological quality was used to assess the included studies after they were read in full text.[47] This checklist has five domains (i.e., theoretical framework, sampling, data collection, analysis, and researcher role) and 13 items integrated among the five domains (see Online Supplementary File 4).[48]

The studies were initially assessed by A1, and secondly independently assessed by A6, who is a senior researcher and Professor. Both A1 and A6 considered omitting studies indicating low methodological quality. All studies considered relevant were assessed as having moderate or high quality by both assessors. Items in the five domains were related to methodological choices, such as if the aim was coherent with regard to the theoretical stance, if the recruitment was suitable and well conducted, or if the method of analysis was appropriate and carried out properly. The items could be answered with “yes”, “no” or “unclear”. The combined item answers regarding whether it had serious methodological concerns affecting the reliability of the study resulted in a domain grading of “yes” “no” or “unclear” (see Online Supplementary File 4). The gradings from the five domains were then combined and given an overall rating of the study as follows: no or few concerns = high quality; moderate concerns = moderate quality; and high concerns = low quality, in line with the recommendations of SBU.[49] The studies’ ratings varied between moderate (n = 12) and high (n = 5) quality. The most common deficiencies were missing descriptions of the researcher’s role and pre-understanding, and how it might have affected the study results. No studies were assessed as

For peer review only - http://bmjopen.bmj.com/site/about/guidelines.xhtml

having low methodological quality, so no studies were excluded due to the quality assessment. The quality assessments are presented in Online Supplementary File 5.

Data extraction and synthesis

The qualitative findings extracted from the included studies were analysed via thematic synthesis. The synthesis followed the three stages described by Thomas and Harden.[37] The synthesis was conducted by A1 supported by A6. All authors then reviewed and discussed the synthesis and themes.

The first stage of the thematic synthesis was coding, which was conducted using NVivo software, in which the text was coded line by line to identify passages of text from the studies in line with the aim and research question of the review. Each text passage received a code describing its meaning. The coding was continuously scrutinised: it was revised with further coding if consistency was lacking, or progressed without revision if no issues emerged.[37]

The second stage involved inductively developing descriptive themes from the data. In this stage, a descriptive theme was formulated to cover the first code from the first study; either the following codes from the same study were grouped under that theme, if the codes were related in meaning and concept, or new descriptive themes were formulated when codes with new thematic concepts emerged. Codes from subsequent studies were then assigned to existing descriptive themes or new themes were formulated, if needed.[37]

The third stage of the process 'went beyond' the content of the primary studies and created a new understanding of the topic. New knowledge was created by utilising the descriptive themes generated and connecting them to the research question to produce analytical themes. The analytical themes were discussed by A1 and A6, and new themes were created if necessary and scrutinised again until they were considered to sufficiently describe the descriptive themes.[37]

Patient and public involvement

This study was conducted without patient or public involvement.

RESULTS

Characteristics of included studies

For peer review only - http://bmjopen.bmj.com/site/about/guidelines.xhtml

The study aimed to explore HCPs' experiences of job satisfaction when providing PCC. The characteristics of the 17 included studies are presented in Online Supplementary File 6. Seven studies were conducted in Sweden,[50-56] four in the United Kingdom (UK),[57-60] two in the Netherlands,[61 62] one in Ireland,[63] one in Portugal,[64] one in Austria and Germany,[65] and one in Sweden, Norway, and Australia.[66]

Most of the studies were qualitative (*n* = 13),[50-55 57-59 62 63 65 66] while four[56 60 61 64] used mixed methods. Eight studies[52 54 56-58 62 63 66] used individual interviews, three[61 64 65] used focus groups, and six[50 51 53 55 59 60] combined individual and focus-group interviews, of which two[51 59] also used dyadic interviews.

The total number of participants in the included studies was 459 (range 6–97).[51 54] Thirteen studies[50-57 61-64 66] reported the gender of participants, who were predominantly women (*n* = 351; 90%). The professions represented were RNs and specialist nurses (*n* = 211),[50-54 56 58 59 62 63 65 66] assistant nurses, students, enrolled nurses, caregivers, direct-care workers, frontline staff (*n* = 189),[51 53 57 58 60 61 64 66] physiotherapists (*n* = 23),[51 55 56 66] occupational therapists (*n* = 15),[51 56 66] physicians (*n* = 10),[51] professional support workers and social workers (*n* = 8),[58 62] and 'other', with the profession not being specified (*n* = 1).[51] Nine studies were conducted in hospitals, [51-55 58 59 63 65] six in elderly-care, residential-care, or nursing homes,[56 57 60 61 64 66] and two in primary-care or GP practices (an organisation of one or more General Practitioners).[50 62]

For peer review only - http://bmjopen.bmj.com/site/about/guidelines.xhtml

Synthesis

Twenty-four descriptive themes were derived from the codes, and eight analytical themes from the descriptive themes (Table 2). The analytical themes were generated by going beyond the content of the primary studies, representing interpretations of the included studies as a whole.[37]

Table 2 Themes from the qualitative synthesis

Analytical themes	Descriptive themes	Studies included in themes
HCPs feeling torn and inadequate	Conflict between organisational structures and PCC<br>Conflict between disease-oriented task care and PCC<br>Feelings of inadequacy and guilt	Coyne,[63] Fridberg *et al*,[51] Kadri *et al*,[57] Kjörnsberg *et al*,[54] Pinkert *et al*,[65] Uittenbroek *et al*,[62]
PCC demanding for HCPs	High workloads hamper the work with PCC<br>PCC not affecting or is negative for workload<br>Stressful due to lack of resources	Boersma *et al*,[61] Boström *et al*,[50] Coyne,[63] Fridberg *et al*,[51] Kadri *et al*,[57] Karlsson *et al*,[52] Kjörnsberg *et al*,[54] Ross *et al*,[58] Sjöberg and Forsner,[55] Uittenbroek *et al*,[62]
Remoulded professional role	Disorientation and uncertainties with new approach and routines<br>Loss of control	Boström *et al*,[50] Fridberg *et al*,[51] Kadri *et al*,[57] Sjöberg and Forsner[55]

For peer review only - http://bmjopen.bmj.com/site/about/guidelines.xhtml

Patients having considerable influence		
Providing PCC meaningful for HCPs	Improved job satisfaction<br>Meaningfulness – making a difference and doing the 'little extra'<br>In line with ethical expectations<br>Improved relationship between HCPs and patients<br>Self-care for improved care	Barbosa *et al*,[64] Boersma *et al*,[61] Fridberg *et al*,[51] Karlsson *et al*,[52] Kjörnsberg *et al*,[54] Nilsson *et al*, [53] Pinkert *et al*,[65] Ross *et al*,[58] Vassbø *et al*,[66] Öhman *et al*,[56]
HCPs feeling appreciated	Gratitude from patients makes working with PCC easier<br>Feeling valued and appreciated by colleagues	Barbosa *et al*,[64] Boersma *et al*,[61] Nilsson *et al*, [53] Uittenbroek *et al*,[62] Vassbø *et al*,[66] Öhman *et al*[56]
Improved team collaboration	Enhanced collaboration improves satisfaction and working context<br>Social support reduces stress and frustration	Boersma *et al*,[61] Kirkley *et al*,[60] Nilsson *et al*, [53] Uittenbroek *et al*,[62] Vassbø *et al*[66]
Workload and stress reduction	Improves flow and workload<br>Reduced stress at work<br>Sense of increased control and reduced anxiety	Barbosa *et al*,[64] Fridberg *et al*,[51] Kjörnsberg *et al*,[54] Vassbø *et al*[66]
Increased personal motivation and commitment	Feeling joy and energy<br>Thriving at work	Barbosa *et al*,[64] Fridberg *et al*,[51] Kadri *et al*,[57] Karlsson *et al*,[52] Vassbø *et al*,[66] Walker and Deacon[59]

For peer review only - http://bmjopen.bmj.com/site/about/guidelines.xhtml

Willingness and motivation to perform 'ideal'
PCC

For peer review only - http://bmjopen.bmj.com/site/about/guidelines.xhtml

Analytical themes

HCPs feeling torn and inadequate

Working in line with PCC was experienced as challenging and could make HCPs feel torn and inadequate. The reason for this was described as a conflict between the desire to perform personalised care fulfilling ethical and professional standards, organisational deficiencies in support and negotiation, and an organisational focus on effectiveness.[54 57 62 63 65] A perceived conflict between disease-oriented task care and PCC was described, with HCPs initially uncertain whether, for example, listening attentively was regarded as a task.[62] HCPs who experienced difficulties balancing PCC with the more task-oriented disease care perceived that they were blamed by others, resulting in feelings of guilt and inadequacy.[51 54]

PCC demanding for HCPs

HCPs described how spending time with patients when providing PCC could be demanding and be hampered by high workloads in the organisation.[50 52 55 63] HCPs found that PCC resulted in the same or even a greater workload than before.[51 54 58 62] HCPs also perceived that it could be stressful to work with PCC due to pressures from insufficient resources in terms of time, space, and staffing.[51 57 63] Some HCPs found that PCC did not influence their job satisfaction, especially when dealing with difficult behaviours of patients, such as when they did not respond well.[61]

Remoulded professional role

HCPs experienced a remoulded professional role when providing PCC. This could result in disorientation and uncertainty due to new structures, routines, and adaptation to new ways of working.[50 55] They perceived their expertise to be compromised and experienced a loss of control, with patients now having more influence over their own care.[55 57] Moreover, HCPs experienced doubt concerning parts of their new role, which was more conversation based than before and felt less valid than the usual, more hands-on approach.[55] Providing PCC could be frustrating with patients who were less communicative and more reserved[55] and in situations in which HCPs did not agree with patients' wishes regarding their care.[51]

Providing PCC meaningful for HCPs

When HCPs could provide PCC in line with ethical expectations and could collaborate with patients, they experienced increased meaningfulness and job satisfaction.[52 53 56 58 65 66] PCC provision was aligned with HCPs' standards and expectations regarding ethics and routines, such as providing equitable and inclusive care, discussing goals and writing health plans with

For peer review only - http://bmjopen.bmj.com/site/about/guidelines.xhtml

patients.[51] Spending more time with patients improved the relationship between HCPs and patients.[51 52 54 58 61 66] Additionally, HCPs described becoming more aware of the importance of their personal well-being when providing PCC, through its connection to patient well-being. HCPs taking better care of themselves could enable care that both patients and HCPs were content with.[64]

HCPs feeling appreciated

Although some HCPs found that PCC did not influence their job satisfaction when dealing with patients presenting difficult behaviours, others found that providing PCC instead helped in the care of these patients, causing them to show appreciation.[61] Patient expressions of gratification with the care were experienced as leading to increased job satisfaction and enhanced energy.[56 61 66] PCC was also described as a practice leading to appreciation from colleagues, as colleagues could understand and acknowledge one another more through collaboration and interaction, increasing satisfaction and meaningfulness at work.[53 62 64 66]

Improved team collaboration

The enhanced awareness, learning, and collaboration with colleagues and organisations derived from PCC was described as improving job satisfaction and the work environment.[61 62 66] HCPs valued the social support that emerged from working with PCC, fostering a supportive culture in which colleagues shared experiences and feelings, and recognised and relieved each other; this was experienced as increasing job satisfaction and reducing stress and frustration.[53 60 66]

Workload and stress reduction

HCPs who implemented PCC experienced a better workflow with new routines[51] and a calmer work environment.[66] Lower workload and reduced stress were experienced from working with PCC and being responsible for fewer patients.[54] Moreover, HCPs described increased control arising from enhanced independence in organising the work.[66] With PCC, HCPs could better deal with situations formerly experienced as stressful, which was described as reducing anxiety.[64]

Increased personal motivation and commitment

Motivation[57 66] and commitment[52 57 59 64] to continue providing PCC increased. The HCPs experienced enhanced joy from the improved connections with patients[51] and increased energy when they could meet patients' needs.[66] Moreover, HCPs working jointly with PCC,

For peer review only - http://bmjopen.bmj.com/site/about/guidelines.xhtml

helping one another and discussing the care, generated motivation and an increased striving to improve their current skills.[66]

DISCUSSION

This systematic review identified, assessed, and synthesised qualitative studies to explore HCPs' experiences of providing PCC by focusing on job satisfaction. In the updated literature search, two additional relevant qualitative studies by Allerby *et al*,[67] and Petersson *et al*[68] were identified. Findings from these studies were in line with findings from this systematic review and will be incorporated into the following discussion.

An interesting finding derived from the synthesis concerned the remoulded professional role. A study by Boström *et al*[69] of RNs' experiences of providing PCC over the phone highlighted that RNs experienced a need to adapt and remould their professional role when working with PCC. Remoulding the professional role was an iterative process in which they had to challenge their thinking and practice, which could generate insecurity;[69] despite being a challenging process, the RNs experienced this remoulding as expanding their professional role.[69] HCPs experiencing development of the professional role when working with PCC was also described in the study by Allerby *et al*.[67] In this systematic review, uncertainty towards the new professional role when providing PCC emerged due to the new practice and routines, and an experienced decrease in control and autonomy.[50 51 55 57] Having a system with a PCC culture with well-supported and committed HCPs is a prerequisite for implementing PCC.[70] Moreover, Moore *et al*[26] described how HCPs must adopt a new professional role when working with PCC. HCPs' integration of PCC is a process that requires time for reflecting on and adapting to the theory and practice of PCC for the specific context.[14] A more equal distribution of power, with patients being more involved and seen as experts in their own care, could be experienced as challenging for HCPs used to traditional care. Summer Meranius *et al*[71] argued that PCC could lead to reduced autonomy and negative health impacts for HCPs. To avoid negative health impacts, overcome the challenges of adapting, and instead expand the professional role, HCPs should be provided with time for the process and be encouraged to reflect on the theory and practice of PCC in their specific setting.

HCPs unable to provide the care desired from an ethical standpoint were also apparent. Similar to the finding of Juthberg and Sundin,[72] guilt and inadequacy could arise from being torn between striving to provide PCC, task-oriented care, and organisational structures.[51 54 55]

For peer review only - http://bmjopen.bmj.com/site/about/guidelines.xhtml

[57 62 63 65] This was also described in the study by Peterson *et al*,[68] where care structures requiring tasks being carried out in high tempo could inhibit PCC. A review by Güney *et al*[32] showed that the organisation and task-oriented care could be barriers to PCC and that improved support could modify organisational barriers. Ethical stress can also emerge from not being able to provide the care desired due to a lack of resources.[27] Furthermore, perceived stress and job dissatisfaction can increase healthcare turnover rates.[7] However, systematic organisational support when providing PCC can reduce stress of conscience,[73] indicating that systematic organisational support could serve as a preventive measure. Providing resources for HCPs working with PCC could thus potentially reduce stress of conscience and improve job satisfaction; in the long term, this might lead to reduced turnover, with HCPs being more satisfied with the care provided.

HCPs also experienced improved job satisfaction and meaningfulness when providing PCC, being engaged, and spending time with patients,[51-54 56 58 61 65 66] and they described reduced workload and stress from working with PCC.[51 54 66] The mixed experiences found in this review regarding engagement, workload, and stress align with Summer Meranius *et al*,[71] suggesting that engagement in patient care is essential for PCC and enhances the relationship between HCPs and patients; however, too much engagement and commitment can also be considered a risk.[71] The contrasting views of workload and stress may be mediated by organisational support. Whereas reduced stress seemed to be related to resources such as enhanced support, increased stress was related to a lack of resources in term of space, time, and staffing. Structures promoting collaboration with colleagues and more time with patients facilitate a conducive working environment, promoting PCC.[70] This underpins the benefits of supporting HCPs with sufficient resources to provide PCC. Moreover, it aligns with WHO's efforts to promote the co-creation of care in the interest of improved working conditions.[74]

The HCPs felt increased appreciation from colleagues when providing PCC.[53 62 64 66] Additionally, improved team collaboration when providing PCC was described as leading to enhanced satisfaction with the work environment.[53 61 62 66] The findings regarding those themes are well aligned with Montgomery *et al*,[75] who found a positive association between well-functioning teamwork and engagement. Similarly, Allerby *et al*[67] found that PCC increased satisfaction for HCPs from the improved work environment, communication and team collaboration. This suggests that collaboration in providing PCC could have a role in improving the health and well-being of HCPs.

For peer review only - http://bmjopen.bmj.com/site/about/guidelines.xhtml

As in the review by van den Pol-Grevelink,[31] increased thriving, motivation, and commitment were also apparent.[52 57 59 64 66] Keyko *et al*[76] found that HCPs engaged in their work will experience increased motivation and well-being, resulting in higher-quality care. Moreover, healthy HCPs are a prerequisite for the consistent provision of PCC.[77] HCPs increasingly valued their personal well-being, which meant that they could provide better, more satisfying care.[64] HCPs providing care that helps them thrive and be satisfied could mean healthier HCPs and decreased turnover in healthcare, facilitating the continuous provision of PCC.

Strengths and limitations

A strength of this review was that its authors had different professional backgrounds, with A1, A3, and A4 coming from nursing, A2 from sociology, and A5 and A6 from social medicine. In the selection and research process, pre-understanding was addressed through regular discussions in the group. Moreover, a librarian was consulted, several databases were searched, a broad range of terms was used, and the reference lists of the included studies were scrutinised. Also, two authors independently screened the titles, abstracts, and full texts for inclusion.

A systematic review was a suitable method for the present research purpose, since the aim was to explore and synthesise findings regarding a particular research question.[78] To demonstrate transparency, reduce the risk of bias, and enable replicability, a PRISMA protocol was published in PROSPERO before data collection, a PRISMA flow diagram was used, and data from extracted studies were presented in tables.[46 79-81] It was a strength that the synthesis strictly followed the steps described by Thomas and Harden,[37] since these have been proven viable for thematically synthesising qualitative experiences of healthcare implementations. The themes derived from the qualitative synthesis can be valuable as guidance for further research or can inform policy and practice concerning HCPs' job satisfaction.[78 82]

Some potentially relevant studies might not have been captured since only English-language studies, specific search terms, and studies published in or since 2010 were included. Recent studies are more likely to capture the current healthcare situation. Additionally, the literature search was performed on 21 December 2021, and studies published after that date were not included in the synthesis of this review. However, an updated search was conducted in March 2023 to capture relevant studies published after the initial literature search.

For peer review only - http://bmjopen.bmj.com/site/about/guidelines.xhtml

The search was delimited regarding the countries included in the eligibility criteria. Two studies were included despite being partly conducted in countries not included in the criteria, which could be considered a limitation. Although Vassbø *et al*[66] collected some of the data in Australia, two of the three countries represented in the data collection (i.e., Sweden and Norway) fit the inclusion criteria and the study contained rich data, so the study was included after discussions. The study by Pinkert *et al*[65] was conducted in Germany and Austria, with the latter not being included in the criteria; however, these two countries have similar healthcare systems and the working conditions in the dementia care and acute settings are similar, so this study was also deemed relevant in order to facilitate a comprehensive overview.[65 83] Additionally, the included studies applied different methodologies and theoretical approaches within qualitative research, which might have affected how the studies were interpreted and compared.

CONCLUSION

This qualitative systematic review identified HCPs experiences of job satisfaction when providing PCC in European healthcare settings. Noteworthy is the experiences of a new professional role, with its own demands and initially disorientation and uncertainty. Vitally, the new role came with experiences of job satisfaction related to meaningfulness, deepened relationship between HCPs and patients, appreciation from patients and colleagues, and enhanced team collaboration. The findings are in line with theoretical assumptions of PCC not mainly aiming at job satisfaction, but improved relations from partnership and meaningfulness. An implication of our findings are the possibility for healthcare organisations to improve the initial phase of implementing PCC. This could be achieved by facilitating structures promoting team collaboration, and resources such as time, space and staffing. Future studies with a longitudinal follow-up approach with recurrent data collection can contribute with a deeper understanding of the mechanisms involved in the process towards increased job satisfaction when PCC is implemented. It can also contribute with causal inferences not possible from qualitative or cross-sectional studies.

Author contributions

All study authors; K.G (A1), C.v.D (A2), A.F (A3), M.A (A4), M.B (A5), and G.H (A6), were involved in planning and developing the design of the study. K.G created the eligibility criteria and search strategy, which all authors provided feedback on in discussions. K.G

For peer review only - http://bmjopen.bmj.com/site/about/guidelines.xhtml

developed the protocol and screening tool, which was reviewed by A.F, and G.H, who provided comments and suggestions for improvements. K.G and C.v.D screened titles, abstracts, and full-text studies for inclusion, which was discussed with A.F in a meeting. Extraction and synthesis of the data was conducted by K.G, supported by G.H through discussions, and then reviewed by all authors providing comments and suggestions for revisions. Both K.G and G.H conducted the quality assessments. K.G drafted the first version of the manuscript with constructive input from C.v.D, A.F., M.A., M.B, and G.H. Throughout the research process, the manuscript and its analyses, interpretations and conclusions were continuously discussed and critically assessed by all authors. The final version of the manuscript was approved by all authors.

Acknowledgements

We wish to thank Linda Hammarbäck at the Biomedical Library for contributing to the search strategy.

Funding

This work was supported by grants from the AFA Insurance (no.190030), an organisation owned by Sweden's labour market parties, and the University of Gothenburg Centre for Person-Centred Care (GPCC), Sweden. GPCC is funded by the Swedish Government's grant for Strategic Research Areas, Care Sciences (Application to Swedish Research Council no. 2009–1088). The funders have no role in the design of the study, data collection, analysis or interpretation.

Competing interests

None declared.

Patient consent for publication

Not required.

Ethics approval

No formal ethics approval was needed since this review is based on publicly available data from previously published studies.

Provenance and peer review

Not commissioned; externally peer reviewed.

For peer review only

Data availability statement

Data sharing not applicable as no datasets were generated and/or analysed for this study. This is a systematic review, and all the data used are taken from previously published material.

Supplemental material

For peer review only

For peer review only - http://bmjopen.bmj.com/site/about/guidelines.xhtml

REFERENCES

1. The European Agency for Safety and Health at Work. Third European Survey of Enterprises on New and Emerging Risks (ESENER 3) https://osha.europa.eu/en/publications/third-european-survey-enterprises-new-and-emerging-risks-esener-3/view, 2019.

2. The National Institute for Occupational Safety and Health. Healthcare Workers: Work Stress & Mental Health 2016 [Available from: https://www.cdc.gov/niosh/topics/healthcare/workstress.html accessed 21 February, 2022.

3. Harrison DA, Newman DA, Roth PL. How Important Are Job Attitudes? Meta-Analytic Comparisons of Integrative Behavioral Outcomes and Time Sequences. *Academy of Management journal* 2006;49(2):305-25. doi: 10.5465/AMJ.2006.20786077

4. Broetje S, Jenny GJ, Bauer GF. The Key Job Demands and Resources of Nursing Staff: An Integrative Review of Reviews. *Frontiers in Psychology* 2020;11 doi: 10.3389/fpsyg.2020.00084

5. Bigliardi B, Ivo Dormio A, Galati F, et al. The impact of organizational culture on the job satisfaction of knowledge workers. *VINE Very informal newsletter on library automation* 2012;42(1):36-51. doi: 10.1108/03055721211207752

6. Portoghese I, Galletta M, Coppola RC, et al. Burnout and workload among health care workers: the moderating role of job control. *Saf Health Work* 2014;5(3):152-57. doi: 10.1016/j.shaw.2014.05.004 [published Online First: 2014/06/07]

7. Halter M, Boiko O, Pelone F, et al. The determinants and consequences of adult nursing staff turnover: a systematic review of systematic reviews. *BMC Health Services Research* 2017;17(1):824. doi: 10.1186/s12913-017-2707-0

8. WHO. Spending on health in Europe: entering a new era. https://apps.who.int/iris/bitstream/handle/10665/340910/9789289055079-eng.pdf, 2021.

9. The European Commission. Recruitment and Retention of the Health Workforce in Europe. https://ec.europa.eu/health/other-pages/basic-page/recruitment-and-retention-health-workforce-europe-2015_en, 2015.

10. The European Commission. Occupational health and safety risks in the healthcare sector. https://op.europa.eu/en/publication-detail/-/publication/b29abb0a-f41e-4cb4-b787-4538ac5f0238/language-en/format-PDF/source-251761322, 2010.

For peer review only - http://bmjopen.bmj.com/site/about/guidelines.xhtml

11. Swedish Institute for Standards. Patient involvement in health care - Minimum requirements for person-centred care. https://www.sis.se/en/produkter/health-care-technology/general/health-care-services-in-general/ss-en-173982020/, 2020.

12. Ekman I. The GPCC. *European Heart Journal* 2018;39(7):495-96. doi: 10.1093/eurheartj/ehy029

13. Ekman I. Practising the ethics of person-centred care balancing ethical conviction and moral obligations. *Nurs Philos* 2022;23(3):e12382. doi: 10.1111/nup.12382 [published Online First: 20220225]

14. Britten N, Moore L, Lydahl D, et al. Elaboration of the Gothenburg model of person-centred care. *Health Expectations* 2017;20(3):407-18. doi: https://doi.org/10.1111/hex.12468

15. Ekman I, Swedberg K, Taft C, et al. Person-Centered Care — Ready for Prime Time. *European Journal of Cardiovascular Nursing* 2011;10(4):248-51. doi: 10.1016/j.ejcnurse.2011.06.008

16. Ekman N, Moons P, Taft C, et al. Observable indicators of person-centred care: an interview study with patients, relatives and professionals. *BMJ Open* 2022;12(4):e059308. doi: 10.1136/bmjopen-2021-059308

17. Epstein RM, Fiscella K, Lesser CS, et al. Why The Nation Needs A Policy Push On Patient-Centered Health Care. *Health Affairs* 2010;29(8):1489-95.

18. Vennedey V, Hower KI, Hillen H, et al. Patients' perspectives of facilitators and barriers to patient-centred care: insights from qualitative patient interviews. *BMJ Open* 2020;10(5):e033449. doi: 10.1136/bmjopen-2019-033449

19. Chenoweth L, Jeon Y-H, Stein-Parbury J, et al. PerCEN trial participant perspectives on the implementation and outcomes of person-centered dementia care and environments. *International Psychogeriatrics* 2015;27(12):2045-57. doi: 10.1017/S1041610215001350 [published Online First: 2015/08/26]

20. Mead N, Bower P. Patient-centredness: a conceptual framework and review of the empirical literature. *Soc Sci Med* 2000;51(7):1087-110. doi: 10.1016/s0277-9536(00)00098-8

21. Epstein RM. The science of patient-centered care. *J Fam Pract* 2000;49(9):805-7.

22. Beach MC, Inui T, Relationship-Centered Care Research N. Relationship-centered care. A constructive reframing. *J Gen Intern Med* 2006;21 Suppl 1(Suppl 1):S3-S8. doi: 10.1111/j.1525-1497.2006.00302.x

23. Kuo DZ, Houtrow AJ, Arango P, et al. Family-centered care: current applications and future directions in pediatric health care. *Matern Child Health J* 2012;16(2):297-305. doi: 10.1007/s10995-011-0751-7

24. Weiner SJ. Contextualizing medical decisions to individualize care: lessons from the qualitative sciences. *J Gen Intern Med* 2004;19(3):281-85. doi: 10.1111/j.1525-1497.2004.30261.x

25. Hughes JC, Bamford C, May C. Types of centredness in health care: themes and concepts. *Med Health Care Philos* 2008;11(4):455-63. doi: 10.1007/s11019-008-9131-5 [published Online First: 20080409]

For peer review only - http://bmjopen.bmj.com/site/about/guidelines.xhtml

26. Moore L, Britten N, Lydahl D, et al. Barriers and facilitators to the implementation of person-centred care in different healthcare contexts. *Scand J Caring Sci* 2017;31(4):662-73. doi: 10.1111/scs.12376 [published Online First: 2016/11/08]

27. Aiken LH, Sloane DM, Bruyneel L, et al. Nurses' reports of working conditions and hospital quality of care in 12 countries in Europe. *International Journal of Nursing Studies* 2013;50(2):143-53. doi: https://doi.org/10.1016/j.ijnurstu.2012.11.009

28. van Diepen C, Fors A, Ekman I, et al. Associations between person-centred care and job strain, stress of conscience, and intent to leave among hospital personnel. *Journal of Clinical Nursing* 2022;31(5-6):612-22. doi: https://doi.org/10.1111/jocn.15919

29. Britten N, Ekman I, Naldemirci Ö, et al. Learning from Gothenburg model of person centred healthcare. *BMJ* 2020;370:m2738. doi: 10.1136/bmj.m2738

30. Rajamohan S, Porock D, Chang Y-P. Understanding the Relationship Between Staff and Job Satisfaction, Stress, Turnover, and Staff Outcomes in the Person-Centered Care Nursing Home Arena. *Journal of Nursing Scholarship* 2019;51(5):560-68. doi: https://doi.org/10.1111/jnu.12488

31. van den Pol-Grevelink A, Jukema JS, Smits CHM. Person-centred care and job satisfaction of caregivers in nursing homes: a systematic review of the impact of different forms of person-centred care on various dimensions of job satisfaction. *International Journal of Geriatric Psychiatry* 2012;27(3):219-29. doi: https://doi.org/10.1002/gps.2719

32. Güney S, Karadağ A, El-Masri M. Perceptions and experiences of person-centered care among nurses and nurse aides in long term residential care facilities: A systematic review of qualitative studies. *Geriatric Nursing* 2021;42(4):816-24. doi: https://doi.org/10.1016/j.gerinurse.2021.04.005

33. van Diepen C, Fors A, Ekman I, et al. Association between person-centred care and healthcare providers' job satisfaction and work-related health: a scoping review. *BMJ Open* 2020;10(12):e042658. doi: 10.1136/bmjopen-2020-042658 [published Online First: 2020/12/10]

34. Barbosa A, Sousa L, Nolan M, et al. Effects of Person-Centered Care Approaches to Dementia Care on Staff: A Systematic Review. *Am J Alzheimers Dis Other Demen* 2015;30(8):713-22. doi: 10.1177/1533317513520213 [published Online First: 2014/01/23]

35. Brownie S, Nancarrow S. Effects of person-centered care on residents and staff in aged-care facilities: a systematic review. *Clin Interv Aging* 2013;8:1-10. doi: 10.2147/cia.S38589 [published Online First: 20130103]

36. Shamseer L, Moher D, Clarke M, et al. Preferred reporting items for systematic review and meta-analysis protocols (PRISMA-P) 2015: elaboration and explanation. *BMJ : British Medical Journal* 2015;349:g7647. doi: 10.1136/bmj.g7647

37. Thomas J, Harden A. Methods for the thematic synthesis of qualitative research in systematic reviews. *BMC Medical Research Methodology* 2008;8(1):45. doi: 10.1186/1471-2288-8-45

38. Tong A, Flemming K, McInnes E, et al. Enhancing transparency in reporting the synthesis of qualitative research: ENTREQ. *BMC Medical Research Methodology* 2012;12(1):181. doi: 10.1186/1471-2288-12-181

For peer review only - http://bmjopen.bmj.com/site/about/guidelines.xhtml

39. Bettany-Saltikov J. How to do a systematic literature review in nursing : a step-by-step guide. 2. ed. ed: London : McGraw-Hill Education/Open University Press 2016.

40. Rosengren K, Brannefors P, Carlström E. Adoption of the concept of person-centred care into discourse in Europe: a systematic literature review. *Journal Of Health Organization And Management, 2021, Vol 35, Iss 9, pp 265-280* 2021;35(9):265-80. doi: 10.1108/JHOM-01-2021-0008

41. National Library of Medicine. Medical Subject Headings 2022 [Available from: https://www.nlm.nih.gov/mesh/meshhome.html accessed 11 February, 2022.

42. Sayers A. Tips and tricks in performing a systematic review--chapter 4. *Br J Gen Pract* 2008;58(547):136-36. doi: 10.3399/bjgp08X277168

43. Polanin JR, Pigott TD, Espelage DL, et al. Best practice guidelines for abstract screening large-evidence systematic reviews and meta-analyses. *Research Synthesis Methods* 2019;10(3):330-42. doi: https://doi.org/10.1002/jrsm.1354

44. Bramer WM, Giustini D, de Jonge GB, et al. De-duplication of database search results for systematic reviews in EndNote. *J Med Libr Assoc* 2016;104(3):240-43. doi: 10.3163/1536-5050.104.3.014

45. Ouzzani M, Hammady H, Fedorowicz Z, et al. Rayyan-a web and mobile app for systematic reviews. *Syst Rev* 2016;5(1):210. doi: 10.1186/s13643-016-0384-4 [published Online First: 2016/12/07]

46. Page MJ, McKenzie JE, Bossuyt PM, et al. The PRISMA 2020 statement: an updated guideline for reporting systematic reviews. *BMJ* 2021;372:n71. doi: 10.1136/bmj.n71

47. SBU. Appraisal of Studies with Qualitative Methodology. https://www.sbu.se/globalassets/ebm/bedomning_studier_kvalitativ_metodik.pdf, 2020.

48. SBU. Guidance for Assessment of Studies with Qualitative Methodology. https://www.sbu.se/globalassets/ebm/vagledning_granskning_studier_kvalitativ_metodik.pdf, 2020.

49. SBU. SBU:s metodbok. https://www.sbu.se/sv/metod/sbus-metodbok/?pub=48286#48490, 2020.

50. Boström E, Isaksson U, Lundman B, et al. Patient-centred care in type 2 diabetes - an altered professional role for diabetes specialist nurses. *Scand J Caring Sci* 2014;28(4):675-82. doi: 10.1111/scs.12092

51. Fridberg H, Wallin L, Tistad M. The innovation characteristics of person-centred care as perceived by healthcare professionals: an interview study employing a deductive-inductive content analysis guided by the consolidated framework for implementation research. *BMC Health Services Research* 2021;21(1):904. doi: 10.1186/s12913-021-06942-y

52. Karlsson AC, Gunningberg L, Bäckström J, et al. Registered nurses' perspectives of work satisfaction, patient safety and intention to stay - A double-edged sword. *J Nurs Manag* 2019;27(7):1359-65. doi: 10.1111/jonm.12816

53. Nilsson A, Edvardsson D, Rushton C. Nurses' descriptions of person-centred care for older people in an acute medical ward-On the individual, team and organisational levels'. *J Clin Nurs* 2019;28(7):1251-59. doi: https://doi.org/10.1111/jocn.14738

For peer review only - http://bmjopen.bmj.com/site/about/guidelines.xhtml

54. Kjörnsberg A, Karlsson L, Babra A, et al. Registered nurses' opinions about patient focused care. *Australian Journal of Advanced Nursing* 2010;28(1):35-44.

55. Sjöberg V, Forsner M. Shifting roles: physiotherapists' perception of person-centered care during a pre-implementation phase in the acute hospital setting - A phenomenographic study. *Physiother Theory Pract* 2020:1-11. doi: 10.1080/09593985.2020.1809042

56. Öhman A, Keisu BI, Enberg B. Team social cohesion, professionalism, and patient-centeredness: Gendered care work, with special reference to elderly care - a mixed methods study. *BMC Health Serv Res* 2017;17(1):381. doi: https://doi.org/10.1186/s12913-017-2326-9

57. Kadri A, Rapaport P, Livingston G, et al. Care workers, the unacknowledged persons in person-centred care: A secondary qualitative analysis of UK care home staff interviews. *PLoS One* 2018;13(7):e0200031. doi: 10.1371/journal.pone.0200031

58. Ross H, Tod AM, Clarke A. Understanding and achieving person-centred care: the nurse perspective. *J Clin Nurs* 2015;24(9):1223-33. doi: 10.1111/jocn.12662

59. Walker W, Deacon K. Nurses' experiences of caring for the suddenly bereaved in adult acute and critical care settings, and the provision of person-centred care: A qualitative study. *Intensive Crit Care Nurs* 2016;33:39-47. doi: 10.1016/j.iccn.2015.12.005

60. Kirkley C, Bamford C, Poole M, et al. The impact of organisational culture on the delivery of person-centred care in services providing respite care and short breaks for people with dementia. *Health Soc Care Community* 2011;19(4):438-48. doi: 10.1111/j.1365-2524.2011.00998.x

61. Boersma P, Dröes RM, Lissenberg-Witte BI, et al. Does working with the Veder Contact Method influence the job satisfaction of caregivers? A non-randomized controlled trial in nursing homes for people with dementia. *Int Psychogeriatr* 2017;29(12):2017-32. doi: 10.1017/S1041610217001582

62. Uittenbroek RJ, van der Mei SF, Slotman K, et al. Experiences of case managers in providing person-centered and integrated care based on the Chronic Care Model: A qualitative study on embrace. *PLoS One* 2018;13(11):e0207109. doi: 10.1371/journal.pone.0207109

63. Coyne I. Families and health-care professionals' perspectives and expectations of family-centred care: hidden expectations and unclear roles. *Health Expect* 2015;18(5):796-808. doi: 10.1111/hex.12104

64. Barbosa A, Nolan M, Sousa L, et al. Supporting direct care workers in dementia care: effects of a psychoeducational intervention. *Am J Alzheimers Dis Other Demen* 2015;30(2):130-8. doi: https://doi.org/10.1177/1533317514550331

65. Pinkert C, Faul E, Saxer S, et al. Experiences of nurses with the care of patients with dementia in acute hospitals: A secondary analysis. *J Clin Nurs* 2018;27(1):162-72. doi: 10.1111/jocn.13864

66. Vassbø TK, Kirkevold M, Edvardsson D, et al. The meaning of working in a person-centred way in nursing homes: a phenomenological-hermeneutical study. *BMC Nurs* 2019;18:45. doi: https://doi.org/10.1186/s12912-019-0372-9

67. Allerby K, Goulding A, Ali L, et al. Increasing person-centeredness in psychosis inpatient care: staff experiences from the Person-Centered Psychosis Care (PCPC) project. *BMC Health Services Research* 2022;22(1):596. doi: 10.1186/s12913-022-08008-z

For peer review only - http://bmjopen.bmj.com/site/about/guidelines.xhtml

68. Petersson Å, Hellström A, Assarsson J, et al. Following a standardised pathway: Healthcare professionals' perspectives on person-centred care within ERAS for patients with colorectal cancer. *Journal of Clinical Nursing* 2022;n/a(n/a) doi: https://doi.org/10.1111/jocn.16562

69. Boström E, Ali L, Fors A, et al. Registered nurses' experiences of communication with patients when practising person-centred care over the phone: a qualitative interview study. *BMC Nurs* 2020;19:54. doi: https://doi.org/10.1186/s12912-020-00448-4

70. Santana MJ, Manalili K, Jolley RJ, et al. How to practice person-centred care: A conceptual framework. *Health Expectations* 2018;21(2):429-40. doi: https://doi.org/10.1111/hex.12640

71. Summer Meranius M, Holmström IK, Håkansson J, et al. Paradoxes of person-centred care: A discussion paper. *Nursing Open* 2020;7(5):1321-29. doi: https://doi.org/10.1002/nop2.520

72. Juthberg C, Sundin K. Registered nurses' and nurse assistants' lived experience of troubled conscience in their work in elderly care--a phenomenological hermeneutic study. *Int J Nurs Stud* 2010;47(1):20-9. doi: 10.1016/j.ijnurstu.2009.06.001 [published Online First: 20090701]

73. Backman A, Sjögren K, Lövheim H, et al. The influence of nursing home managers' leadership on person-centred care and stress of conscience: A cross-sectional study. *BMC Nursing* 2021;20(1):200. doi: 10.1186/s12912-021-00718-9

74. WHO. WHO global strategy on integrated people-centred health services 2016-2026. https://interprofessional.global/wp-content/uploads/2019/11/WHO-2015-Global-strategy-on-integrated-people-centred-health-services-2016-2026.pdf, 2016.

75. Montgomery A, Spânu F, Băban A, et al. Job demands, burnout, and engagement among nurses: A multi-level analysis of ORCAB data investigating the moderating effect of teamwork. *Burnout Research* 2015;2(2):71-79. doi: https://doi.org/10.1016/j.burn.2015.06.001

76. Keyko K, Cummings GG, Yonge O, et al. Work engagement in professional nursing practice: A systematic review. *Int J Nurs Stud* 2016;61:142-64. doi: 10.1016/j.ijnurstu.2016.06.003 [published Online First: 2016/06/29]

77. Hower KI, Vennedey V, Hillen HA, et al. Implementation of patient-centred care: which organisational determinants matter from decision maker's perspective? Results from a qualitative interview study across various health and social care organisations. *BMJ Open* 2019;9(4):e027591. doi: 10.1136/bmjopen-2018-027591

78. Snyder H. Literature review as a research methodology: An overview and guidelines. *Journal of Business Research* 2019;104:333-39. doi: https://doi.org/10.1016/j.jbusres.2019.07.039

79. Booth A, Clarke M, Ghersi D, et al. An international registry of systematic-review protocols. *The Lancet* 2011;377(9760):108-09. doi: https://doi.org/10.1016/S0140-6736(10)60903-8

80. Xiao Y, Watson M. Guidance on Conducting a Systematic Literature Review. *Journal of Planning Education and Research* 2017;39(1):93-112. doi: 10.1177/0739456X17723971

81. Butler A, Hall H, Copnell B. A Guide to Writing a Qualitative Systematic Review Protocol to Enhance Evidence-Based Practice in Nursing and Health Care.

For peer review only - http://bmjopen.bmj.com/site/about/guidelines.xhtml

Worldviews on Evidence-Based Nursing 2016;13(3):241-49. doi: https://doi.org/10.1111/wvn.12134

82. Noyes J, Booth A, Cargo M, et al. Qualitative evidence. Cochrane Handbook for Systematic Reviews of Interventions2019:525-45.

83. The European Parliament. Health Care Systems in the EU: A Comparative Study. https://www.europarl.europa.eu/workingpapers/saco/pdf/101_en.pdf, 1998.

For peer review only

For peer review only - http://bmjopen.bmj.com/site/about/guidelines.xhtml

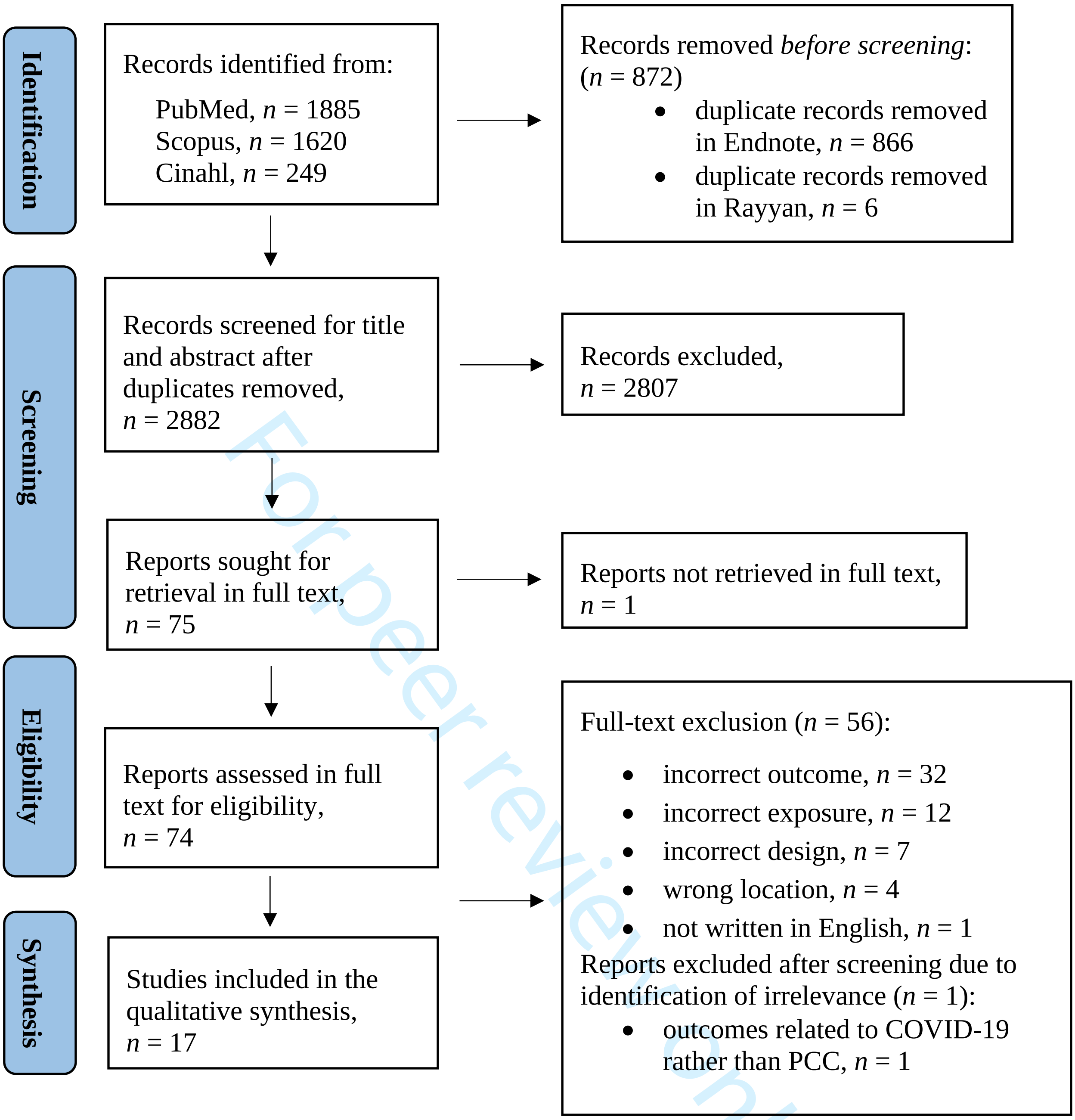

For peer review only

Online Supplementary File 1 – ENTREQ

No	Item	Guide and description	Reported on page #
**1**	Aim	State the research question the synthesis addresses.	5
**2**	Synthesis methodology	Identify the synthesis methodology or theoretical framework which underpins the synthesis, and describe the rationale for choice of methodology *(e.g. meta-ethnography, thematic synthesis, critical interpretive synthesis, grounded theory synthesis, realist synthesis, meta-aggregation, meta-study, framework synthesis).*	9
**3**	Approach to searching	Indicate whether the search was pre-planned (*comprehensive search strategies to seek all available studies)* or iterative (*to seek all available concepts until they theoretical saturation is achieved)*.	6
**4**	Inclusion criteria	Specify the inclusion/exclusion criteria *(e.g. in terms of population, language, year limits, type of publication, study type).*	6
**5**	Data sources	Describe the information sources used (e.g. *electronic databases (MEDLINE, EMBASE, CINAHL, psycINFO, Econlit), grey literature databases (digital thesis, policy reports), relevant organisational websites, experts, information specialists, generic web searches (Google Scholar) hand searching, reference lists)* and when the searches conducted; provide the rationale for using the data sources.	5-6
**6**	Electronic Search strategy	Describe the literature search *(e.g. provide electronic search strategies with population terms, clinical or health topic terms, experiential or social phenomena related terms, filters for qualitative research, and search limits).*	6-7
**7**	Study screening methods	Describe the process of study screening and sifting *(e.g. title, abstract and full text review, number of independent reviewers who screened studies).*	4
**8**	Study characteristics	Present the characteristics of the included studies *(e.g. year of publication, country, population, number of participants, data collection, methodology, analysis, research questions).*	7-8
**9**	Study selection results	Identify the number of studies screened and provide reasons for study exclusion *(e,g, for comprehensive searching, provide numbers of studies screened and reasons for exclusion indicated in a figure/flowchart; for iterative searching describe reasons for study exclusion and*	7-8

For peer review only

No	Item	Guide and description	Reported on page #
		inclusion based on modifications t the research question and/or contribution to theory development).	
**10**	Rationale for appraisal	Describe the rationale and approach used to appraise the included studies or selected findings *(e.g. assessment of conduct (validity and robustness), assessment of reporting (transparency), assessment of content and utility of the findings).*	8-9
**11**	Appraisal items	State the tools, frameworks and criteria used to appraise the studies or selected findings *(e.g. Existing tools: CASP, QARI, COREQ, Mays and Pope* [25]*; reviewer developed tools; describe the domains assessed: research team, study design, data analysis and interpretations, reporting).*	8
**12**	Appraisal process	Indicate whether the appraisal was conducted independently by more than one reviewer and if consensus was required.	8
**13**	Appraisal results	Present results of the quality assessment and indicate which articles, if any, were weighted/excluded based on the assessment and give the rationale.	8-9
**14**	Data extraction	Indicate which sections of the primary studies were analysed and how were the data extracted from the primary studies? *(e.g. all text under the headings 'results /conclusions' were extracted electronically and entered into a computer software).*	9
**15**	Software	State the computer software used, if any.	Endnote, NVivo, Rayyan
**16**	Number of reviewers	Identify who was involved in coding and analysis.	9
**17**	Coding	Describe the process for coding of data *(e.g. line by line coding to search for concepts).*	9
**18**	Study comparison	Describe how were comparisons made within and across studies *(e.g. subsequent studies were coded into pre-existing concepts, and new concepts were created when deemed necessary).*	9
**19**	Derivation of themes	Explain whether the process of deriving the themes or constructs was inductive or deductive.	9
**20**	Quotations	Provide quotations from the primary studies to illustrate themes/constructs, and identify whether the quotations were participant quotations of the author's interpretation.	N/A

For peer review only

No	Item	Guide and description	Reported on page #
**21**	Synthesis output	Present rich, compelling and useful results that go beyond a summary of the primary studies (e.g. *new interpretation, models of evidence, conceptual models, analytical framework, development of a new theory or construct).*	20-23

For peer review only

Online Supplementary File 2 – Search strategy

Search strategy

Population	Exposure	Outcome	Countries	Study design
“health personnel” “care personnel” “health care provider*” “care provider*” “health care worker*” “health care staff” “caregiver*” “care assistant*” “care aides” “nurse*” “nursing*” “physician*” “physiotherapist*” “physical therapist*” “psychologist*” “social worker*” “health care professional*” “nutritionist*” “case manager*” “medical staff” “general practitioner*”	“person centered” “person centeredness” “client centered” “patient centered” **“patient-centered care”** “relationship centered” “family centered” “patient focused” “person focused” “individualized care” “personalized care” “person-directed care” “individual centered”	**“perception*”** “experience*” “perspective*” **“attitude*”** “stress of conscience” **“job satisfaction” “absenteeism” “presenteeism” “occupational stress” “occupational health” “personnel turnover*” “sick leave” “stress, psychological”** “psychological stress” **“dyssomnia*”** “sleep disorder*” “sleep disturbance*” “moral stress” **“attitude of health personnel”**	“Sweden” “Swedish” “Norway” “Norwegian” “Finland” “Finnish” “Denmark” “Danish” “Iceland” “Icelandic” “United kingdom” “UK” “British” “North Ireland” “North Irish” “Wales” “Welsh” “Scotland” “Scottish” “Ireland” “Irish” “the Netherlands” “Dutch” “Germany” “German” “Spain” “Spanish” “Italy” “Italian” “Belgium” “Belgian” “Portugal” “Portuguese” “Malta” “Maltese” “Estonia” “Estonian” “Slovenia” “Slovenian” “Czech republic” “Czech” “France” “French” “Poland” “polish” “Hungary”	**“qualitative research”** “qualitative stud*” **“focus group*” “grounded theory”** “mixed-method” “ethnography” “phenomenology*” **“interviews as topic”** “interview*” **“hermeneutic*”**

	“Hungarian” “Greece” “Greek” “Latvia” “Latvian” “Serbia” “Serbian”

For peer review only

PubMed 1885

Scopus 1620

Cinahl 249

PubMed searched 21 December 2021

(Sweden OR Swedish OR Norway OR Norwegian OR Finland OR Finnish OR Denmark OR Danish OR Iceland OR Icelandic OR “United Kingdom” OR UK OR British OR North Ireland OR North Irish OR Wales OR Welsh OR Scotland OR Scottish OR Ireland OR Irish OR The Netherlands OR Netherlands OR Dutch OR Germany OR German OR Spain OR Spanish OR Italy OR Italian OR Belgium OR Belgian OR Portugal OR Portuguese OR Malta OR Maltese OR Estonia OR Estonian OR Slovenia OR Slovenian OR Czech Republic OR Czech OR France OR French OR Poland OR Polish OR Hungary OR Hungarian OR Greece OR Greek OR Latvia OR Latvian OR Serbia OR Serbian)

AND

(“health personnel”[MeSH] OR “health personnel”[tiab] OR “care personnel”[tiab] OR “health care provider”[tiab] OR “health care providers”[tiab] OR “care provider”[tiab] OR “care providers”[tiab] OR “health care worker”[tiab] OR “health care staff”[tiab] OR caregiver[tiab] OR caregivers[tiab] OR caregivers[mesh] OR “healthcare worker”[tiab] OR “healthcare workers”[tiab] OR “care assistant”[tiab] OR “care aides”[tiab] OR nurses[mesh] OR nurse[tiab] OR nurses[tiab] OR nursing[tiab] OR physicians[mesh] OR physicians[tiab] OR physician[tiab] OR physiotherapists[tiab] OR “physical therapists”[mesh] OR “physical therapists”[tiab] OR psychologist[tiab] OR “social workers”[mesh] OR “social workers”[tiab] OR “social worker”[tiab] OR “Health care professionals”[tiab] OR “health care professional”[tiab] OR “nutritionist”[tiab] OR nutritionists[mesh] OR “case managers” [tiab] OR “Medical staff”[mesh] OR “Medical staff”[tiab] OR “general practitioners”[tiab] OR “General Practitioners”[mesh])

AND

(“Person centered”[tiab] OR “person centred”[tiab] OR “person-centred”[tiab] OR “person centeredness”[tiab] OR “personcenteredness”[tiab] OR “person centredness”[tiab] OR “client centered”[tiab] OR “client centred”[tiab] OR “patient centered”[tiab] OR “patient centred”[tiab] OR “Patient-Centered Care”[Mesh] OR “relationship centered”[tiab] OR “relationship centred”[tiab] OR “family centred”[tiab] OR “patient focused”[tiab] OR “person

For peer review only

focused"[tiab] OR "individualized care" [tiab] OR "personalized care" [tiab] OR "person-directed care" [tiab] OR "individual centered"[tiab])

AND

(perception[mesh] OR perception[tiab] OR experience[tiab] OR experiences[tiab] OR perspective[tiab] OR attitude[mesh] OR attitude[tiab] OR attitudes[tiab] OR "stress of conscience"[tiab] OR "Job Satisfaction"[Mesh] OR "job satisfaction"[tiab] OR "Absenteeism"[Mesh] OR "absenteeism"[tiab] OR "presenteeism"[Mesh] OR "presenteeism"[tiab] OR "Occupational Stress"[Mesh] OR "occupational stress"[tiab] OR "Personnel Turnover"[Mesh] OR "personnel turnover"[tiab] OR "Sick Leave"[Mesh] OR "sick leave"[tiab] OR "Stress, Psychological"[Mesh] OR "psychological stress"[tiab] OR "Dyssomnias"[Mesh] OR "sleep disorder"[tiab] OR "sleep disorders"[tiab] OR "sleep disturbances"[tiab] OR "occupational health"[Mesh] OR "occupational health"[tiab] OR "moral stress"[tiab] OR "Attitude of Health Personnel"[mesh])

AND

("qualitative research"[mesh] OR "qualitative research"[tiab] OR "qualitative study"[tiab] OR "qualitative studies"[tiab] OR "focus groups"[mesh] OR "focus groups"[tiab] OR "focus group"[tiab] OR "Grounded theory"[mesh] OR "grounded theory"[tiab] OR "mixed-method"[tiab] OR ethnography[tiab] OR phenomenology*[tiab] OR interview*[tiab] OR "interviews as topic"[mesh] OR Hermeneutics[mesh] OR hermeneutic*[tiab])

Scopus searched 21 December 2021

(sweden OR swedish OR norway OR norwegian OR finland OR finnish OR denmark OR danish OR iceland OR icelandic OR "United Kingdom" OR uk OR british OR "North Ireland" OR "North Irish" OR wales OR welsh OR scotland OR scottish OR ireland OR irish OR the AND netherlands OR netherlands OR dutch OR germany OR german OR spain OR spanish OR italy OR italian OR belgium OR belgian OR portugal OR portuguese OR malta OR maltese OR estonia OR estonian OR slovenia OR slovenian OR "Czech Republic" OR czech OR france OR french OR poland OR polish OR hungary OR hungarian OR greece OR greek OR latvia OR latvian OR serbia OR serbian) AND TITLE-ABS-KEY ("health personnel" OR "care personnel" OR "health care provider*" OR "care provider*" OR "health care worker*" OR "health care staff" OR caregiver* OR "healthcare worker*" OR "care assistant*" OR "care

For peer review only

aides” OR nurse* OR nursing OR physician* OR physiotherapist* OR “physical therapist*” OR psychologist* OR “social worker*” OR “Health care professional*” OR “nutritionist*” OR “case manager*” OR “Medical staff” OR “general practitioner*”) AND TITLE-ABS-KEY (“Person centered” OR “person centred” OR “person centeredness” OR “personcenteredness” OR “person centredness” OR “client centered” OR “client centred” OR “patient centered” OR “patient centred” OR “Patient-Centered Care” OR “relationship centered” OR “relationship centred” OR “family centred” OR “patient focused” OR “person focused” OR “individualized care” OR “personalized care” OR “person-directed care” OR “individual centered”) AND TITLE-ABS-KEY (perception* OR experience* OR perspective* OR attitude* OR “stress of conscience” OR “Job Satisfaction” OR absenteeism OR presenteeism OR “Occupational Stress” OR “Personnel Turnover*” OR “Sick Leave” OR “psychological stress” OR dyssomnia* OR “sleep disorder*” OR “sleep disturbance*” OR “occupational health” OR “moral stress”) AND TITLE-ABS-KEY (“qualitative research” OR “qualitative stud*” OR “focus group*” OR “Grounded theory” OR “mixed-method” OR ethnography OR phenomenology OR interview* OR hermeneutic*) AND (LIMIT-TO (PUBYEAR , 2022) OR LIMIT-TO (PUBYEAR , 2021) OR LIMIT-TO (PUBYEAR , 2020) OR LIMIT-TO (PUBYEAR , 2019) OR LIMIT-TO (PUBYEAR , 2018) OR LIMIT-TO (PUBYEAR , 2017) OR LIMIT-TO (PUBYEAR , 2016) OR LIMIT-TO (PUBYEAR , 2015) OR LIMIT-TO (PUBYEAR , 2014) OR LIMIT-TO (PUBYEAR , 2013) OR LIMIT-TO (PUBYEAR , 2012) OR LIMIT-TO (PUBYEAR , 2011) OR LIMIT-TO (PUBYEAR , 2010))

Cinahl searched 21 December 2021

(Sweden OR Swedish OR Norway OR Norwegian OR Finland OR Finnish OR Denmark OR Danish OR Iceland OR Icelandic OR “United Kingdom” OR UK OR British OR “North Ireland” OR “North Irish” OR Wales OR Welsh OR Scotland OR Scottish OR Ireland OR Irish OR The Netherlands OR Netherlands OR Dutch OR Germany OR German OR Spain OR Spanish OR Italy OR Italian OR Belgium OR Belgian OR Portugal OR Portuguese OR Malta OR Maltese OR Estonia OR Estonian OR Slovenia OR Slovenian OR “Czech Republic” OR Czech OR France OR French OR Poland OR Polish OR Hungary OR Hungarian OR Greece OR Greek OR Latvia OR Latvian OR Serbia OR Serbian)

AND

((MH “health personnel+”) OR (TI “health personnel” OR AB “health personnel”) OR (TI “care personnel” OR AB “care personnel”) OR (TI “health care provider” OR AB “health care provider”) OR (TI “health care providers” OR AB “health care providers”) OR (TI “care provider” OR AB “care provider”) OR (TI “care providers” OR AB “care providers”) OR (TI “health care staff” OR AB “health care staff”) OR (TI caregiver OR AB caregiver) OR (TI caregivers OR AB caregivers) OR (MH caregivers+) OR (TI “healthcare worker” OR AB “healthcare worker”) OR (TI “healthcare workers” OR AB “healthcare workers”) OR (TI “care assistant” OR AB “care assistant”) OR (TI “care aides” OR AB “care aides”) OR (MH nurses+) OR (TI nursing OR AB nursing) OR (TI nurse OR AB nurse) OR (TI nurses OR AB nurses) OR (MH physicians+) OR (TI physicians OR AB physicians) OR (TI physician OR AB physician) OR (TI physiotherapists OR AB physiotherapists) OR (MH “physical therapists+”) OR (TI “physical therapists” OR AB “physical therapists”) OR (TI psychologist OR AB psychologist) OR (MH “social workers+”) OR (TI “social workers” OR AB “social workers”) OR (TI “social worker” OR AB “social worker”) OR (TI “Health care professionals” OR AB “Health care professionals”) OR (TI “health care professional” OR AB “health care professional”) OR (TI nutritionist OR AB nutritionist) OR (TI “case managers” OR AB “case managers”) OR (MH “Medical staff+”) OR (TI “Medical staff” OR AB “Medical staff”) OR (TI “general practitioners” OR AB “general practitioners”) OR (MH “Physicians, Family+”))

AND

((TI “Person centered” OR AB “Person centered”) OR (TI “person centred” OR AB “person centred”) OR (TI “person centeredness” OR AB “person centeredness”) OR (TI personcenteredness OR AB personcenteredness) OR (TI “person centredness” OR AB “person centredness”) OR (TI “client centered” OR AB “client centered”) OR (TI “client centred” OR AB “client centred”) OR (TI “patient centered” OR AB “patient centered”) OR (TI “patient centred” OR AB “patient centred”) OR (MH “Patient Centered Care”) OR (TI “relationship centered” OR AB “relationship centered”) OR (TI “relationship centred” OR AB “relationship centred”) OR (TI “family centred” OR AB “family centred”) OR (TI “patient focused” OR AB “patient focused”) OR (TI “person focused” OR AB “person focused”) OR (TI “individualized care” OR AB “individualized care”) OR (TI “personalized care” OR AB “personalized care”) OR (TI “person-directed care” OR AB “person-directed care”) OR (TI “individual centered” OR AB “individual centered”)) AND ((MH perception+) OR (TI perception OR AB perception) OR (TI experience OR AB experience) OR (TI

For peer review only

experiences OR AB experiences) OR (TI perspective OR AB perspective) OR (MH attitude+) OR (TI attitude OR AB attitude) OR (TI attitudes OR AB attitudes) OR (TI “stress of conscience” OR AB “stress of conscience”) OR (MH “Job Satisfaction+”) OR (TI “job satisfaction” OR AB “job satisfaction”) OR (MH Absenteeism+) OR (TI absenteeism OR AB absenteeism) OR (MH presenteeism+) OR (TI presenteeism OR AB presenteeism) OR (MH “Stress, Occupational+”) OR (TI “occupational stress” OR AB “occupational stress”) OR (MH “Personnel Turnover+”) OR (TI “personnel turnover” OR AB “personnel turnover”) OR (MH “Sick Leave+”) OR (TI “sick leave” OR AB “sick leave”) OR (MH “Stress, Psychological+”) OR (TI “psychological stress” OR AB “psychological stress”) OR (MH Dyssomnias+) OR (TI “sleep disorder” OR AB “sleep disorder”) OR (TI “sleep disorders” OR AB “sleep disorders”) OR (TI “sleep disturbances” OR AB “sleep disturbances”) OR (MH “occupational health+”) OR (TI “occupational health” OR AB “occupational health”) OR (TI “moral stress” OR AB “moral stress”) OR (MH “Attitude of Health Personnel+”))

AND

((MH “qualitative studies+”) OR (TI “qualitative research” OR AB “qualitative research”) OR (TI “qualitative study” OR AB “qualitative study”) OR (TI “qualitative studies” OR AB “qualitative studies”) OR (MH “focus groups+”) OR (TI “focus groups” OR AB “focus groups”) OR (TI “focus group” OR AB “focus group”) OR (MH “Grounded theory+”) OR (TI “grounded theory” OR AB “grounded theory”) OR (TI mixed-method OR AB mixed-method) OR (TI ethnography OR AB ethnography) OR (TI phenomenology* OR AB phenomenology*) OR (TI interview* OR AB interview*) OR (MH “interviews+”) OR (MH “Phenomenology”) OR (TI hermeneutic* OR AB hermeneutic*))

For peer review only

Online Supplementary File 3 – Screening Tool

Screening Tool

Citation, Title, and Abstract Screening

1. Does the **citation** indicate publication on or after 2010?

    a. Yes or Unsure/Unclear: continue screening

    b. No: stop screening

2. Does the **title or abstract** use English?

    a. Yes or Unsure/Unclear: continue screening

    b. No: stop screening

3. Does the **title or abstract** indicate that the study uses a qualitative design?

    a. Yes or Unsure/Unclear: continue screening

    b. No: stop screening

Keywords: qualitative, interview, focus group, grounded-theory, mixed-method, ethnography, phenomenology, hermeneutic.

4. Does the **title or abstract** indicate that the study concerns PCC?

    a. Yes or Unsure/Unclear: continue screening

    b. No: stop screening

Note: This also includes the different definitions of PCC, e.g., Patient-Centred Care, Relationship-Centred Care, Client-Centred Care etc. (See block 3 in Search Strategy).

5. Does the **title or abstract** indicate that the study concerns HCPs perspectives/experiences of PCC?

    a. Yes or Unsure/Unclear: continue screening

    b. No: stop screening

6. Does the **title or abstract** indicate that the research was conducted in one of the countries from the inclusion criteria?

a. Yes or Unsure/Unclear: continue screening

b. No: stop screening

Countries: Sweden, Norway, Finland, Denmark, Iceland, United Kingdom (England, Scotland, Wales, North Ireland) Ireland, The Netherlands, Germany, Spain, Italy, Belgium, Portugal, Malta, Estonia, Slovenia, Czech Republic, France, Poland, Hungary, Greece, Latvia, Serbia.

7. Does the **title or abstract** indicate that job satisfaction/occupational health in relation to PCC was studied?

a. Yes or Unsure/Unclear: continue screening

b. No: stop screening

Keywords: satisfaction, job satisfaction, job strain, stress, moral stress, stress of conscience, demand, occupational health

Decision: **Should this article be included for full-text read?**

a. **Yes**, all 7 screening questions answered Yes or Unsure/Unclear

b. **No**, at least one answers definitely “No”

review only

Online Supplementary File 4 – SBU Checklist

Bedömning av studier med kvalitativ metodik

UPPDATERAD 2020-10-06

Författare: ______________________ År: ______________

Granskare: ______________________________________

Sammanvägd bedömning av metodologiska brister:
Obetydliga eller mindre ☐<br>Måttliga ☐
Stora brister, studien ingår inte i syntesen ☐
Kommentarer:

only

1. Överensstämmelse mellan filosofisk hållning/teori och urval och metodik i studien[1]

Vilken teori eller filosofisk hållning utgick författarna från?

Hänger syfte och fråga ihop med teori/filosofisk hållning?	Ja	Nej	Oklart
	☐	☐	☐

Kommentarer:

2. Deltagare

Hur gjordes urvalet?

**Stödfrågor för bedömning av brister i urvalsförfarandet:**	Ja	Nej	Oklart
Är urvalet lämpligt för att besvara frågan?	☐	☐	☐
Är rekryteringsmetoden lämpligt vald och genomförd?	☐	☐	☐
Finns det allvarliga brister som kan påverka tillförlitligheten?	☐	☐	☐

Kommentarer:

3. Datainsamling

Vilka metoder användes för datainsamling?

Finns det allvarliga brister i datainsamlingen som kan påverka tillförlitligheten?	Ja	Nej	Oklart
	☐	☐	☐

Kommentarer:

4. Analys

Vilka metoder användes för analys?

**Stödfrågor för bedömning av brister i analyssteget:**	Ja	Nej	Oklart
Är vald analysmetod lämplig och genomförd på ett lämpligt sätt?	☐	☐	☐
Var forskarna reflexiva vid tolkning av data?	☐	☐	☐
Validerades tolkningarna?	☐	☐	☐
Finns det allvarliga brister i analysen som kan påverka tillförlitligheten?	☐	☐	☐

Kommentarer:

5. Forskaren

Vilken bakgrund och kompetens hade forskarna?

**Stödfrågor för bedömning av brister:**	Ja	Nej	Oklart
Har forskarna någon relation till studiedeltagarna som kan påverka datainsamlingen?	☐	☐	☐
Har forskarna hanterat sin förförståelse på ett acceptabelt sätt?	☐	☐	☐
Var forskarna oberoende av finansiella eller andra förutsättningar som kunde påverka analysen?	☐	☐	☐
Finns det allvarliga brister som kan påverka tillförlitligheten?	☐	☐	☐

Kommentarer:

Online Supplementary File 5 – Quality Assessments

Quality assessments from the SBU checklist

Study	Data collection	Analysis	1. Theory/Philosophy: aim and research question coherent with theory or philosophical approach	2. Participants: relevant sampling; recruitment relevant and well described; ethical considerations relevant and well described	3. Data collection: relevant and well described	4. Data analysis: methodology relevant and well described; researchers showing reflexiveness in their analysis	5. The researcher: researcher–participant relationship well described; researcher pre-understanding well described; conflict of interest described	Methodological quality assessment
Barbosa *et al*[64]	Semi-structured focus groups	Thematic analysis	Yes	Yes	Yes/unclear	Yes	Yes	High
				Limitations: Ensure participants confidentiality and anonymity, but conducts focus groups where that cannot be ensured. Only one moderator for focus groups. Brief description of analysis.				
Boersma *et al*[61]	Focus groups	Deductive analysis from the RE-AIM framework	Yes	Yes	Yes/unclear	Yes	Yes/no	Moderate
				Limitations: Researcher-participant relation not described. Potential response bias discussed (with regards to focus group).				

Boström *et al*[50]	Focus group and individual semi-structured interviews	Qualitative content analysis	Yes	Yes	Yes	Yes	Yes/no	Moderate
				Limitations: Researcher-participant relation not described. Not discussing preunderstanding.				
Coyne[63]	Individual interviews with open-ended questions	Grounded theory	Yes	Yes	Unclear	Yes	Yes/no	Moderate
				Limitations: Researcher-participant relation not described. Split focus in the data between families/patients and HCPs.				
Fridberg *et al*[51]	Focus groups, semi-structured dyadic interviews, and semi-structured individual interviews	Deductive–inductive content analysis	Yes	Yes	Unclear	Yes	Yes	Moderate
				Limitations: Potential data collection bias.				
Kadri *et al*[57]	Semi-structured individual interviews	Secondary qualitative thematic analysis	Yes	Yes	Unclear	Yes	Yes/no	Moderate
				Limitations: Initial study not having the precise focus relevant for this study which means data could have been more precise, the secondary analysis however relevant for this review. Preunderstanding not described.				
Karlsson *et al*[52]	Semi-structured individual interviews	Systematic text condensation	Yes	Yes	Yes	Yes	Yes/no	High
				Limitations: Declaration of interest not described.				

For peer review only

Kirkley *et al*[60]	Focus groups, telephone interview, individual interviews	Qualitative thematic analysis	Yes	Yes/no	Yes	Yes	Yes/no	Moderate
				Limitations: Potential selection bias in the face-to-face interviews. Participants worked in departments with good knowledge in PCC whereas in the telephone interviews the knowledge varied. Relation with participants not described.				
Kjörnsberg *et al*[54]	Open-ended informal individual interviews	Latent qualitative content analysis	Yes	Yes/no	Yes	Yes	Yes/no	Moderate
				Limitations: Small sample of only 6 RNs. Conflict of interest not described.				
Nilsson *et al*[53]	Individual semi-structured interviews	Qualitative content analysis	Yes	Yes	Yes	Yes	Yes/no	Moderate
				Limitations: Researcher-participant relationship not described. Preunderstanding not described.				
Pinkert *et al*[65]	Semi-structured focus groups	Secondary qualitative content analysis	Yes	Yes	Yes/unclear	Yes	Yes/no	Moderate
				Limitations: Cannot find researcher-participant relation due to not getting access to the initial study. Only focus groups.				
Ross *et al*[58]	Individual semi-structured interviews	Framework analysis	Yes	Yes	Yes	Yes	Yes/no	High
				Limitations: Researcher-participant relationship unclear and potential bias not described.				

Sjöberg and Forsner[55]	Focus groups, semi-structured individual interviews	Analysis in phenomenography	Yes	Yes/unclear	Yes	Yes	Yes/no	Moderate
				Limitations: Relatively low sample size (n = 7). Researcher-participant relationship not described.				
Uittenbroek *et al*[62]	Individual interviews with topic-based interview guide	Grounded theory	Yes	Yes/no	Yes	Yes	Yes/no	High
				Limitations: Only female participants. Researcher-participant relationship not described.				
Vassbø *et al*[66]	Individual open-ended interviews	Phenomenological-hermeneutical analysis for research of lived experiences	Yes	Yes/no	Yes	Yes	Yes/no	Moderate
				Limitations: Only female participants. Relationship between some researchers and participants that could lead to potential bias.				
Walker and Deacon[59]	Group interviews and an individual interview	Qualitative directed content analysis	Yes	Yes	Yes/no	Yes	Yes/no	Moderate
				Limitations: Researcher-participant relationship not described.				
Öhman *et al*[56]	Individual interviews with thematic interview guide	Grounded theory, qualitative content analysis	Yes	Yes	Yes	Yes	Yes/no	High
				Limitations: Preunderstanding not described. Researcher-participant relationship not described.				

Online Supplementary File 6 - Characteristics of included studies					
**Author, year, country**	**Aim**	**Data collection method**	**Analysis method**	**Context**	**HCPs**
Barbosa *et al*,[64] 2015, Portugal	To explore direct-care workers' experiences of a psychoeducational intervention including person-centred care	Semi-structured focus groups (mixed methods)	Thematic analysis	Four residential care facilities	Direct-care workers (*n* = 50) (8 focus groups) (all women)
Boersma *et al*,[61] 2017, The Netherlands	To explore how caregivers working with a person-centred care method (the Veder method) experience their job satisfaction	Focus groups (mixed methods)	Deductive analysis from the RE-AIM framework	Six nursing home wards	Professional caregivers (*n* = 42) (6 focus groups) (40 women, 2 men)
Boström *et al*,[50] 2014, Sweden	To describe how nurses specialising in diabetes care experience patient-centred care during an intervention for type 2 diabetes	Focus group and individual semi-structured interviews	Qualitative content analysis	Primary health care centres in northern Sweden	Diabetes specialist nurses (*n* = 10) (2 focus groups, 10 individual interviews, same participants) (all women)
Coyne,[63] 2015, Ireland	To explore families' and nurses' experiences and perceptions of family-centred care	Individual interviews using open-ended questions	Constructivist grounded theory	One general hospital ward and one surgical ward for children in a children's hospital, and one surgical	RNs (*n* = 18) (17 women, 1 man)

				ward for children in a general district hospital	
Fridberg *et al*,[51] 2021, Sweden	To describe HCPs' perceptions and experiences of PCC implementation	Focus groups, semi-structured dyadic interviews, and semi-structured individual interviews	Deductive–inductive content analysis	Six healthcare units in a region in central Sweden	97 in total<br>Assistant nurses (*n* = 30), RNs (*n* = 40), physicians (*n* = 10), physiotherapists (*n* = 9), occupational therapists (*n* = 7), 'other' (not specified) (*n* = 1)<br>(65 participants in 15 focus groups, 10 participants in 5 dyadic interviews, 22 individual interviews)<br>(80 women, 17 men)
Kadri *et al*,[57] 2018, UK	To explore home-care staff members' experiences and perceptions of their personhood when working with person-centred care	Semi-structured individual interviews	Secondary qualitative thematic analysis	Six care homes in the UK: three residential care homes and three nursing homes	Care home staff (*n* = 25)<br>(17 women, 8 men)
Karlsson *et al*,[52] 2019, Sweden	To describe RNs' job satisfaction	Semi-structured individual interviews	Systematic text condensation	One university hospital and one county hospital from different healthcare regions in Sweden	RNs (*n* = 25)<br>(23 women, 2 men)
Kirkley *et al*,[60] 2011, UK: England, Scotland	To explore cultural organisational perspectives in the provision of person-centred care	Focus groups, individual interviews (mixed methods)	Qualitative thematic analysis	Organisations in England and Scotland providing respite care	Frontline staff (*n* = 17)

Kjörnsberg *et al*,[54] 2010, Sweden	To investigate RNs' experiences of transitioning to patient-focused care	Open-ended informal individual interviews	Latent qualitative content analysis	A ward for infectious diseases at a university hospital in central Sweden	RNs (*n* = 6) (all women)
Nilsson *et al*,[53] 2019, Sweden	To explore RNs' experiences of working with person-centred care	Individual semi-structured interviews, focus groups	Qualitative content analysis	An acute ward at a university hospital in Sweden	14 in total RNs, enrolled nurses (12 women, 2 men)
Pinkert *et al*,[65] 2018, Austria and Germany	To describe the experiences of RNs working with dementia patients in acute-care hospital contexts	Semi-structured focus groups	Secondary qualitative content analysis	Acute-care settings in four Austrian hospitals and five German hospitals	RNs (*n* = 68) RNs from Austrian wards (*n* = 46) and German wards (*n* = 22) (12 focus groups)
Ross *et al*,[58] 2015, UK	To describe nurses' perspectives on facilitators of person-centred care	Individual semi-structured interviews	Framework analysis	An acute ward at a UK teaching hospital	14 in total RNs (*n* = 7), support workers (*n* = 3), student nurses (*n* = 4)
Sjöberg and Forsner,[55] 2020, Sweden	To explore physiotherapists' varying perceptions of person-centred care	Focus groups, semi-structured individual interviews	Analysis in phenomenography in accordance with Sjöström and Dahlgren (2002)	Wards for acute medicine, rheumatology, surgery, and orthopaedics	Physiotherapists (*n* = 7) (7 individual interviews, 1 focus group with 3 participants from individual interviews) (6 women, 1 man)
Uittenbroek *et al*,[62] 2018, The Netherlands	To explore case managers' perceptions of their professional role when working with person-centred care	Individual interviews using topic-based interview guide	Classical grounded theory	GP practices in northern Netherlands	11 in total RNs (*n* = 6), social workers (*n* = 5) (All women)

Vassbø *et al*,[66] 2019, Australia, Norway, and Sweden	To illuminate how HCPs in nursing homes experience the meaning of person-centred care focusing on their job satisfaction	Individual open-ended interviews	Phenomenological–hermeneutical analysis for research into lived experiences	Three nursing homes: one in a city in Norway, one in rural Sweden, and one in rural Australia	29 in total<br>RNs (*n* = 10), enrolled nurses (*n* = 12), care assistants (*n* = 2), personal carers (*n* = 2), occupational therapists (*n* = 2), physiotherapist (*n* = 1) (all women)
Walker and Deacon,[59] 2016, UK	To explore nurses' experiences of person-centred care of the suddenly bereaved	Group interviews, dyadic interview, and individual interview	Qualitative directed content analysis	A hospital with critical and acute-care services	RNs (*n* = 9)<br>(2 group interviews with 7 participants, 1 dyadic and 1 individual interview)
Öhman *et al*,[56] 2017, Sweden	To explore and improve knowledge of the factors contributing to HCPs' job satisfaction	Individual interviews using thematic interview guide (mixed methods)	Constructivist grounded theory, qualitative content analysis	Six elderly-care facilities in Sweden	17 in total<br>RNs (*n* = 5), physiotherapists (*n* = 6), occupational therapists (*n* = 6)<br>(14 women, 3 men)
